# Supplementary material for: Clinician and parent views on urine collection in precontinent children in the UK: a qualitative interview study
Source: BMJ Open. 2024 Apr 29;14(4):e081306. doi: 10.1136/bmjopen-2023-081306 (PMC11086548; doi:10.1136/bmjopen-2023-081306)
Supplement: Supplementary data [file bmjopen-2023-081306supp001.pdf]

| **SECTION A. RESEARCH DETAILS** | |
|---|---|
| **1. Full title of research** | Exploring perspectives, priorities, and solutions of urine collection: A qualitative user centric study to understand practices and gain feedback from healthcare professionals and parents/carers |
| **2. Short title of research** | Urine collection qualitative studies |
| **3. Principal Investigator (PI) / Student Supervisor** | Dr. Jeroen Bergmann |
| **4. PI's training in research ethics and/or GCP** | "Children and Clinical Research" from the Global Health Training Centre, Course completed on 31/08/2020 JB<br><br>"ICH Good Clinical Practice (GCP) E6", from the Global Health Training Centre, Course completed on 31/08/2020 JB<br><br>"Research Integrity", University of Oxford, Course completed on 31/08/2020 JB<br><br>"Research Integrity in Engineering and Technology", University of Oxford, Course completed on 26/02/2015 JB<br><br>"Introduction to Research Ethics" from the Global Health Training Centre, Course completed on 19/01/2015 JB<br><br>"Human Research", Basic Course, Collaborative Institutional Training Initiative, MIT, Completed on 12/11/2013 JB |
| **5. Student name and degree programme (if applicable)** | |
| **6. Department/Institute name** | Department of Engineering Science |
| **7. University email address** | jeroen.bergmann@eng.ox.ac.uk |
| **8. University telephone number** | |
| **9. Funding Source** | EPSRC IAA / NIHR BRC |
| **10. State any conflicts of interest and explain how these will be addressed** | None |

| **SECTION B. RESEARCHERS** | |
|---|---|
| | |
| **1. Researcher title and name** | Dr. Monica Armengol |
| **2. Department / Institute name** | Department of Engineering Science |
| **3. Role in research** | Researcher will obtain informed consent and conduct research activities with participant |
| **4. Training in research ethics and/or research integrity** | “Children and Clinical Research” from the Global Health Training Centre, Course completed on 23/06/2021 MA<br><br>“Research Integrity”, University of Oxford, Course completed on 24/06/2021 MA |
| **5. Researcher title and name** | Dr. Christopher Bird |
| **6. Department / Institute name** | MIC Oxford Health NHS Foundation Trust |
| **7. Role in research** | Design and supervision of project, liaise with medical professionals |
| **8. Training in research ethics and/or research integrity** | GCP up to date July/2021 (Health Research Authority, the governing body for NHS Ethics in the UK) |
| **9. Researcher title and name** | Dr. Gail Hayward |
| **10. Department / Institute name** | Primary Care Health Science |
| **11. Role in research** | Design and supervision of project |
| **12. Training in research ethics and/or research integrity** | GCP, 2019 (Health Research Authority, the governing body for NHS Ethics in the UK) |
| **13. Researcher title and name** | Dr. Philip Turner |
| **14. Department / Institute name** | Primary Health Care Science |
| **15. Role in research** | Design and supervision of project |
| **16. Training in research ethics and/or research integrity** | “Research Integrity”, University of Oxford, Course completed on 21/09/2021 |
| **17. Researcher title and name** | Nathaniel Mills |
| **18. Department / Institute name** | NIHR Children & Young People MedTech Cooperative |

| | |
|---|---|
| CUREC 1 Form Version 6.6 | Version/Date: V.02~~1~~ 21~~0~~.03~~10~~.2022~~1~~ |
| Ethics number: R77332/RE001 | |

| | |
|---|---|
| **19. Role in research** | Support and supervision. Liaise to medical professionals |
| **20. Training in research ethics and/or research integrity** | NIHR Good Clinical Practice course |
| **21. Researcher title and name** | Dr. Paul Dimitri |
| **22. Department / Institute name** | NIHR Children & Young People MedTech Cooperative |
| **23. Role in research** | Support and supervision. Liaise to medical professionals |
| **24. Training in research ethics and/or research integrity** | GCP (Health Research Authority, the governing body for NHS Ethics in the UK)<br>GCP Paediatrics Module (Health Research Authority, the governing body for NHS Ethics in the UK) |
| **25. Researcher title and name** | Gemma Wheler |
| **26. Department / Institute name** | NIHR Children & Young People MedTech Cooperative |
| **27. Role in research** | Design of study and analysis support |
| **28. Training in research ethics and/or research integrity** | NIHR Good Clinical Practice course |
| **29. Researcher title and name** | Molly Abbott |
| **30. Department / Institute name** | Oxford Health NHS Foundation Trust and University of Oxford |
| **31. Role in research** | Recruitment of Interviewees and performing interviews |
| **32. Training in research ethics and/or research integrity** | Oxford GCP training course for clinical researchers 28 March 2021 |

| **SECTION C. BASIC INFORMATION** | |
|---|---|
| **1. Provide a brief lay summary of the aims and objectives of the research. This should cover the questions it will answer and any potential benefits.**<br>**(Maximum 300 words)** | Urine is used to diagnose and monitor different diseases including diabetes and urine tract infections (UTI). Paediatric UTI accounts for 14% of the emergency department visits. Despite the high occurrence, collection of urine samples in non-toilet trained infants is challenging, lengthy and up to 30% of urine samples are contaminated with bacteria from the back passage or skin, making results meaningless.<br>Re-testing urine samples due to contamination causes delays, increases visits to the GP and emergency services. Additionally, it is associated with misdiagnosis, which can cause bacterial resistance if children are prescribed an antibiotic due to erroneous diagnosis.<br>This study aims to understand the current practices of urine collection in non-toilet trained infants and the challenges faced when carrying out, ordering or waiting for a collection. |

CUREC 1 Form Version 6.6 Version/Date: V.02~~1~~ 21~~0~~.03~~10~~.2022~~1~~
Ethics number: R77332/RE001

| | |
|---|---|
| | These findings will guide the focus areas for the development of a new device for paediatric urine collection, requirements, and specifications of the device.<br><br>We believe that a user-centric approach in the development of medical devices, produces device of higher clinical value. Participants will provide input in two parts: (1) interviews will aim to explore practices, costs, experiences, and perceptions of available diagnostic technology used for urine collection in infants in clinical settings. Additionally, we will explore unmet needs with regards to urine collection technologies and the potential impact of new diagnostic methods, tools or technology.<br>(2) a co-design workshop will allow participants to have input into possible solutions to the urine collection issues reported during the interviews. |
| **2. List all places where research will be conducted (including any other countries and online)** | Most interviews and workshop will be conducted online, via teams/zoom. Interviews will be conducted out of NHS premises. |
| **3. Anticipated research start date** | As soon as ethics is approved. Oct-Nov 2021 |
| **4. Anticipated research end date**<br>(n.b. A maximum of 5 years approval can be granted) | 3 years after approval |
| **5. Please list any CUREC Approved Procedure(s) you will follow** | |
| **6. Please list any CUREC Best Practice Guidance used to develop your research** | BPG 01 Researcher Safety/ BPG 03 Elite and expert interviewing/ BPG 09 Data collection, protection and management/ BPG 10 Conducting research interviews |
| **7. Name of departmental / peer reviewer (if applicable)** | Engineering Science / Sarah Hoeksma |

| **SECTION D. PARTICIPANTS** | |
|---|---|
| **1. Age range of participants** | Between 18 and 70 years old |
| **2. Are research participants classed as people whose ability to give free and informed consent is in question?** | Yes |
| **3. Anticipated number of participants** | Around 30 healthcare professionals and 20-30 parents |

| | |
|---|---|
| **4. How was the number of participants decided?** | We anticipate conducting between 20-30 interviews to healthcare professionals and 20-30 interviews to parents/carers. However, recruitment will continue until data saturation is reached or sufficient exploratory power for the findings is achieved. Further interviews might be required to fully explore the topics proposed. The research team will decide on concluding interviews.<br><br>For the workshop, we anticipate around 30 people taking part. This will include parents and healthcare professionals that took part in the interviews, in addition to other parents, health care professionals, engineers, designers and general public that are interested in taking part. |
| **5. Inclusion criteria** | Healthcare Professionals<br><br>Consenting adults with experience working with urine collection whether direct or indirect (perform, assist, manage, purchase, order or interpret) within a clinical setting. These include medical professionals, nurses, administrative staff and bioanalyst.<br><br>Parents/Carers:<br><br>Consenting adults with experience aiding a pre-continent child collect a urine sample in the last 2 years.<br><br>The adults taking part in the interviews, both parents/carers and healthcare professionals, must be able to take part on the interview conducted in English and consent to the interview being recorded. Participants must be over the age of 18 years and below 70 years old.<br><br>Co-design workshop<br><br>Consenting adults over the age of 18 years and below 70 years old with an interest in urine collection in infants, including those that took part in the interviews. Additionally, engineers, designers, industry experts and academics will be invited. |
| **6. Exclusion criteria** | The participant may not enter the study if they are unable to take part in an interview in English or written consent cannot be provided. |
| **7. Please mark 'X' against all planned recruitment methods**<br><br>Provide copies of all recruitment material for review | Poster advert ☒<br>Flyer ☒<br>Email circulation ☒<br>In-person approach ☒<br>Website ☒<br>Social media (e.g. twitter, Facebook) ☒<br>Snowball sampling (recruiting through contacts of existing participants) ☒<br>Newspapers ☐<br>Research recruitment sites (e.g. Prolific Academic, Amazon Turk) ☐<br>Existing departmental contacts or volunteer database ☒<br>Other (please specify) ☐ |
| **8. How will potential participants be identified and approached?** | Information about the study and poster will be shared in social media (including Facebook groups [with permission of the group moderator] i.e. Community and parents groups, for example [Oxford] Community, [Oxford] Parents & Careers, paediatric organisations, charities and research groups), twitter (personal |

| | |
|---|---|
| | accounts, research group account and organisations we are collaborating with – NIHR Children and Young People (CYP) Medical Technology and In-vitro Co-operative (MIC)/ NIHR Community Health (CH) MIC), blogs.<br><br>Newsletters and mailing lists could be used to share information and poster. Poster will include detail of £20 remuneration in the form of electronic gift vouchers will be offered for time taken to participate in interview.<br><br>These will include the University Department's mailing list charities and baby groups, paediatric medical organisations and, partners from the NIHR MICs will share information in their website and newsletter. The emails will be sent on behalf of the researchers conducting this study. We will also publish information of the study in our website and rely on word of mouth of participants.<br><br>Participants can express their interest in taking part of the study by contacting the researcher by email or phone. We will also include a form on the website for people to express their interest. Any potential participant expressing interest in taking part in the study will be contacted by email or telephone. Potential participants will be directed to our website where they can download the participant information sheet and consent form. Participants wishing to take part will be asked to return the consent form and participant information sheet signed. Participants wishing to take part, will be invited (by email or phone) to take part in the co-design workshop. Information and invite in the co-design workshop will use the same medium as the interviews did for distribution.<br><br>Participants will be given options regarding the timing, format, and venue (if face-to-face) for the interview will be given. The participant will have the opportunity to ask any questions either by email or telephone. They may choose to decline any further contact; in which case they will be reassured that this will in no way impact upon their current or future working.<br><br>The workshop will have a set date, participants will be asked to register their attendance in advance. |
| **9. Will informed consent be obtained from the research participants or their parents/ guardians?** If not, please explain why not. | Verbal consent will be taken at the start of interviews and co-design workshop. Before the interview/workshop starts, we will explain the study to the participant and go through the consent form.<br><br>Written informed consent will be obtained by means of participant dated signature and dated signature of the person who presented and obtained the Informed Consent.<br><br>Written consent will be sought participants will be given the option to fill the consent themselves and emailing it to us, they can also complete it through a GDPR compliant platform (https://www.smartsurvey.co.uk/) or researcher can fill them on their behalf and email a password-protected copy and retaining a copy for the records. For interviews being done by phone, form will be sent by email, or if not possible, oral consent will be sought.<br><br>Participant will be able to ask any questions or withdraw at any point without any explanation. |
| **10. For each activity or group of participants, explain how informed consent will be obtained from the participants themselves and/or their** | Verbal consent will be taken at the start of the interview and workshop. Study will be explained and the researcher will go through the consent form with the participant.<br><br>Written consent will be sought prior to interviews and co-design workshop are taking place. As interview/workshop will be conducted remotely (either online or |

| | |
|---|---|
| **parents/guardians, if applicable. How will their consent be recorded?** | by phone) participants will be given a few options to fill the consent form; the option to fill the consent themselves and emailing it to us, they can fill it online through a GDPR compliant software (https://www.smartsurvey.co.uk/ ) or researcher can fill them on their behalf and email a password-protected copy and retaining a copy for the records. A copy of the consent form will be made and stored safely in password protected file.<br><br>Participants will be given the opportunity to ask questions or withdraw from study. If participants are happy to proceed, interviews will be arranged online or by telephone at their convenience. A date will be set for the workshop, participants can register their interest to participants through the website. We will be available to reply to any questions or concerns that the participant may have at any point during this process. |

## SECTION E. RESEARCH METHODOLOGY

**1. Please mark 'X' against the methods that will be used in your research**

Ensure you address each method you will use in your informed consent documents and on this form

| | | | |
|---|---|---|---|
| Use of casual or local workers (e.g. interpreters) | ☐ | Audio recording of participant | ☒ |
| Interview (refer to guidance in BPG 10: Conducting research interviews) | ☒ | Video recording of participant | ☐ |
| Focus group | ☐ | Photography of participant | ☐ |
| Participant completes questionnaire in hard copy | ☐ | Physiological recording from participant | ☐ |
| Participant completes online questionnaire or other online task (refer to guidance in BPG 06: Internet-mediated research) | ☐ | Taking a sample of blood or other bodily fluid from a participant | ☐ |
| Use of social media to recruit or interact with participants (refer to guidance in BPG 06: Internet-mediated research) | ☒ | Participant observation | ☐ |
| Analysis of existing records | ☐ | Covert observation | ☐ |
| Participant performs verbal or aural task | ☒ | Systematic observation | ☐ |
| Participant performs paper and pencil task | ☒ | Observation of specific organisational practices | ☐ |
| Participant performs computer based task | ☒ | Other (please specify below) | ☐ |
| Measurement/recording of motor behaviour | ☐ | | |

**2. Provide a lay description of the research design and methods. In particular, describe clearly what participants in the research will be asked to do.**

Interviews:

Before interview starting, the information sheet will be reviewed, and any questions addressed.

- Participants will be asked to provide their informed consent to participation prior to data collection. Participants will be explained to that their participation is voluntary and they can end or withdraw from study at any point without any explanation.



Armengol M, *et al. BMJ Open* 2024; 14:e081306. doi: 10.1136/bmjopen-2023-081306

- A date for the interview to take place will be scheduled. Participants will have a choice of date, time and place of interview (online or telephone).
- Sessions will last between 40-60 min. Each participant will have one session. Consent will be asked in case follow-up is required in any of the questions.
- A qualitative methodology will be used given that it is appropriate to explore people's knowledge, choices, and decision. Data collection and analysis will be guided by the principles of grounded theory which allows adjustments to questions and sampling.
- Semi-structured questionnaires with open-ended questions will be used for the interviews. A topic guide will be used to conduct the interviews. This will allow flexibility in the order and wording of the questions. Additionally, questions will be amended based on on-going interviews, to explore emerging ideas and themes and to include new participants as participants for interview (theoretical sampling).

The topic guide will be divided into three parts, a warmup section that will include personal information and questions to get to know the person being interviewed and their relationship to urine collection. Then the main questions section, which will determine experiences, stories and opinions about urine collection methods. Finally, the wrap-up questions, to give the opportunity to ask any questions to us.

The open-ended questions will be used to probe the different areas of urine collection we are interested in. The interviews will explore the procedure, i.e., who uses it, device used, procedure (who collects the urine, how long it takes, final sample), post-collection feedback and process, patient follow-up.

- Interviews will be audio recorded and transcribed. If only audio is not possible (during online meeting),participant will be asked to turn off their video off during the interview..

Co-design Workshop

- Participants will be invited to take part in the workshop. They can express their interest by filling a registration form on the website.
- Participant will be able to download the consent and participant information form from the website. And will be able to contact the researchers with questions using the contact information provided in the participant information sheet.
- Participants will be asked to provide their informed consent to participation before the workshop starts. Participants will be explained to that their participation is voluntary and they can end or withdraw from study at any point without any explanation. They will also have the opportunity to ask questions.
- Participants will be divided in groups, where they can discuss urine collection ideas and brainstorm new solutions. This can be by drawing/writing/verbal explanation or making something with available materials. At the end each group will be able to present the summary of their ideas to the rest of the participants and organisers.
- If workshops are held online. An online worksheet will be set up and teams will work on their ideas there. Teams will be asked to present their ideas at the end of the workshop.
- Notes will be taken and online worksheets will be kept by the research team for analysis and further work. No video or audio recording will be taken.
- Session will last between 2.5-3h



Armengol M, *et al. BMJ Open* 2024; 14:e081306. doi: 10.1136/bmjopen-2023-081306

| **3. Will the research include any audio, video or photographic recordings?** |
|---|
| Yes. Participants who don't wish to be recorded could still take part. In this case researcher will take notes during the interviews. |
| **4. Biological sample handling** |
| No biological samples will be taken as part of the study. |
| **5. Please detail all expenses or gifts that will be offered to participants.**<br>Guidance is available in Best Practice Guidance: 05 Payments and incentives in research. |
| R~~No r~~emuneration will be given for participation in the study, in the form of £20 electronic gift vouchers for time taken to participate in interviews.~~.~~ |

| **SECTION F. ETHICAL CONSIDERATIONS**<br>For guidance on ethical issues, please see http://researchsupport.admin.ox.ac.uk/governance/ethics/resources<br>*(N.B. To complete, double click on the check boxes and select 'checked')* | | |
|---|---|---|
| **1. Will the research involve any participants considered vulnerable in the context of the research (e.g. children, elderly, prisoners)?**<br>**If yes,** please describe how they are defined as vulnerable and detail any CUREC Approved Procedures or guidance that will be applied to the research (for current documents and templates see https://researchsupport.admin.ox.ac.uk/governance/ethics/resources).<br>**If yes, and you cannot apply any Approved Procedure**, please cease completion of this form – a CUREC 2 application is required | Yes ☐ | No ☒ |
| | | |
| **2. Will unequal relationships exist between participants and those obtaining informed consent?**<br>**If yes,** describe the nature of the unequal relationship and how arising ethical issues will be addressed | Yes ☐ | No ☒ |
| | | |
| **3. Will the research involve questions and/or discussions of contentious and/or sensitive issues (e.g. information relating to ethnicity, political opinions, religious beliefs, physical/mental health or sexual life)?**<br>**If yes,** please justify why this is required and provide a copy of the questionnaire raising the issues that will be used in your research. | Yes ☒ | No ☐ |
| Healthcare professionals:<br>We know urine collection methods and procedure are not consistent throughout different clinical practices or clinicians. We would like to understand what drives the decision making. We know different countries and culture | | |



Armengol M, *et al. BMJ Open* 2024; 14:e081306. doi: 10.1136/bmjopen-2023-081306

have different acceptability to invasive or non-invasive procedures, so would like to test whether ethnicity plays a role.

Parents:

We would like our study to be diverse and include people from different social-economic backgrounds and ethnic backgrounds. In order to quantify the success rate of these goals, and report them, we will ask parents/carers to state their ethnicity and questions that will allow us to estimate their social-economic position.

Workshop:

In order to be able to report who took part in this study, we will ask demographic questions. Participants will have the chance of not answering if they don't feel comfortable doing so.

| | | |
|---|---|---|
| **4. Will the research involve deliberate deception of participants outside the scope of CUREC Approved Procedure 07?**<br>**If yes**, please cease completion of this form – a CUREC 2 application is required<br>**If no,** please either give details of the deception or state that no deception will be involved | Yes ☐ | No ☒ |
| | | |
| **5. Could the proposed research affect your own physical and/or psychological safety as a researcher?**<br>**If yes,** describe how you will manage this. | Yes ☐ | No ☒ |
| | | |

**6. How will you ensure the research is conducted according to the details given in this form?**

Members of the research team will meet weekly to discuss progress or issues and themes that are arising from the interviews in order to make decision about the continuity and direction of interviews. Moreover, these discussions will also support the data analysis. And if needed, another person will be review recordings to validate findings.

Any students involved in the study will form part of the research team and will present findings on weekly basis. Students will be supported in their analysis by senior researchers.

Procedures will be discussed during meetings. We have involved people with experience in qualitative studies, who will support the process. Additionally, senior members of the research team will be able to join interviews and analysis, in order to check procedures are followed.

If any participant has questions, they will be able to ask the research team directly. They will also be able to reach the principal investigator if they feel appropriate. We will acknowledge reception and will give an indication of how it will be dealt with. Any serious complaints will be forwarded to the Chair of the Research Ethics Committee at the University of Oxford (ethics@medsci.ox.ac.uk).

**7. Please give details of any other research-specific ethical and/or safety considerations**

None

**8. How do you propose to deal with / handle any incidental findings?**

Not applicable

| **9. Will any data or information from this study be provided to individual participants?** |
|---|
| No. The study aims to explore and validate challenges faced during urine collection. Therefore, there are no individual results to be provided. |

## SECTION G. OTHER CONSIDERATIONS

| | | |
|---|---|---|
| **1. Is any part of this research being conducted overseas?**<br>**If yes,** please give details below. Ensure you complete and submit a travel risk assessment form to your departmental safety officer. (This is necessary to ensure the travel/fieldwork is covered by the University's travel insurance – see http://www.admin.ox.ac.uk/finance/insurance/travel/) | Yes ☐ | No ☒ |
| | | |
| **2. Does your research raise issues relevant to the Counter-Terrorism and Security Act (the Prevent Duty), which seeks to prevent people from being drawn into terrorism?**<br>**If yes,** please say how you plan to address any related risks. Please see advice on this on our Best Practice Guidance Web Page. | Yes ☐ | No ☒ |
| | | |

## SECTION H. DATA MANAGEMENT AND HANDLING

All information provided by participants is considered **research data** for the purpose of this form. Any research data from which participants can be identified is known as **personal data**; any personal data which is sensitive is considered **special category data**.

Management of personal data, either directly or via a third party, must comply with the requirements of the General Data Protection Regulation (GDPR) and the Data Protection Act 2018, as set out in the University's Guidance on Data Protection and Research.

In answering the questions below, please also consider the points raised in the Data Protection Checklist and whether, for higher-risk data processing, a separate Data Protection Impact Assessment may also be required for the research. Advice on research data management and security is available from Research Data Oxford and your local IT department. Advice on data protection is available from the Information Compliance team.

**1. Please mark 'X' against the data you will collect for your research**

| | | | |
|---|---|---|---|
| Screening documents | ☐ | Audio recordings | ☒ |
| Consent records including participant name or other identifiers (e.g. written consent forms, audio-recorded consent, assent forms) | ☒ | Video recordings | ☐ |



Armengol M, *et al. BMJ Open* 2024; 14:e081306. doi: 10.1136/bmjopen-2023-081306

| | | | |
|---|---|---|---|
| Consent obtained anonymously (e.g. via online survey) | ☐ | Transcript of audio/video recordings | ☒ |
| Opt-out forms | ☐ | Photographs | ☐ |
| Contact details for the purpose of this research only | ☒ | Information about the health of the participant (including mental health) | ☐ |
| Contact details for future use (guidance) | ☒ | Physiological test results / measurements | ☐ |
| Field notes | ☒ | MRI scans | ☐ |
| Task results (e.g. questionnaires, diary completion) | ☐ | IP addresses (refer to Best Practice Guidance 09: Data collection, protection and management for guidance) | |
| Data already in the public domain.<br>Specify the source of the data: | ☐ | Other (please specify below) | ☐ |
| Previously collected (secondary) data | ☐ | | |

**2. How and where will each type of data be stored whilst the research is ongoing (until the end of all participant involvement)?**

List each type of data selected above, and explain how each will be physically transferred (including movement/sharing of audio files, paper records, electronic downloads etc.) from where it is collected to a suitable storage site (e.g. Nexus365 OneDrive for Business, SharePoint, University servers). State the storage location for each. Do not store unencrypted data in freely available cloud services or unprotected USB drives.

Refer to Best Practice Guidance on data collection, protection and management (BPG09).

Hard copies of data (consent forms and field notes) will be placed in a folder and transferred to an office at the Engineering Science Department as soon as possible. In the Engineering Science Department, consent forms will be stored in a locked filling cabinet. When, despite efforts, it is not practical to immediately store data in this location, a lockable filling cabinet in the researcher's home will be used and will be transferred to the Engineering Science Department as soon as possible.

All electronic data (typed field notes, audio recordings and personal data including contact details) will be transferred from the researcher's password protected device to a password-protected drive within the University network. The audio recording held by the researchers will then be deleted once the interview audio has been transcribed. These recordings will be securely transferred using the university file sharing system or Nexus OneDrive for business. Workshops work documents will be de-identified.

Identifying information from audio recordings will be removed from the transcripts. Transcription will be done by the researcher or approved University transcriber. De-identified transcripts may be uploaded into NVIVO 12 which will accessed by the research team on password-protected computers. This de-identified data may also be made available to other researchers.

The transcription will be stored as a Word file on encrypted computers within the University network.

**3. Will you use a unique participant number on research data instead of participant name?**

**If yes,** state whether or not you will retain a list of participant names against numbers (pseudonymisation via a linkage list).

**Where will the list be stored, and when will it be destroyed?**

Yes. Data identifiers will be labelled and stored securely and separately from the research data, on password protected computers. We will destroy participants information once research has been completed and published.

| |
|---|
| |
| **4. Who will have access to the research data?** |
| Researchers listed on this form will have access to the research data and other student/researchers working on the study. Access will be granted to the MS IDREC for the purposes of monitoring and/or audit of the research.<br>De-identified data may be made available to other researchers. |
| **5. If research data is to be shared with another organisation, how will it be transferred / disclosed securely?** |
| Any data will be shared securely using the university file sharing system (OxFile or Nexus365 OneDrive Business system) and returned the same way. The data will be stored in password-protected computers within their institutions. |
| **6. When and how will identifiable data be destroyed or deleted?**<br>N.B. If any identifiable data will be retained beyond the end of the study and/or indefinitely, please state what data this is, and the reasons for retention (e.g. contact details for future studies; photos used in publication). This must be clearly stated on participant information, and specific consent obtained. |
| All identifiable data will be removed from the transcript.<br>Unique participants number will be used. Audio recordings will be destroyed after publication.<br>Consent records and de-identified transcripts and worksheets will be kept 3 years after publication.<br>Personal data will be deleted from servers after the analysis and publication has been completed. |

| | | |
|---|---|---|
| **7. Please confirm that you will store other (non-identifiable) research data safely for at least 3 years after final publication or public release and adhere to any additional research funder policies.**<br>For more information about the University policies, please see the University's web pages on research data management.<br>**If 'Yes'**, please give details of who will store the data and on storage format, location and security.<br>**If 'No'**, please provide further details. | Yes ☒ | No ☐ |
| Non-identifiable data will be stored in a password-protected computer within the University network at the PI's department. | | |

| **SECTION I. PUBLICATION AND DISSEMINATION OF RESULTS** | |
|---|---|
| **1. How will you disseminate and feedback project outcomes at the end of the research?** | Study finding will be disseminated though publications in peer-reviewed journals, conferences and other articles such as for the Community Health MIC, Children and Young People MIC. Public journals or seminars. |

CUREC 1 Form Version 6.6 Version/Date: V.02~~1~~ 2~~1~~0.03~~10~~.2022~~1~~
Ethics number: R77332/RE001

| | |
|---|---|
| | We will aim to make these findings available to the scientific and wider community by following the Open Science guidelines. |

## DECLARATIONS AND SIGNATURES

**In providing signatures, the MS IDREC Secretariat will accept either:**

**Option 1:** Email confirmations sent from a University of Oxford email address. Separate emails should be sent by each of the relevant signatories as outlined below, indicating acceptance of their responsibilities.

**Option 2:** That the form be fully-signed with handwritten (wet-ink) signatures. Please scan these and the rest of the form pages to create a single PDF document and email to us.

## PRINCIPAL INVESTIGATOR (AND STUDENT IF APPLICABLE)

I/We, the researcher(s):

- Understand our responsibilities as outlined on this form and in the CUREC glossary and guidance
- Agree to start this research only after obtaining approval from the MS IDREC;
- Understand that the Principal Investigator must ensure that all researchers are suitably qualified and trained to conduct the research described, or are appropriately supervised until deemed qualified/trained;
- Agree to provide additional information as requested by the MS IDREC before approval is secured and as research progresses;
- Agree to maintain the confidentiality of all data collected from or about participants;
- Agree to notify the MS IDREC in writing immediately of any proposed change to the research, and await approval before proceeding with the proposed change;
- Agree to notify the MS IDREC if the Principal Investigator changes and supply the name of the successor;
- Will use the data collected only for the research for which approval has been given;
- Will grant access to data only to authorised persons; and
- Have made arrangements to ensure that personal data collected from participants will be held in compliance with the requirements of the GDPR and the Data Protection Act 2018.

| | |
|---|---|
| **Principal Investigator (Name)** | Jeroen Bergmann |
| **Principal Investigator (Signature)**<br>Pasted images of signatures cannot be accepted | |
| **Date** | 30/06/2021 |
| **Student (Name)** | |
| **Student (Signature)**<br>Pasted images of signatures cannot be accepted | |
| **Date** | |

CUREC 1 Form Version 6.6 Version/Date: V.02~~1~~ 21~~0~~.03~~10~~.2022~~1~~
Ethics number: R77332/RE001

## ACCEPTANCE BY HEAD OF DEPARTMENT/FACULTY OR DESIGNATED NOMINEE*

*Another senior member of the department may sign where the head of department is the Principal Investigator, or where the head of department has appointed a nominee. Example nominees include Deputy Head of Department, Director of Research, or Director of Graduate/Undergraduate Studies.

I have read the research proposal above. On the basis of the information available to me, I:

- consider the Principal Investigator/Supervisor and student researcher (if applicable) to be aware of their ethical responsibilities in regard to this research;
- am satisfied that the proposed design and scientific methodology are sound; the research has been subject to appropriate peer review and is likely to contribute to existing knowledge and/or to the education and training of the researcher(s) and that it is in the public interest.

| | |
|---|---|
| **Head of Department or designated nominee (Name)** | |
| **Head of Department or designated nominee (Signature)**<br>Wet-ink signature (not pasted electronic image)<br>*or*<br>The Head of Department/nominee can send an email (including PI name and study title) to ethics@medsci.ox.ac.uk confirming the above | |
| **Date** | |

CUREC 1 Form Version 6.6 Version/Date: V.02~~1~~ 21~~0~~.03~~10~~.2022~~1~~
Ethics number: R77332/RE001
