# Supplementary material for: Clinician and parent views on urine collection in precontinent children in the UK: a qualitative interview study
Source: BMJ Open. 2024 Apr 29;14(4):e081306. doi: 10.1136/bmjopen-2023-081306 (PMC11086548; doi:10.1136/bmjopen-2023-081306)
Supplement: Supplementary data [file bmjopen-2023-081306supp002.pdf]

Study Title: Exploring perspectives, priorities, and solutions of urine collection: A qualitative user centric study to understand practices and gain feedback on from healthcare professionals and parents/carers
Document Title: Topic Guide Healthcare Staff
Date and Version Number: 20-Oct-2021, V0.1
CUREC Reference Number: R77332/RE001

DEPARTMENT OF ENGINEERING SCIENCE
UNIVERSITY OF OXFORD

Interview questions

This is a semi-structured interview. The questions below provide a guide and are not fixed. The interviewee can talk freely during the interview and the conversation will guide which questions will be asked next. The interview has 3 stages: warm-up questions, main questions, and wrap-up questions.

Introduction

Reassure interviewee that this is an interview to understand practices and knowledge around urine collection in order to design new solutions including technology, rather than judging or evaluating practices of individual.

Healthcare Professionals:

Warm-up questions

Interviewees will be asked to complete these questions in writing. Demographic questions will have an option "prefer not to say". Interviewer will remind participants that they do not need to answer any question they don't wish to answer.

All Healthcare Professionals

- Tell us about yourself
  - which hospital do you work in?
  - what is your role?
  - how long you have been in the role?
- Demographic questions
  - Age
  - Gender
  - Ethnicity
- How did you hear about this study?

If Medical Practitioners/Nurses

- About position
  - Main location of practice
  - Country of medical or nursing degree if applicable
  - Years of working in as current specialty (ie. As GP / in ED / Admin)
  - Training level
  - Do you know how many children under 2 would have a urine collection a week?
  - Specialised in paediatrics
  - Additional postgraduate qualification in paediatrics or child health
- Number of full-time equivalent GPs at clinic/number of doctors in ED
- Number of full-time equivalent nurses
- Type of clinic (private, mixed, NHS, other)
- What is your role related to urine collection?

Main questions

If Medical Practitioners/Nurses

Deep dive into the urine collection process:

- In the hospital you work in / have worked in, is there a preference for a particular urine collection method?
- What is your experience carrying out urine collection in infants? Have you used all methods?
- How often do you perform urine collection in children? / (in a day/week how many urine samples are collected?)
- Think about a recent experience carrying out/ordering/supervising/assisting a urine collection in an infant
  - Age of patient
  - Physical and emotional condition of patient / parent
  - Where was the collection done? Home, hospital, GP practice?
  - Who ordered the urine collection?
  - Where did the collection take place?
  - How many people were involved in the procedure?
  - Who carried out the collection?
  - How long did it take?
  - Any feedback?
  - Follow-up of patient
- Tell me about how would you go about asking parents to obtain a urine sample from their child?
  - Instructions:
    - ~~Have you been taught how to give~~Do you provide urine collection instructions for parents?
      - If no: why not? (lack of time, lack of knowledge, don't think it's necessary)
      - If yes, what type of instructions do you provide?
        - Do you think this is normal practice with colleagues? (allude to the fact that most parents have mentioned not to get instructions, would they agree?.
          - Why do you think that is?
    - ~~Did you share this information with parents?~~
  - Procedure explanation
  - Why are you collecting the sample?
- Who decides to get a urine sample?
  - If you, when would you get a urine sample?. Is this process clear?
- What is your perception of non-invasive urine methods as a diagnostic procedure?
- What is your opinion about the urine collection devices available?
  - Invasive vs. non-invasive
  - What is the determining factor when choose a particular option?
- The NICE guidelines in UK suggest using the clean catch method. In your opinion is this the most common method used?
- Do you or your colleagues ever use voiding stimulating methods for clean catch (i.e. Quick-wee method, bladder-tapping method)?
  - If yes, what is the practice mostly used and your experience in terms of success rate, usefulness, difficulty
  - If not, why not?
- What do you think are the pros and cons of current urine collection devices/methods, if any?

Formatted

Formatted: Font: (Default) +Body (Calibri), 11 pt

Commented [MA1]: Here, trying to see, if they feel is an easy and valid method to diagnose an infection or if it's just a check list procedure.

Formatted: Font: (Default) +Body (Calibri), 11 pt

Formatted: List Paragraph, Bulleted + Level: 1 + Aligned at: 0.63 cm + Indent at: 1.64 cm

    - Re-visits
    - Misdiagnosis
    - Contamination
    - Usability
- Do you know of other novel urine collection devices?
- Having considered everything mentioned before,
    - what would the ideal process look like from beginning to end?
    - Where can/does the process go wrong?

If Bio-analyst

Deep dive into the urine collection processes:

- Tell me about the work you do in regard to urine analysis in infants?
- How many urine collections happen in a month?
- How reliable are urine samples of infants for diagnosis of infections?
    - Do you have information of the method used for urine collection?
        - If yes, what do you think about invasive and non-invasive methods of urine collection?
        - How does clean catch compare?
        - Do you think there are other practices that reduce contamination rates
- Is retesting a frequent occurrence?
    - How much do you think this cost?
    - In your opinion what drives the use of one method or another?
- Having considered everything mentioned before,
    - what does the ideal process look like from beginning to end?
    - Where can/does the process go wrong?

If admin staff

Deep dive into the urine collection processes:

- Tell me about your experiences with urine collection in infants?
- How many urine collections happen in a month?
- What is the cost?
    - What are the resources used for a urine collection in infants
        - Duration for a collection
        - Use of room
        - Staff
    - How does clean catch compare to other methods?
    - In your opinion what drives the use of one method or another?
- The NICE guidelines in UK suggest using the clean catch method. In your opinion is this the most common method used?
    - Have you ever heard of voiding stimulating methods for clean catch (i.e. Quick-wee method, bladder-lumbar method)?
        - If yes, what is the success rate?
- Are you aware of contamination rates in different urine collection methods?
    - How does this affect cost?
- Do you know of other novel urine collection devices?
- Having considered everything mentioned before,
    - what does the ideal process look like from beginning to end?
    - Where can/does the process go wrong?

Wrap-up questions

End with asking them if they have any questions, ensuring they are comfortable with everything that has been said, and whether they would be open to further interviews in the future:

- Are there any questions you think I should have asked? Is there anything you would like to add?
- Who/where else do you think it might be interesting for us to interview? - How come? (because of different dynamic, scale and scope of work etc).
- If I had any quick questions arise from reviewing my notes today, would it be possible to give you a quick call/email?
- Would you been open to further interviews from us in the future as we continue to develop this tool?

Do you have any questions from us, would you like to know more about our urine collection project?

Armengol M, *et al. BMJ Open* 2024; 14:e081306. doi: 10.1136/bmjopen-2023-081306
