# Supplementary material for: Clinician and parent views on urine collection in precontinent children in the UK: a qualitative interview study
Source: BMJ Open. 2024 Apr 29;14(4):e081306. doi: 10.1136/bmjopen-2023-081306 (PMC11086548; doi:10.1136/bmjopen-2023-081306)
Supplement: Supplementary data [file bmjopen-2023-081306supp003.pdf]

Study Title: Exploring perspectives, priorities, and solutions of urine collection: A qualitative user centric study to understand practices and gain feedback on from healthcare professionals and parents/carers
Document Title: Topic Guide Parents/Carers
Date and Version Number: 20-Oct-2021, V0.1
CUREC Reference Number: R77332/RE001

Interview questions

This is a semi-structured interview. The questions below provide a guide and are not fixed. The interviewee can talk freely during the interview and the conversation will guide which questions will be asked next. The interview has 3 stages: warm-up questions, main questions, and wrap-up questions.

Reminder of how we will use the data:

> We will record the interview. This will be transcribed, and all identifiable information will be erased from transcription and stored safely within the Engineering Department. Recordings will be erased.

Introduction

Reassure interviewee that this is an interview to understand what works and what doesn't work of current urine collection methods so we can design technology that helps parents and clinicians.

Warm-up questions

We will give demographic questions to interviewee for them to fill. The option of prefer not to say will be given. Interviewer will remind participant that they do not need to answer any question they prefer not to answer.

- Tell us about yourself;
  - Who do you care for?
  - How many children do you have?
  - Demographic questions
    - Age
    - Gender
    - Ethnicity (give options from a list)
    - Social demographics:
      - Post code
      - How many people live in the house?
      - Employment status (full time / part time / stay at home parent / unemployed)
      - Number of dependents
    - Education level
  - How did you hear about this study?

Main questions

Deep dive into the urine collection process:

- Tell me about your experiences with urine collection in infants and toddlers?
  - When did it last happen?
  - Where did the urine collection occur? i.e. hospital, home, GP

Armengol M, *et al. BMJ Open* 2024; 14:e081306. doi: 10.1136/bmjopen-2023-081306

- If you sought medical attention, what were the symptoms your child had that worried you?
- Tell me about how you were told to get the sample
  - How was the urine sample collected?
  - Were you given instructions? Do you remember them (the instructions you were given)? → Urine Collection Method
  - Who gave them to you?
  - Did someone explain why a urine sample was needed?
  - Was there any leaflet or other information that helped with the process?
    - If yes, do you remember any helpful information?
- How did you find the process of urine collection in your child?
  - What were the challenges?
  - What worked well?

- When did this last happen?
- Did this happen with only one or many of your children? Does it happen often?
- How often do you perform urine collection in children?
- Are you aware that there are different urine collection methods?
  - Have you previously used other methods?
  - If you used other ways of collecting urine, which method did you prefer?
    - Why?
  - Did you have any worries about some of the collection methods?

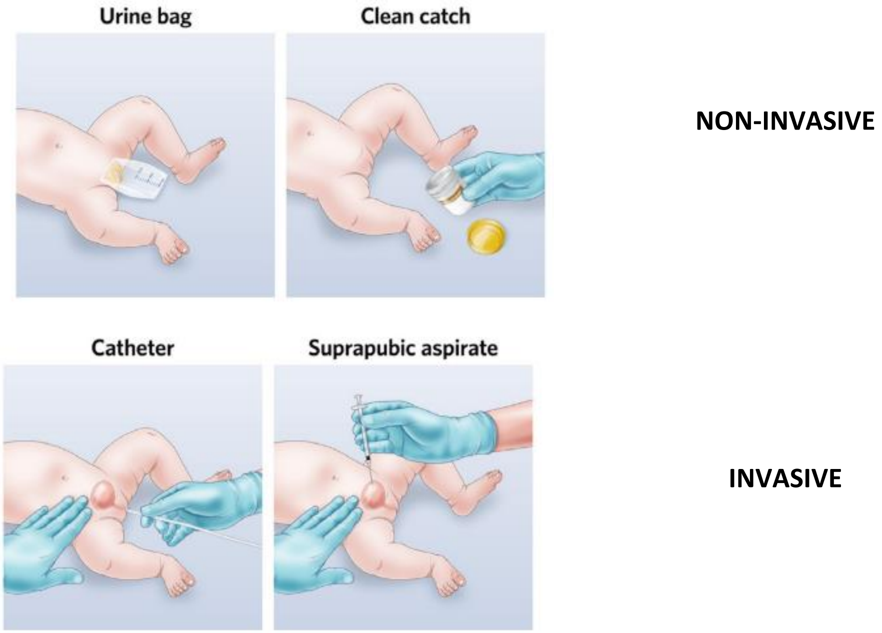

Figure 1. Images by Dr. Jonathan Kaufman and Bill Reid, Royal Children's Hospital. Kaufman et al, BMJ, 2017. 357: 16-19.

- What is your opinion about the urine collection devices available?

    - Invasive vs. non-invasive, i.e. a sample requiring a nurse/doctor placing a urinary catheter versus getting the child to sit on a potty
    - If there was more than one option, were you given a choice?
- The NICE guidelines in UK suggest using the clean catch method. In your opinion was this the method used for your child.
    - Have you ever heard of ways to speed up collecting urine samples (eg gently tapping the bladder)?
        - If yes, did it work? / How long did it take?
- Are you aware that some urine samples are unreliable because they end being contaminated with bacteria (bugs) commonly found on our skin?
- Do you know of other urine collection devices?
- In your opinion:
    - what would the ideal process look like from beginning to end?
    - Where can/does the process go wrong?

Wrap-up questions

End with asking them if they have any questions, ensuring they are comfortable with everything that has been said, and whether they would be open to further interviews in the future:

- Would you like to add anything that we didn't ask about?
- Do you know anyone else that has gone through urine collection in a non-toilet trained infant that might be interested in participating?
- If I had any quick questions arise from reviewing my notes today, would it be possible to give you a quick call/email?
- Would you been open to further interviews from us in the future as we continue to develop this tool?

Armengol M, *et al. BMJ Open* 2024; 14:e081306. doi: 10.1136/bmjopen-2023-081306
