# Supplementary material for: Clinician and parent views on urine collection in precontinent children in the UK: a qualitative interview study
Source: BMJ Open. 2024 Apr 29;14(4):e081306. doi: 10.1136/bmjopen-2023-081306 (PMC11086548; doi:10.1136/bmjopen-2023-081306)
Supplement: Author's manuscript [file bmjopen-2023-081306.draft_revisions.pdf]

BMJ Open is committed to open peer review. As part of this commitment we make the peer review history of every article we publish publicly available.

When an article is published we post the peer reviewers' comments and the authors' responses online. We also post the versions of the paper that were used during peer review. These are the versions that the peer review comments apply to.

The versions of the paper that follow are the versions that were submitted during the peer review process. They are not the versions of record or the final published versions. They should not be cited or distributed as the published version of this manuscript.

BMJ Open is an open access journal and the full, final, typeset and author-corrected version of record of the manuscript is available on our site with no access controls, subscription charges or pay-per-view fees (http://bmjopen.bmj.com).

If you have any questions on BMJ Open's open peer review process please email info.bmjopen@bmj.com

BMJ Open

Urine collection in young children: a qualitative study of health care professionals and parents

Journal:	*BMJ Open*
Manuscript ID	bmjopen-2023-081306
Article Type:	Original research
Date Submitted by the Author:	24-Oct-2023

Complete List of Authors:	Armengol, Monica; University of Oxford, Department of Engineering Science<br>Hayward, Gail; University of Oxford, Nuffield Department of Primary Care Health Sciences<br>Abbott, Molly; Guy's and St Thomas' Hospital NHS Trust, London<br>Bird, Chris; Birmingham Children's Hospital, Emergency Department<br>Bergmann, Jeroen; University of Oxford, Department of Engineering, Institute of Biomedical Engineering<br>Glogowska, Margaret; University of Oxford, Nuffield Department of Primary Care Health Sciences

Keywords:	PAEDIATRICS, Urinary tract infections < UROLOGY, QUALITATIVE RESEARCH

SCHOLARONE™
Manuscripts

I, the Submitting Author has the right to grant and does grant on behalf of all authors of the Work (as defined in the below author licence), an exclusive licence and/or a non-exclusive licence for contributions from authors who are: i) UK Crown employees; ii) where BMJ has agreed a CC-BY licence shall apply, and/or iii) in accordance with the terms applicable for US Federal Government officers or employees acting as part of their official duties; on a worldwide, perpetual, irrevocable, royalty-free basis to BMJ Publishing Group Ltd ("BMJ") its licensees and where the relevant Journal is co-owned by BMJ to the co-owners of the Journal, to publish the Work in this journal and any other BMJ products and to exploit all rights, as set out in our licence.

The Submitting Author accepts and understands that any supply made under these terms is made by BMJ to the Submitting Author unless you are acting as an employee on behalf of your employer or a postgraduate student of an affiliated institution which is paying any applicable article publishing charge ("APC") for Open Access articles. Where the Submitting Author wishes to make the Work available on an Open Access basis (and intends to pay the relevant APC), the terms of reuse of such Open Access shall be governed by a Creative Commons licence – details of these licences and which Creative Commons licence will apply to this Work are set out in our licence referred to above.

Other than as permitted in any relevant BMJ Author's Self Archiving Policies, I confirm this Work has not been accepted for publication elsewhere, is not being considered for publication elsewhere and does not duplicate material already published. I confirm all authors consent to publication of this Work and authorise the granting of this licence.

For peer review only

Urine collection in young children: a qualitative study of health care professionals and parents

Monica Armengol

Department of Engineering Science, University of Oxford

Gail Hayward

Nuffield Department of Primary Care Health Sciences, University of Oxford

Molly Abbott

Guy's and St Thomas' Hospital NHS Trust, London

Christopher Bird

Birmingham Women's and Children's NHS Foundation Trust and Nuffield Department of Primary Care Health Sciences, University of Oxford

Jeroen Bergmann

Department of Engineering Science, University of Oxford

Margaret Glogowska

Nuffield Department of Primary Care Health Sciences, University of Oxford

Corresponding Author:

Margaret Glogowska

Nuffield Department of Primary Care Health Sciences, University of Oxford

Radcliffe Observatory Quarter, Woodstock Road

Oxford OX2 6GG, UK

Tel: +44 (0)1865 617872

Email: Margaret.glogowska@phc.ox.ac.uk

For peer review only - http://bmjopen.bmj.com/site/about/guidelines.xhtml

Abstract

Objective

To explore the experiences of health care professionals (HCPs) and parents of urine collection methods, to identify barriers to successful sampling and what could improve the process.

Design

Qualitative research design, using individual semi-structured interviews with HCPs and parents. The interviews were audio-recorded, transcribed and thematically analysed.

Setting

UK-based HCPs from both primary and secondary care settings and parents with experience of urine collection in primary and/or secondary care settings.

Participants

HCPs who took part were involved in aiding, supervising or ordering urine samples. Parents who took part had experience of urine collection in at least one pre-continent child.

Results

13 HCPs and 19 parents were interviewed. Two of the participating HCPs were GPs, eight were nurses and three were doctors working in paediatric secondary care settings. Two of the parents had children with underlying conditions where frequent urine collection was required to rule out infections.

HCPs and parents reported that there were no straightforward methods of urine collection for pre-continent children. Each method – 'clean catch', urine bag and urine pad – had limitations and problems with usage. 'Clean catch', regarded as the gold standard by HCPs and with a lower risk of contamination, often proved difficult for parents to achieve. Other methods had elevated risk of contamination but were more acceptable to parents because they were less challenging. Many of the parents expressed the need for more information about urine collection in young children.

Conclusions

For peer review only - http://bmjopen.bmj.com/site/about/guidelines.xhtml

A new device is required to assist with urine collection in pre-continent children, to simplify and reduce the stress of the situation for those using it. Parents are key partners in the process of urine collection with young children. Meeting their expressed need for more information could be an important way to achieve better quality samples while waiting for a new device.

Strengths and limitations of this study

- We interviewed parents familiar with several of the different types of urine collection method and varied lived experience of urine collection.
- Most of the HCPs interviewed worked in environments where unwell children presented every day and where urine collection was a routine part of assessment.
- Interviewing both parents and HCPs gave us a dual perspective on the urine collection process.
- Our study includes relatively small samples of both parents and HCPs.

For peer review only

For peer review only - http://bmjopen.bmj.com/site/about/guidelines.xhtml

Background

Urinary tract infections (UTIs) are common bacterial infections in young children.[1] Diagnosis is challenging as symptoms typical of UTI in adults may be absent.[2] In one study, fewer than one third of children who presented to primary care with acute illness and met microbiological criteria for UTI had been clinically suspected of having a UTI.[3] Thus, there is a need for urine testing to exclude UTI in children where the cause of acute illness is unclear.[4]

In the UK, invasive testing methods to collect urine in pre-continent children such as catheterisation may be used in emergency settings where time is critical but suprapubic aspiration (SPA) under ultrasound guidance is rarely performed. There is debate around whether invasive methods should be used more,[5] although there is evidence that SPA is more painful in infants than catheterisation.[6] UK guidelines emphasise the use of non-invasive methods and recommend invasive methods only if non-invasive methods are not possible or practical.[7] However, non-invasive methods such as 'clean catch', urine bags and nappy pads to collect urine in pre-continent children are challenging to use and associated with high rates of contamination. [4,8,9,10] Contamination of samples presents a significant clinical problem in that it delays diagnosis, can lead to inappropriate prescription and overuse of antibiotics, requires repeat samples which adds healthcare costs and delays discharge, and can result in further unnecessary diagnostic tests such as renal ultrasound scans.[10]

A necessary step toward improving urine collection for pre-continent children is to understand current experiences and how things could be improved. Primary care Health Care Professionals' (HCPs') views of urine collection methods for pre-continent children have been explored using qualitative research methods.[4, 11] However, the more holistic picture, including the views of secondary care HCPs, often the first contact for unwell children, as well as those of parents, have not been explored.

For peer review only - http://bmjopen.bmj.com/site/about/guidelines.xhtml

Aims

The aims of this study were:

- to understand the experiences and perceptions of HCPs and parents of urine collection methods in primary and secondary care settings
- to identify barriers to successful urine sampling in pre-continent children and what could make the process more efficient

Methods

Design

We chose a qualitative research design – individual semi-structured interviewing – for our study. This design captures people's detailed accounts of their experiences and perceptions of phenomena. It allows researchers to explore their selected topics but gives participants the opportunity to raise issues significant to them. Ethical approval was granted by the Central University Research Ethics Committee, University of Oxford (R77332/RE002). We did not involve patients or the public in the design, conduct, reporting or dissemination stages of our research.

Recruitment and sampling

We invited HCPs and parents to participate. For HCPs we sought to include medical professionals at any stage of their training, general practitioners, and nurses. Inclusion criteria included aiding, supervising or ordering urine samples and being able to be interviewed in English. For parents, the inclusion criteria were having experience of urine collection in a pre-continent child within the two years prior to the interview and being able to be interviewed in English. This time period was sufficiently narrow to allow recall of events, but wide enough to ensure that enough parents could be recruited.

For peer review only

For peer review only - http://bmjopen.bmj.com/site/about/guidelines.xhtml

Recruitment took place through advertisements on social media groups of parents, charities, and paediatric organisations for HCPs; in newsletters around universities; on posters in nurseries; and on public news boards. The main recruitment material was a leaflet and poster which directed potential participants to the study website. Here they could find more information and register interest. They were then contacted by a female research team member (MAr or MAb) to schedule an interview. Participants received written information about the study and had the opportunity to ask questions. Participants gave written consent before interviewing took place.

For peer review only

Data collection

The interviews with HCPs and parents were conducted using separate topic guides around three main issues – the methods and process of urine collection experienced, difficulties encountered, and how urine collection could be made easier. The topic guides were employed flexibly, allowing the interviewers to follow up issues raised by participants, and modified as the study progressed. The interviews were conducted online using Microsoft Teams (by MAr and MAb) between December 2021 and July 2022, lasting for approximately 30 minutes. Methodological oversight was provided by an experienced female health services researcher (MG). Data collection ended when the researchers agreed that no new issues were raised in the interviews and there was sufficient understanding of the emerging categories.

Data analysis

Interviews were audio-recorded and transcribed. Transcripts were checked against the audio recordings, de-identified and uploaded to NVivo 12 (Lumivero, Denver, USA), a qualitative analysis software tool. To explore the range of experiences we encountered in the interviews, thematic analysis was used. Data driven analysis allowed us to identify the topics emerging from the HCP and parent data and group them into themes. An iterative approach to coding and grouping codes into

For peer review only - http://bmjopen.bmj.com/site/about/guidelines.xhtml

categories was taken.[12] Themes and sub-themes were identified, discussed and agreed with the research group to enhance trustworthiness.[13,14]

Findings

Sample

42 people expressed interest in participating through the website or email, but not all responded to contact from the research team. Altogether, the sample included 13 HCPs and 19 parents. Among the HCPs, two of the participants were GPs, eight were nurses working in paediatric secondary care settings and three were doctors working in paediatric secondary care settings. All HCPs who participated were practising in the UK. Working experience varied from 7 months to over 30 years. Among the parents, eight of the parents had one child and the other eleven had two. Two of the parents had children with underlying conditions where frequent urine collection was required to rule out infections. All but one of the parents interviewed were female. The parents were between 25 and 56 years old. All our participants identified as white, except three who identified as mixed race or Indian.

From the analysis of the interviews, two main themes emerged: acceptability and challenges of urine collection methods; and making the urine collection process more parent- and child-friendly.

Acceptability and challenges of urine collection methods

Invasive methods

HCPs and parents agreed that invasive urine collection methods, such as in and out catheterisation and suprapubic aspiration (SPA), were acceptable when a child was seriously unwell and a urine sample was urgently needed. For parents, SPA seemed the least acceptable method because they perceived it as painful for their child. Similarly, HCPs tended to regard SPA as the most invasive method and did not commonly perform it. One clinician regarded it as useful only if imaging was

For peer review only - http://bmjopen.bmj.com/site/about/guidelines.xhtml

used to guide collection of the sample. Both parents and HCPs were aware that invasive methods had additional risks, particularly introducing infection, limiting their acceptability.

<u>*Non-invasive methods*</u>

Clean catch

Among the non-invasive urine collection methods, 'clean catch' was preferred in the primary and secondary care settings where the HCPs we interviewed worked and the parents we interviewed sought care for their children. This usually involves direct catching of urine spontaneously produced by the child into a sterile container such as a pot or a bowl provided for that purpose. Several HCPs described this process as a *"nightmare"* with pre-continent children, acknowledging that it can be demanding for parents, messy, difficult to judge and time-consuming.

Parents found the 'clean catch' process challenging, if not impossible. They highlighted how emotional the process could be, also making it less acceptable. Very young unwell children often became upset at the process which further increased the stress for parents. The 'clean catch' method often required the child to be unclothed, at the least have their nappy removed, so the parent could see when urination occurred. Some parents and children found this aspect very upsetting. Even adapting the method to obtain a sample at home, such as holding a child over the bath, could be distressing. Knowing that they needed to obtain a sample to help pinpoint why their child is ill but having little control over when and if they can get one, increased the stress felt by parents. HCPs concurred that using the 'clean catch' method often meant that samples could be very delayed.

HCPs acknowledged that while the clean catch method was pursued to avoid contamination, the reality of attempting it diverged from the ideal. They were aware that sterile containers provided did not always remain sterile and that it was difficult, if not impossible, for parents to prevent the

For peer review only - http://bmjopen.bmj.com/site/about/guidelines.xhtml

container coming into contact with the child's skin. Some parents were aware of how contamination could occur but found it difficult to avoid. To reduce contamination from a 'clean catch' sample, several HCPs suggested that HCPs advise parents to wipe their child's genital area beforehand. Parents, however, had not received this instruction.

Urine bags

HCPs, particularly the GPs in our sample, and some parents, mentioned urine bags for urine collection but these were not used in all of the settings described. These are plastic bags placed around the child's genitalia using an adhesive which sticks to the skin. HCPs found the idea acceptable in principle as they caught urine without parents needing to follow their child around with a container. However, HCPs highlighted issues such as high contamination rates, leakage through loss of adhesion and difficulty with fitting the bag around the genitalia of both boys and girls, making them far less acceptable. Parents who hadn't used urine bags were optimistic about trying them. However, parents who had already used them found them far less acceptable in practice. They described similar issues to those of the HCPs – contamination, leakage, the bag losing adhesive and dropping off, and discomfort when the bag was pulled away from the child's skin in sensitive areas.

Urine pads

Normally used for children younger than 6 months, urine pads were mentioned less frequently than 'clean catch' and urine bags. These are absorbent pads positioned inside the child's nappy from which urine is collected via a syringe or squeezed out. HCPs regarded them as acceptable but acknowledged their problems, which included higher contamination rate than 'clean catch' and difficulty knowing whether any urine has been collected. Few parents had experience of urine pads for urine collection. A couple of parents mentioned using improvised 'pads' of sponge or cotton wool when desperate to obtain a sample at home, acknowledging that this was not ideal.

For peer review only - http://bmjopen.bmj.com/site/about/guidelines.xhtml

Table 1 contains a summary of this theme **Acceptability and challenges of urine collection methods**. Table 2 summarises the acceptability criteria for non-invasive urine collection methods discussed by parents and HCPs. For HCPs the acceptability of a non-invasive method of urine collection was largely determined by contamination risk. For parents, however, charged with obtaining the sample, acceptability was centred more around the difficulty of the method, the time taken and whether it caused their child distress.

Making the urine collection process more parent- and child-friendly

Communication between parents and HCPs

Parents often reported a lack of clear communication about urine collection from HCPs. Some parents felt they lacked an explanation of why a urine sample was required, while other parents felt it was only partially explained. Parents also suggested that HCPs assumed that they would know how to perform 'clean catch'. In one situation a parent was given a urine bag to use at home but needed to find instructions on the Internet. Parents often felt concern that they were obtaining samples correctly. Some were aware of contamination but lacked a step-by-step explanation of where it occurs and how to avoid it.

Some of the HCPs acknowledged spending little time giving parents full instructions. When asked if it was standard procedure for HCP to give parents instructions, one HCP thought there should be policies and written standards. Additionally, this HCP felt it was important to have guidelines around when to collect urine samples, so that parents were not burdened with it unless a sample was necessary for clinical decision-making.

Information for parents

For peer review only - http://bmjopen.bmj.com/site/about/guidelines.xhtml

HCPs and parents were asked about what could improve communication about urine collection. They agreed that formal instructions in a leaflet provided along with the urine collection container would be useful. HCPs were positive about giving parents written instructions as there was little time to pass on verbal instructions. Posters in clinical rooms where parents could read information were also viewed positively and one HCP suggested making a video parents could watch.

Parents and HCPs were asked about acceleration methods – actions which encourage or speed up urination. Mostly HCPs just recommended giving the child regular drinks but some were aware of other methods, such as turning taps on in the background, running a cool fan near the child, pouring warm water over the child's abdomen, and putting either warm or cool wet paper towels above their pubic area (Quick-Wee method).[15] Some HCPs found the tap method gave good results but was not practical, as it required a number of people to be involved. These methods were normally suggested only after time had elapsed without a sample collected. Few parents reported receiving any tips about speeding up urination. One parent accidentally found that as soon their child's nappy was removed and he stood on a cold floor urination happened. Parents, however, felt they would have appreciated suggestions from HCPs around acceleration methods even if they did not guarantee success.

For peer review only

Towards a new device design

Both parents and HCPs agreed on the need for a new device to simplify urine collection and reduce the stress of obtaining samples. Parents appreciated that no device could automatically reduce waiting time for urination but that it should enable clean, efficient collection when urination did occur. Parents were highly critical of the containers provided for urine collection, especially narrow pots designed for adults which they knew adults also struggle with. They felt that any new device using a container should be tailored according to sex and be wider to allow easier collection without touching the skin.

For peer review only - http://bmjopen.bmj.com/site/about/guidelines.xhtml

Parents and HCPs described the criteria they considered important in making a new device fit for purpose. A high priority for parents was a non-invasive device which didn't require constant monitoring. Parents and HCPs described how current methods could be adapted to make urine collection easier, such as a urine bag which doesn't need adhesive to stay in place. One HCP described how something like a nappy which would fit on the child would be helpful. Parents also favoured a wearable device for urine collection from a child resting or lying down. Several parents responded very negatively to removing their child's nappy and other clothing during the wait for a sample, especially where this caused distress. Thus, a device which could be used while the child remained clothed was considered more appropriate. Secondary care HCPs concurred that such a device would reduce the need for private spaces during the process.

HCPs perceived that simplicity of use was crucial in a new device so that staff could learn to use it quickly. Cost and any need for training in using a new device were also major considerations. Disposal was considered important by both parents and HCPs but both groups accepted that any device would probably need to be single use plastic as sterilisation was not likely to be possible.

Table 3 contains a summary of this theme **Making the urine collection process more parent- and child-friendly**.

Discussion

Invasive methods have a place in urine collection with pre-continent children but non-invasive methods were more commonly experienced in both the primary and secondary care settings, from which we drew our sample. We found that there were no straightforward methods of urine collection for babies and pre-continent children. HCPs and parents explained that each method described – 'clean catch', urine bag and urine pad – had limitations and problems with usage. 'Clean

For peer review only - http://bmjopen.bmj.com/site/about/guidelines.xhtml

catch', the urine collection method regarded as the gold standard by HCPs and with a lower risk of contamination, often proved difficult for parents to achieve. Other methods had elevated risk of contamination but were more acceptable to parents because they were less challenging and are still advocated as a last resort by UK national guidelines.[7]

While there is evidence that quicker sample collection can occur by the 'Quick-Wee' method,[15] acceleration methods did not appear to be routinely practised by the HCPs we interviewed, as they required a number of staff members to be involved,[16] and parents were typically not informed about them. Although GPs who had tried this method considered it impractical, such information could potentially improve the urine collection process for parents.[2] Parents often expressed the need for more information and support for collecting urine samples, as these may determine the management of their child's illness. This has a parallel with the information needs highlighted in a study conducted with adult women experiencing UTIs.[17]

Our findings demonstrate the need for improved urine collection methods, such as a device which could make the urine collection process less challenging for parents and less distressing for the children. Previous literature, detailing the strengths and shortcomings of various methods of urine collection, including all of those discussed in our study, also points towards the need for new methods. [2,4,11]

Strengths and limitations

Our study includes relatively small samples of both parents and HCPs who presented for interview. However, the sample includes a number of parents familiar with several of the different types of urine collection method and varied lived experience of urine collection. Similarly, the majority of the HCPs worked in environments where unwell children presented every day and where urine collection was a routine part of assessment.

For peer review only - http://bmjopen.bmj.com/site/about/guidelines.xhtml

We were able to achieve a dual perspective on urine collection. Comparing and contrasting the expectations and experiences of HCPs and parents helped us to identify where mismatches occurred and suggest why.

Conclusions

There is need for a new device to assist with urine collection in pre-continent children. Ideally, this would simplify and reduce the stress of the situation for those using it whilst reducing the contamination risk. Parents are key partners in the process of urine collection with young children. Meeting their need for more information and support in the process of collecting urine could be an important way to achieve better samples while waiting for a new device.

For peer review only

For peer review only - http://bmjopen.bmj.com/site/about/guidelines.xhtml

References

1 Campbell A, Hartling L, Louie-Poon S, Scott SD. Exploring the Experiences and Information Needs of Parents Caring for a Child With a Urinary Tract Infection: A Qualitative Study. *J Patient Exp*. 2021;8:1-8.

2 Boon H, Van den Bruel A, Verbakel J. GPs' perspectives on diagnosing childhood urinary tract infections: a qualitative study. *Br J Gen Pract.* 2022; DOI: https://doi.org/10.3399/BJGP.2021.0589

3 Butler CC, O'Brien K, Pickles T, et al. Childhood urinary tract infection in primary care: a prospective observational study of prevalence, diagnosis, treatment, and recovery. *Br J Gen Pract.* 2015; DOI: 10.3399/bjgp15X684361.

4 Boon HA, Lenaerts W, Van Aerde C, Verbakel JY. Outpatient urine collection methods for paediatric urinary tract infections: Systematic review of diagnostic accuracy studies. *Acta Paediatr.* 2021;110:3170– 3179. https://doi.org/10.1111/apa.16027

5 Cousin E, Ryckewaert A, de Jorna Lecouvey C, Arnaud AP. Urine collection methods used for non-toilet-trained children in pediatric emergency departments in France: A medical practice analysis. *Archives de Pédiatrie*. 2019;26:16–20.

6 Kozer E, Rosenbloom E, Goldman D. Pain in Infants Who Are Younger Than 2 Months During Suprapubic Aspiration and Transurethral Bladder Catheterization: A Randomized, Controlled Study. *Pediatrics*. 2006;118, 1:e51-e56.

7 NICE, Urinary tract infection in under 16s: diagnosis and management NICE guideline Published: 27 July 2022 www.nice.org.uk/guidance/ng224

8 Butler CC, Sterne JA, Lawton M, et al. Nappy pad urine samples for investigation and treatment of UTI in young children: the 'DUTY' prospective diagnostic cohort study. *Br J Gen Pract.* 2016;66(648):e516-524.

9 Tosif S, Kaufman J, Fitzpatrick P, et al. Clean catch urine collection: Time taken and diagnostic implication. A prospective observational study. *J Paediatr Child Health*. 2017;53:970–975.

For peer review only - http://bmjopen.bmj.com/site/about/guidelines.xhtml

10 O'Gorman J, Clarke J, Robinson M. 'Spending a penny, but keeping it clean' – reducing the rate of contaminated non-invasive samples in the paediatric department. *Arch Dis Child.* 2022;107(Suppl 2):A429–A430.

11 Kaufman J, Sanci L, Temple-Smith M. What's the catch? Urine sample collection from young pre-continent children: a qualitative study in primary care. *BJGP Open*. 2020; DOI: 10.3399/bjgpopen20X101060.

12 Silverman D. *Doing qualitative research: a practical handbook.*2018. London: Sage Publications: 2018.

13 O'Brien BC, Harris IB, Beckman TJ, Reed DA, Cook DA. Standards for reporting qualitative research: a synthesis of recommendations. Acad Med. 2014;89(9):1245-1251.

14 Lincoln YS, Guba EG. *Naturalistic inquiry.* 1985. Beverly Hills, CA: Sage Publications; 1985.

15 Kaufman J, Fitzpatrick P, Tosif S, et al. Faster clean catch urine collection (Quick-Wee method) from infants: randomised controlled trial. BMJ. 2017;357(j1341):1–8.

16 Herreros Fernández ML, González Merino N, Tagarro García A, et al. A new technique for fast and safe collection of urine in newborns. *Arch Dis Child.* 2013;Jan;98(1):27-9. doi: 10.1136/archdischild-2012-301872.

17 Glogowska M, Croxson C, Hayward G. Women's information needs around urine testing for Urinary Tract Infections: qualitative study. *Br J Genl Pract. 2022;*DOI: https://doi.org/10.3399/BJGP.2021.0564

For peer review only - http://bmjopen.bmj.com/site/about/guidelines.xhtml

Tables

Table 1 Summary of theme 'Acceptability and challenges of urine collection methods'

Subtheme	Topic and example quotations
Invasive methods	***Suprapubic aspiration and catheterisation***<br>But if you've got a very unwell baby and there's a time pressure to collect a sample, then essentially you either have to get a catheter sample, or if absolutely required, then very occasionally they would do a suprapubic aspiration [Interview 562, nurse, children's Emergency Department [ED]]<br><br>I would quite happily have allowed them to catheterise her…because she was very poorly…and if that could have meant that we just got some urine out and got an answer and could go home I would have taken that option. I don't think I would want that on the other occasions because it's not particularly nice for a child to have a catheter [Int 600, parent]<br><br>I haven't done a suprapubic aspirate for absolutely years and years…but generally, if you don't do them with imaging to help you then it's a bit pointless. [Int 767, consultant, children's ED]<br><br>***Risks around invasive methods***<br>If life or health could be permanently damaged by waiting an hour, then do what you need to do now. But obviously, it wouldn't have been ideal because…I'm sure using a catheter presumably carries some risk of infection and damage. [Int 869, parent]<br><br>If you were doing [an] invasive technique on everyone then you also put them at risk of introducing infection. They might not have one in the first place and then you can use a poor technique they're going to end up with one [Int 327, paediatric nurse, ED]
Non-invasive methods	***Challenges of the 'clean catch' method***<br>It can take ages and they [parents] missed the urine quite a bit. It ends up on the floor, on the bed, so it's not easy. [Int 984, advanced clinical practitioner, children's ED]<br><br>I mean, they're wildly, wildly unpredictable. And yeah, it is very tiring. It is very challenging because obviously, they don't wee all that frequently, so if you miss it, then it really delays their care. [Int 298, paediatric surgeon, secondary care]<br><br>She [was] really quite distressed trying to put [her] in a position, trying to catch the urine for them…She's getting upset screaming at me. I'm trying to catch it…she keeps kicking the bowl… and it's just an absolutely nightmare [Int 535, parent]<br><br>It wasn't warm…she had to be naked and she was poorly…I thought well, if I put the nappy on, I wouldn't know when she's weeing to be able to then act quickly to move her on top of the pot that was given. So I felt quite bad as a mum when she's crying and unwell and laying there really cold and you're having to try and just get on with it to try and get one [a sample]. [Int 266, parent]<br><br>I have a bit of a blurry memory of holding a baby over a bath at three in the morning. With the husband holding a pot trying to wait for a wee basically…you've done a feed and you're just waiting for pee…she was not happy to be held…held naked just kind of held a bit awkwardly for about 10, 15 minutes. She was not happy about that. She was crying quite a lot. [Int 088, parent]<br><br>There was a kind of time pressure…we need this checked off. And the sooner I can get the sample the sooner we can exclude that there's anything really dangerous going on. [Int 869, parent]

For peer review only - http://bmjopen.bmj.com/site/about/guidelines.xhtml

	Have the child out of a nappy and catch it when they wee, which obviously spends a long, long time…but then it means that everything is delayed. [Int 630, paediatric urology nurse, secondary care]<br><br>***Contamination and 'clean catch'***<br>But if you're sat there with the sterile pot for any period of time, it's gonna get touched by… the parent's finger. [Int 998, paediatric nurse, secondary care]<br><br>With the pot you've got to keep it very close to them, they've got to make sure that they're trying not to touch it [Int 980, advanced nurse practitioner, children's ED]<br>trying to not allow, the genitalia to touch the funnel, I don't think it's achievable. [Int 562, senior nurse, children's ED]<br><br>So I have had the words "get a sterile sample"… and I just sort of think well that's really hard, especially when I was having to do it in such a way when…she was lying down and you were trying to hold a little collection tube or something under. It was touching, you try not to touch the skin or anything, but you kind of have to, and then…I knew it wasn't clean, like as clean as they would want it, because it just it was just impossible. [Int 535, parent]<br><br>We've got some wipes, you want to clean the area before we wait particularly, you know, mainly the girls really the girls are worse. [Int 016, paediatric registrar, secondary care]<br>Nobody tells you make sure their bum is really clean or gives you anything to clean their bum with before they sit in this bowl [Int 307, parent]<br><br>***Experiences around urine bags***<br>The bags were much easier. You were guaranteed to catch, to capture it, you could put the nappy back on. They can run around, you can put them back in the waiting room. [Int 984, advanced clinical practitioner, children's ED]<br><br>There's the little bags that you stick that go inside the nappy but I haven't used them once in my job now…but from what I've been told by colleagues they notoriously don't stick. The urine leaks out of it, also they're not necessarily as sterile. [Int 543, nurse, children's ED]<br><br>Especially that bag, I would have given it a go. I suppose all children are different but…I think that bag one probably would work better than just looking out for wee and when she's going and having it [a pad under her] on the floor ready. [Int 266, parent]<br><br>It's not simple either [using the bag], because, it does stick on her and I think when you kind of peel it off, it leaves like a residue on her, so I think even like peeling it off at the end is a little bit probably a little bit painful, kind of like a bandaid, on those sensitive parts coming off probably does not feel good for her [Int 698, parent]<br><br>***Experiences around urine pads***<br>We can't do it that way because it's not a clean sample. So then I'll explain to them like if it does come up as positive for infection, and it's not a clean sample, we then have to repeat the sample anyway. [Int 327, paediatric nurse, ED]<br><br>Pads are not quite so easy because you can't tell that they've been used without looking at them… and in the past it just used to be just like I have to touch the nappy to see whether it was wet or not [Int 630, paediatric urology nurse, secondary care]<br><br>I used a new sponge and I cut it in half and I just put it between her legs and put a pair of pants on rather than a nappy…then when I felt she'd weed on me, I quickly grabbed the sponge and just wrung it out…I know that was probably classed as contaminated in some way [Int 266, parent]

For peer review only - http://bmjopen.bmj.com/site/about/guidelines.xhtml

Table 2 Acceptability criteria for urine collection using non-invasive methods in young children for HCPs and parents

	HCPs	Parents
**Preventing contamination in urine samples**	Highly important in determining acceptability of the method.<br><br>Uncontaminated samples necessary to guide diagnosis and treatment. 'Clean catch' recommended for this reason.	Not always aware of the importance of uncontaminated samples so less important in determining acceptability of the method.
**Ease of collection**	HCPs not involved in collection process so do not always experience the difficulties encountered by parents.<br><br>HCPs aware that 'clean catch' is challenging but still consider it the best option.	Highly important in determining acceptability of the method.<br><br>'Clean catch' perceived as very difficult and thus other methods, seen as less difficult but where contamination rates are higher, may be preferred.
**Amount of time taken for urine collection**	Important for HCPs as delay in urine collection can delay diagnosis/treatment.<br><br>HCPs aware that 'clean catch' can take a long time but still consider it the best option.	Very important as urine collection process can be lengthy.<br><br>'Clean catch' can require vigilance over long periods of time from parents. This can be particularly challenging if urine collection taking place in hospital.
**Urine collection causing distress to child**	HCPs not involved in urine collection process so do not always experience the distress caused to the child first hand.	Very important as child's distress, especially if undressing is required, during urine collection process adds to parental stress.

For peer review only

For peer review only - http://bmjopen.bmj.com/site/about/guidelines.xhtml

Table 3 Summary of theme 'Making the urine collection process more parent- and child-friendly

Subtheme	Topic and example quotations
Communication between parents and HCPs	***Explanation of the need for a urine sample***<br>It seemed quite random and unnecessary to then do it [get a urine sample]…the link wasn't really explained to me what the need for the sample was…so I tried, but yeah, it wasn't really clear as to what we were looking for in the sense of what she'd seen and why she felt she needed one [a sample]. [Int 266, parent]<br><br>They said that it [urine sample] would be worth having…because all of the other ones [tests] came back alright. It was one of those extra things to check. [Int 519, parent]<br><br>***Information about the 'clean catch' and other methods***<br>They just gave me the containers and said to send it back when I had collected it. [Int 881, parent]<br><br>In your cubicle [in hospital], they didn't give any specific instructions on how to collect. So they gave me the pot and said just make him pee here. And that was pretty much all the instructions that I received. [Int 479, parent]<br><br>No, I wasn't actually [told how to use the bag at home]. I think I looked it up online just to check. [Int 009, parent]<br><br>I've been told you know, be careful that doesn't get contaminated [urine container] but how particularly I do that I don't know…I've ended up following them around with Tupperware pots that I wiped with like sterilising wipes first…I mean I doubt it's that clean…I take their nappy off… but I didn't sort of do anything in terms of cleaning around their bottoms or anything. [Int 600, parent]<br><br>***Giving parents instructions***<br>I'm probably not very good at it actually. I probably say we're gonna need a urine and give them the collection pot and show them sort of how it works and just sort of say…it's a case of sitting there and waiting until they pee…I think I mean, you could blame time, but I think that's an excuse, not a reason…it's something we're doing all the time and they're not. [Int 998, paediatric nurse, secondary care]<br><br>***The need for policies around urine collection to help parents***<br>The amount of times people are set up for urine collections, there is a policy within the trust about urine collection, but I think it should be more specific about methods…..(When I) went up to the acute admissions unit, the nursing staff up there had never really seen it [a urine collection in pre-continent children] done, even their senior ones. So that information [about collection methods], I think sometimes gets lost unless you put it within a policy…I didn't think that they're [junior doctors] given enough education, and I certainly there's not a standard for it. [Interview 562, senior nurse, children's ED and acute ward]<br><br>I don't think some of them, specially when the junior doctors come through, they don't necessarily recognize how difficult it is for families to collect urines to make sure that they do definitely need them rather than arbitrarily saying, oh, I need a urine, yes, but that might take 2 hours and do you really need it for your clinical decision making? Is there an option to let the family go home and do it, you know? [Interview 562, senior nurse, children's ED and acute ward]
Information for parents	***Providing better information about urine collection for parents***<br>I think a leaflet is fine. Especially if there's like a diagram or something. Yeah, if a doctor wants to take the time to explain that as well, that's helpful…and then if you have questions,

For peer review only - http://bmjopen.bmj.com/site/about/guidelines.xhtml

	obviously, you can ask them…What happens a lot is that people assume…that you've been told stuff already. [Int 507, parent]<br><br>It would be something that had fairly clear instructions for the parent, and that were sort of easy to convey fairly quickly too. Because, obviously, there's always the time pressures in general practice. [Int 201, GP]<br><br>We were actually thinking about doing a bit of a quality improvement project in our department…making some videos on common procedures…that the parents could watch. So that was a way to potentially think about making it a bit more consistent as to what is explained. [Int 980, advanced nurse practitioner, children's ED]<br><br>***Information about 'acceleration' methods***<br>So the first and most definite, if the kid doesn't drink then nothing is going to come out. [Int 285, staff nurse, secondary care]<br><br>But the problem is with the tap method, why it's not used as much as because it takes three people to do it. [Int 562, senior nurse, children's ED and acute ward]<br><br>I think putting his feet on the floor because it was cold made him wee. I tried blowing as well 'cause that's, you know, as soon as you take a boys nappy off they usually wee… [Int 451, parent]
Towards a new device design	***Desired characteristics of a new device***<br>I think the other thing that really struck me was the shape of the sample pot. Seemed really non ideal for I mean, if it had been more like a funnel or something like that, that [could] have managed to catch a stream of urine in a really narrow pot. [Int 869, parent]<br><br>Something like the urine bag, but that is easier to attach and to detach, because he has been stressed when we have tried taken it off. [Int 881, parent]<br><br>I think ideally, there would be something that you can just take the baby out of their nappy and put on another nappy and wait for them to wee and then have got a sterile sample…So I think we're removing the kind of human element of having to watch and wait and time to catch it, that would be the best thing. [Int 298, paediatric surgeon, secondary care]<br><br>I think maybe that whatever item that might be designed, it needs to sort of be easy to use that any and everyone can like learn how to use it fairly quickly because obviously there are a lot of GPs in the practice. [Int 570, GP]<br><br>Our trust would never pay I don't think for expensive kit. If it can be done cheaply, then that's the way to do it. [Int 767, consultant, children's ED]<br><br>It's just something simple, something that the parents or the staff can easily use. And then I suppose easily disposed of as well. [Int 630, paediatric urology nurse, secondary care]

For peer review only - http://bmjopen.bmj.com/site/about/guidelines.xhtml

Declarations

Ethics approval and consent to participate

Ethical approval was granted by the Central University Research Ethics Committee, University of Oxford (R77332/RE002). Participants received written information about the study and had the opportunity to ask questions. Participants gave written consent before interviewing took place.

Availability of data and materials

The datasets generated and analysed during the current study are not publicly available because participant consent to share the original data outside the research team was not sought but reasonable requests for further information which does not compromise participant anonymity and confidentiality may be obtained from the corresponding author.

Competing interests

The authors declare that they have no competing interests.

Funding

This work was supported by the Engineering and Physical Sciences Research Council (EPSRC) Impact Acceleration Fund at the University of Oxford and the National Institute for Health Research (NIHR) Community Healthcare MedTech and In Vitro Diagnostics Co-operative.

Authors' contributions

MAr, JB, GH and MG were involved in the design of the study. MAr and MAb recruited participants, collected all the data and led analysis of the data, supervised by MG and GH. All authors were involved in interpretation of the analysed data and contributed to the final manuscript. All authors read and approved the final manuscript.

Word count

3019 words

For peer review only - http://bmjopen.bmj.com/site/about/guidelines.xhtml

SECTION A. RESEARCH DETAILS	
**1. Full title of research**	Exploring perspectives, priorities, and solutions of urine collection: A qualitative user centric study to understand practices and gain feedback from healthcare professionals and parents/carers
**2. Short title of research**	Urine collection qualitative studies
**3. Principal Investigator (PI) / Student Supervisor**	Dr. Jeroen Bergmann
**4. PI's training in research ethics and/or GCP**	"Children and Clinical Research" from the Global Health Training Centre, Course completed on 31/08/2020 JB<br>"ICH Good Clinical Practice (GCP) E6", from the Global Health Training Centre, Course completed on 31/08/2020 JB<br>"Research Integrity", University of Oxford, Course completed on 31/08/2020 JB<br>"Research Integrity in Engineering and Technology", University of Oxford, Course completed on 26/02/2015 JB<br>"Introduction to Research Ethics" from the Global Health Training Centre, Course completed on 19/01/2015 JB<br>"Human Research", Basic Course, Collaborative Institutional Training Initiative, MIT, Completed on 12/11/2013 JB
**5. Student name and degree programme (if applicable)**	
**6. Department/Institute name**	Department of Engineering Science
**7. University email address**	jeroen.bergmann@eng.ox.ac.uk
**8. University telephone number**	
**9. Funding Source**	EPSRC IAA / NIHR BRC
**10. State any conflicts of interest and explain how these will be addressed**	None

For peer review only - http://bmjopen.bmj.com/site/about/guidelines.xhtml

SECTION B. RESEARCHERS

1. **Researcher title and name**	Dr. Monica Armengol
2. **Department / Institute name**	Department of Engineering Science
3. **Role in research**	Researcher will obtain informed consent and conduct research activities with participant
4. **Training in research ethics and/or research integrity**	"Children and Clinical Research" from the Global Health Training Centre, Course completed on 23/06/2021 MA<br>"Research Integrity", University of Oxford, Course completed on 24/06/2021 MA
5. **Researcher title and name**	Dr. Christopher Bird
6. **Department / Institute name**	MIC Oxford Health NHS Foundation Trust
7. **Role in research**	Design and supervision of project, liaise with medical professionals
8. **Training in research ethics and/or research integrity**	GCP up to date July/2021 (Health Research Authority, the governing body for NHS Ethics in the UK)
9. **Researcher title and name**	Dr. Gail Hayward
10. **Department / Institute name**	Primary Care Health Science
11. **Role in research**	Design and supervision of project
12. **Training in research ethics and/or research integrity**	GCP, 2019 (Health Research Authority, the governing body for NHS Ethics in the UK)
13. **Researcher title and name**	Dr. Philip Turner
14. **Department / Institute name**	Primary Health Care Science
15. **Role in research**	Design and supervision of project
16. **Training in research ethics and/or research integrity**	"Research Integrity", University of Oxford, Course completed on 21/09/2021
17. **Researcher title and name**	Nathaniel Mills
18. **Department / Institute name**	NIHR Children & Young People MedTech Cooperative

For peer review only - http://bmjopen.bmj.com/site/about/guidelines.xhtml

**19. Role in research**	Support and supervision. Liaise to medical professionals
**20. Training in research ethics and/or research integrity**	NIHR Good Clinical Practice course
**21. Researcher title and name**	Dr. Paul Dimitri
**22. Department / Institute name**	NIHR Children & Young People MedTech Cooperative
**23. Role in research**	Support and supervision. Liaise to medical professionals
**24. Training in research ethics and/or research integrity**	GCP (Health Research Authority, the governing body for NHS Ethics in the UK)<br>GCP Paediatrics Module (Health Research Authority, the governing body for NHS Ethics in the UK)
**25. Researcher title and name**	Gemma Wheler
**26. Department / Institute name**	NIHR Children & Young People MedTech Cooperative
**27. Role in research**	Design of study and analysis support
**28. Training in research ethics and/or research integrity**	NIHR Good Clinical Practice course
**29. Researcher title and name**	Molly Abbott
**30. Department / Institute name**	Oxford Health NHS Foundation Trust and University of Oxford
**31. Role in research**	Recruitment of Interviewees and performing interviews
**32. Training in research ethics and/or research integrity**	Oxford GCP training course for clinical researchers 28 March 2021

**SECTION C. BASIC INFORMATION**	
**1. Provide a brief lay summary of the aims and objectives of the research. This should cover the questions it will answer and any potential benefits.**<br><br>**(Maximum 300 words)**	Urine is used to diagnose and monitor different diseases including diabetes and urine tract infections (UTI). Paediatric UTI accounts for 14% of the emergency department visits. Despite the high occurrence, collection of urine samples in non-toilet trained infants is challenging, lengthy and up to 30% of urine samples are contaminated with bacteria from the back passage or skin, making results meaningless.<br><br>Re-testing urine samples due to contamination causes delays, increases visits to the GP and emergency services. Additionally, it is associated with misdiagnosis, which can cause bacterial resistance if children are prescribed an antibiotic due to erroneous diagnosis.<br><br>This study aims to understand the current practices of urine collection in non-toilet trained infants and the challenges faced when carrying out, ordering or waiting for a collection.

For peer review only - http://bmjopen.bmj.com/site/about/guidelines.xhtml

	These findings will guide the focus areas for the development of a new device for paediatric urine collection, requirements, and specifications of the device.<br><br>We believe that a user-centric approach in the development of medical devices, produces device of higher clinical value. Participants will provide input in two parts: (1) interviews will aim to explore practices, costs, experiences, and perceptions of available diagnostic technology used for urine collection in infants in clinical settings. Additionally, we will explore unmet needs with regards to urine collection technologies and the potential impact of new diagnostic methods, tools or technology.<br>(2) a co-design workshop will allow participants to have input into possible solutions to the urine collection issues reported during the interviews.
**2. List all places where research will be conducted (including any other countries and online)**	Most interviews and workshop will be conducted online, via teams/zoom. Interviews will be conducted out of NHS premises.
**3. Anticipated research start date**	As soon as ethics is approved. Oct-Nov 2021
**4. Anticipated research end date**<br><br>(n.b. A maximum of 5 years approval can be granted)	3 years after approval
**5. Please list any CUREC Approved Procedure(s) you will follow**	
**6. Please list any CUREC Best Practice Guidance used to develop your research**	BPG 01 Researcher Safety/ BPG 03 Elite and expert interviewing/ BPG 09 Data collection, protection and management/ BPG 10 Conducting research interviews
**7. Name of departmental / peer reviewer (if applicable)**	Engineering Science / Sarah Hoeksma

**SECTION D. PARTICIPANTS**	
**1. Age range of participants**	Between 18 and 70 years old
**2. Are research participants classed as people whose ability to give free and informed consent is in question?**	Yes
**3. Anticipated number of participants**	Around 30 healthcare professionals and 20-30 parents

For peer review only - http://bmjopen.bmj.com/site/about/guidelines.xhtml

**4. How was the number of participants decided?**	We anticipate conducting between 20-30 interviews to healthcare professionals and 20-30 interviews to parents/carers. However, recruitment will continue until data saturation is reached or sufficient exploratory power for the findings is achieved. Further interviews might be required to fully explore the topics proposed. The research team will decide on concluding interviews.<br><br>For the workshop, we anticipate around 30 people taking part. This will include parents and healthcare professionals that took part in the interviews, in addition to other parents, health care professionals, engineers, designers and general public that are interested in taking part.
**5. Inclusion criteria**	Healthcare Professionals<br><br>Consenting adults with experience working with urine collection whether direct or indirect (perform, assist, manage, purchase, order or interpret) within a clinical setting. These include medical professionals, nurses, administrative staff and bioanalyst.<br><br>Parents/Carers:<br><br>Consenting adults with experience aiding a pre-continent child collect a urine sample in the last 2 years.<br><br>The adults taking part in the interviews, both parents/carers and healthcare professionals, must be able to take part on the interview conducted in English and consent to the interview being recorded. Participants must be over the age of 18 years and below 70 years old.<br><br>Co-design workshop<br><br>Consenting adults over the age of 18 years and below 70 years old with an interest in urine collection in infants, including those that took part in the interviews. Additionally, engineers, designers, industry experts and academics will be invited.
**6. Exclusion criteria**	The participant may not enter the study if they are unable to take part in an interview in English or written consent cannot be provided.
**7. Please mark 'X' against all planned recruitment methods**<br><br>Provide copies of all recruitment material for review	Poster advert ☒<br>Flyer ☒<br>Email circulation ☒<br>In-person approach ☒<br>Website ☒<br>Social media (e.g. twitter, Facebook) ☒<br>Snowball sampling (recruiting through contacts of existing participants) ☒<br>Newspapers ☐<br>Research recruitment sites (e.g. Prolific Academic, Amazon Turk) ☐<br>Existing departmental contacts or volunteer database ☒<br>Other (please specify) ☐
**8. How will potential participants be identified and approached?**	Information about the study and poster will be shared in social media (including Facebook groups [with permission of the group moderator] i.e. Community and parents groups, for example [Oxford] Community, [Oxford] Parents & Careers, paediatric organisations, charities and research groups), twitter (personal

For peer review only - http://bmjopen.bmj.com/site/about/guidelines.xhtml

	accounts, research group account and organisations we are collaborating with – NIHR Children and Young People (CYP) Medical Technology and In-vitro Co-operative (MIC)/ NIHR Community Health (CH) MIC), blogs.<br><br>Newsletters and mailing lists could be used to share information and poster. Poster will include detail of £20 remuneration in the form of electronic gift vouchers will be offered for time taken to participate in interview.<br><br>These will include the University Department's mailing list charities and baby groups, paediatric medical organisations and, partners from the NIHR MICs will share information in their website and newsletter. The emails will be sent on behalf of the researchers conducting this study. We will also publish information of the study in our website and rely on word of mouth of participants.<br><br>Participants can express their interest in taking part of the study by contacting the researcher by email or phone. We will also include a form on the website for people to express their interest. Any potential participant expressing interest in taking part in the study will be contacted by email or telephone. Potential participants will be directed to our website where they can download the participant information sheet and consent form. Participants wishing to take part will be asked to return the consent form and participant information sheet signed. Participants wishing to take part, will be invited (by email or phone) to take part in the co-design workshop. Information and invite in the co-design workshop will use the same medium as the interviews did for distribution.<br><br>Participants will be given options regarding the timing, format, and venue (if face-to-face) for the interview will be given. The participant will have the opportunity to ask any questions either by email or telephone. They may choose to decline any further contact; in which case they will be reassured that this will in no way impact upon their current or future working.<br><br>The workshop will have a set date, participants will be asked to register their attendance in advance.
**9. Will informed consent be obtained from the research participants or their parents/ guardians?** If not, please explain why not.	Verbal consent will be taken at the start of interviews and co-design workshop. Before the interview/workshop starts, we will explain the study to the participant and go through the consent form.<br><br>Written informed consent will be obtained by means of participant dated signature and dated signature of the person who presented and obtained the Informed Consent.<br><br>Written consent will be sought participants will be given the option to fill the consent themselves and emailing it to us, they can also complete it through a GDPR compliant platform (https://www.smartsurvey.co.uk/) or researcher can fill them on their behalf and email a password-protected copy and retaining a copy for the records. For interviews being done by phone, form will be sent by email, or if not possible, oral consent will be sought.<br><br>Participant will be able to ask any questions or withdraw at any point without any explanation.
**10. For each activity or group of participants, explain how informed consent will be obtained from the participants themselves and/or their**	Verbal consent will be taken at the start of the interview and workshop. Study will be explained and the researcher will go through the consent form with the participant.<br><br>Written consent will be sought prior to interviews and co-design workshop are taking place. As interview/workshop will be conducted remotely (either online or

For peer review only - http://bmjopen.bmj.com/site/about/guidelines.xhtml

**parents/guardians, if applicable. How will their consent be recorded?**	by phone) participants will be given a few options to fill the consent form; the option to fill the consent themselves and emailing it to us, they can fill it online through a GDPR compliant software (https://www.smartsurvey.co.uk/) or researcher can fill them on their behalf and email a password-protected copy and retaining a copy for the records. A copy of the consent form will be made and stored safely in password protected file.<br><br>Participants will be given the opportunity to ask questions or withdraw from study. If participants are happy to proceed, interviews will be arranged online or by telephone at their convenience. A date will be set for the workshop, participants can register their interest to participants through the website. We will be available to reply to any questions or concerns that the participant may have at any point during this process.

SECTION E. RESEARCH METHODOLOGY

1. Please mark 'X' against the methods that will be used in your research

Ensure you address each method you will use in your informed consent documents and on this form

Use of casual or local workers (e.g. interpreters)	☐	Audio recording of participant	☒
Interview (refer to guidance in BPG 10: Conducting research interviews)	☒	Video recording of participant	☐
Focus group	☐	Photography of participant	☐
Participant completes questionnaire in hard copy	☐	Physiological recording from participant	☐
Participant completes online questionnaire or other online task (refer to guidance in BPG 06: Internet-mediated research)	☐	Taking a sample of blood or other bodily fluid from a participant	☐
Use of social media to recruit or interact with participants (refer to guidance in BPG 06: Internet-mediated research)	☒	Participant observation	☐
Analysis of existing records	☐	Covert observation	☐
Participant performs verbal or aural task	☒	Systematic observation	☐
Participant performs paper and pencil task	☒	Observation of specific organisational practices	☐
Participant performs computer based task	☒	Other (please specify below)	☐
Measurement/recording of motor behaviour	☐		

2. Provide a lay description of the research design and methods. In particular, describe clearly what participants in the research will be asked to do.

Interviews:

Before interview starting, the information sheet will be reviewed, and any questions addressed.

- Participants will be asked to provide their informed consent to participation prior to data collection. Participants will be explained to that their participation is voluntary and they can end or withdraw from study at any point without any explanation.

For peer review only - http://bmjopen.bmj.com/site/about/guidelines.xhtml

- A date for the interview to take place will be scheduled. Participants will have a choice of date, time and place of interview (online or telephone).
- Sessions will last between 40-60 min. Each participant will have one session. Consent will be asked in case follow-up is required in any of the questions.
- A qualitative methodology will be used given that it is appropriate to explore people's knowledge, choices, and decision. Data collection and analysis will be guided by the principles of grounded theory which allows adjustments to questions and sampling.
- Semi-structured questionnaires with open-ended questions will be used for the interviews. A topic guide will be used to conduct the interviews. This will allow flexibility in the order and wording of the questions. Additionally, questions will be amended based on on-going interviews, to explore emerging ideas and themes and to include new participants as participants for interview (theoretical sampling).

The topic guide will be divided into three parts, a warmup section that will include personal information and questions to get to know the person being interviewed and their relationship to urine collection. Then the main questions section, which will determine experiences, stories and opinions about urine collection methods. Finally, the wrap-up questions, to give the opportunity to ask any questions to us.

The open-ended questions will be used to probe the different areas of urine collection we are interested in. The interviews will explore the procedure, i.e., who uses it, device used, procedure (who collects the urine, how long it takes, final sample), post-collection feedback and process, patient follow-up.

- Interviews will be audio recorded and transcribed. If only audio is not possible (during online meeting),participant will be asked to turn off their video off during the interview..

Co-design Workshop

- Participants will be invited to take part in the workshop. They can express their interest by filling a registration form on the website.
- Participant will be able to download the consent and participant information form from the website. And will be able to contact the researchers with questions using the contact information provided in the participant information sheet.
- Participants will be asked to provide their informed consent to participation before the workshop starts. Participants will be explained to that their participation is voluntary and they can end or withdraw from study at any point without any explanation. They will also have the opportunity to ask questions.
- Participants will be divided in groups, where they can discuss urine collection ideas and brainstorm new solutions. This can be by drawing/writing/verbal explanation or making something with available materials. At the end each group will be able to present the summary of their ideas to the rest of the participants and organisers.
- If workshops are held online. An online worksheet will be set up and teams will work on their ideas there. Teams will be asked to present their ideas at the end of the workshop.
- Notes will be taken and online worksheets will be kept by the research team for analysis and further work. No video or audio recording will be taken.
- Session will last between 2.5-3h

For peer review only - http://bmjopen.bmj.com/site/about/guidelines.xhtml

**3. Will the research include any audio, video or photographic recordings?**
Yes. Participants who don't wish to be recorded could still take part. In this case researcher will take notes during the interviews.
**4. Biological sample handling**
No biological samples will be taken as part of the study.
**5. Please detail all expenses or gifts that will be offered to participants.** Guidance is available in Best Practice Guidance: 05 Payments and incentives in research.
R~~No r~~emuneration will be given for participation in the study, in the form of £20 electronic gift vouchers for time taken to participate in interviews.~~.~~

SECTION F. ETHICAL CONSIDERATIONS

For guidance on ethical issues, please see http://researchsupport.admin.ox.ac.uk/governance/ethics/resources

(N.B. To complete, double click on the check boxes and select 'checked')

**1. Will the research involve any participants considered vulnerable in the context of the research (e.g. children, elderly, prisoners)?** **If yes,** please describe how they are defined as vulnerable and detail any CUREC Approved Procedures or guidance that will be applied to the research (for current documents and templates see https://researchsupport.admin.ox.ac.uk/governance/ethics/resources). **If yes, and you cannot apply any Approved Procedure**, please cease completion of this form – a CUREC 2 application is required	Yes ☐	No ☒
**2. Will unequal relationships exist between participants and those obtaining informed consent?** **If yes,** describe the nature of the unequal relationship and how arising ethical issues will be addressed	Yes ☐	No ☒
**3. Will the research involve questions and/or discussions of contentious and/or sensitive issues (e.g. information relating to ethnicity, political opinions, religious beliefs, physical/mental health or sexual life)?** **If yes,** please justify why this is required and provide a copy of the questionnaire raising the issues that will be used in your research.	Yes ☒	No ☐
Healthcare professionals: We know urine collection methods and procedure are not consistent throughout different clinical practices or clinicians. We would like to understand what drives the decision making. We know different countries and culture		

For peer review only - http://bmjopen.bmj.com/site/about/guidelines.xhtml

have different acceptability to invasive or non-invasive procedures, so would like to test whether ethnicity plays a role.<br><br>Parents:<br><br>We would like our study to be diverse and include people from different social-economic backgrounds and ethnic backgrounds. In order to quantify the success rate of these goals, and report them, we will ask parents/carers to state their ethnicity and questions that will allow us to estimate their social-economic position.<br><br>Workshop:<br><br>In order to be able to report who took part in this study, we will ask demographic questions. Participants will have the chance of not answering if they don't feel comfortable doing so.		
**4. Will the research involve deliberate deception of participants outside the scope of CUREC Approved Procedure 07?**<br><br>**If yes**, please cease completion of this form – a CUREC 2 application is required<br><br>**If no,** please either give details of the deception or state that no deception will be involved	Yes ☐	No ☒
**5. Could the proposed research affect your own physical and/or psychological safety as a researcher?**<br><br>**If yes,** describe how you will manage this.	Yes ☐	No ☒
**6. How will you ensure the research is conducted according to the details given in this form?**		
Members of the research team will meet weekly to discuss progress or issues and themes that are arising from the interviews in order to make decision about the continuity and direction of interviews. Moreover, these discussions will also support the data analysis. And if needed, another person will be review recordings to validate findings.<br><br>Any students involved in the study will form part of the research team and will present findings on weekly basis. Students will be supported in their analysis by senior researchers.<br><br>Procedures will be discussed during meetings. We have involved people with experience in qualitative studies, who will support the process. Additionally, senior members of the research team will be able to join interviews and analysis, in order to check procedures are followed.<br><br>If any participant has questions, they will be able to ask the research team directly. They will also be able to reach the principal investigator if they feel appropriate. We will acknowledge reception and will give an indication of how it will be dealt with. Any serious complaints will be forwarded to the Chair of the Research Ethics Committee at the University of Oxford (ethics@medsci.ox.ac.uk).		
**7. Please give details of any other research-specific ethical and/or safety considerations**		
None		
**8. How do you propose to deal with / handle any incidental findings?**		
Not applicable		

For peer review only - http://bmjopen.bmj.com/site/about/guidelines.xhtml

**9. Will any data or information from this study be provided to individual participants?**
No. The study aims to explore and validate challenges faced during urine collection. Therefore, there are no individual results to be provided.

SECTION G. OTHER CONSIDERATIONS

**1. Is any part of this research being conducted overseas?**<br>**If yes,** please give details below. Ensure you complete and submit a travel risk assessment form to your departmental safety officer. (This is necessary to ensure the travel/fieldwork is covered by the University's travel insurance – see http://www.admin.ox.ac.uk/finance/insurance/travel/)	Yes ☐	No ☒
**2. Does your research raise issues relevant to the Counter-Terrorism and Security Act (the Prevent Duty), which seeks to prevent people from being drawn into terrorism?**<br>**If yes,** please say how you plan to address any related risks. Please see advice on this on our Best Practice Guidance Web Page.	Yes ☐	No ☒

SECTION H. DATA MANAGEMENT AND HANDLING

All information provided by participants is considered **research data** for the purpose of this form. Any research data from which participants can be identified is known as **personal data**; any personal data which is sensitive is considered **special category data**.

Management of personal data, either directly or via a third party, must comply with the requirements of the General Data Protection Regulation (GDPR) and the Data Protection Act 2018, as set out in the University's Guidance on Data Protection and Research.

In answering the questions below, please also consider the points raised in the Data Protection Checklist and whether, for higher-risk data processing, a separate Data Protection Impact Assessment may also be required for the research. Advice on research data management and security is available from Research Data Oxford and your local IT department. Advice on data protection is available from the Information Compliance team.

1. Please mark 'X' against the data you will collect for your research

Screening documents	☐	Audio recordings	☒
Consent records including participant name or other identifiers (e.g. written consent forms, audio-recorded consent, assent forms)	☒	Video recordings	☐

For peer review only - http://bmjopen.bmj.com/site/about/guidelines.xhtml

Consent obtained anonymously (e.g. via online survey)	☐	Transcript of audio/video recordings	☒
Opt-out forms	☐	Photographs	☐
Contact details for the purpose of this research only	☒	Information about the health of the participant (including mental health)	☐
Contact details for future use (guidance)	☒	Physiological test results / measurements	☐
Field notes	☒	MRI scans	☐
Task results (e.g. questionnaires, diary completion)	☐	IP addresses (refer to Best Practice Guidance 09: Data collection, protection and management for guidance)	
Data already in the public domain.<br>Specify the source of the data:	☐	Other (please specify below)	☐
Previously collected (secondary) data	☐		

2. How and where will each type of data be stored whilst the research is ongoing (until the end of all participant involvement)?

List each type of data selected above, and explain how each will be physically transferred (including movement/sharing of audio files, paper records, electronic downloads etc.) from where it is collected to a suitable storage site (e.g. Nexus365 OneDrive for Business, SharePoint, University servers). State the storage location for each. Do not store unencrypted data in freely available cloud services or unprotected USB drives.

Refer to Best Practice Guidance on data collection, protection and management (BPG09).

Hard copies of data (consent forms and field notes) will be placed in a folder and transferred to an office at the Engineering Science Department as soon as possible. In the Engineering Science Department, consent forms will be stored in a locked filling cabinet. When, despite efforts, it is not practical to immediately store data in this location, a lockable filling cabinet in the researcher's home will be used and will be transferred to the Engineering Science Department as soon as possible.

All electronic data (typed field notes, audio recordings and personal data including contact details) will be transferred from the researcher's password protected device to a password-protected drive within the University network. The audio recording held by the researchers will then be deleted once the interview audio has been transcribed. These recordings will be securely transferred using the university file sharing system or Nexus OneDrive for business. Workshops work documents will be de-identified.

Identifying information from audio recordings will be removed from the transcripts. Transcription will be done by the researcher or approved University transcriber. De-identified transcripts may be uploaded into NVIVO 12 which will accessed by the research team on password-protected computers. This de-identified data may also be made available to other researchers.

The transcription will be stored as a Word file on encrypted computers within the University network.

3. Will you use a unique participant number on research data instead of participant name?

If yes, state whether or not you will retain a list of participant names against numbers (pseudonymisation via a linkage list).

Where will the list be stored, and when will it be destroyed?

Yes. Data identifiers will be labelled and stored securely and separately from the research data, on password protected computers. We will destroy participants information once research has been completed and published.

For peer review only - http://bmjopen.bmj.com/site/about/guidelines.xhtml

**4. Who will have access to the research data?**
Researchers listed on this form will have access to the research data and other student/researchers working on the study. Access will be granted to the MS IDREC for the purposes of monitoring and/or audit of the research.<br>De-identified data may be made available to other researchers.
**5. If research data is to be shared with another organisation, how will it be transferred / disclosed securely?**
Any data will be shared securely using the university file sharing system (OxFile or Nexus365 OneDrive Business system) and returned the same way. The data will be stored in password-protected computers within their institutions.
**6. When and how will identifiable data be destroyed or deleted?**<br>N.B. If any identifiable data will be retained beyond the end of the study and/or indefinitely, please state what data this is, and the reasons for retention (e.g. contact details for future studies; photos used in publication). This must be clearly stated on participant information, and specific consent obtained.
All identifiable data will be removed from the transcript.<br>Unique participants number will be used. Audio recordings will be destroyed after publication.<br>Consent records and de-identified transcripts and worksheets will be kept 3 years after publication.<br>Personal data will be deleted from servers after the analysis and publication has been completed.

**7. Please confirm that you will store other (non-identifiable) research data safely for at least 3 years after final publication or public release and adhere to any additional research funder policies.**<br>For more information about the University policies, please see the University's web pages on research data management.<br>**If 'Yes'**, please give details of who will store the data and on storage format, location and security.<br>**If 'No'**, please provide further details.	Yes ☒	No ☐
Non-identifiable data will be stored in a password-protected computer within the University network at the PI's department.		

SECTION I. PUBLICATION AND DISSEMINATION OF RESULTS

**1. How will you disseminate and feedback project outcomes at the end of the research?**	Study finding will be disseminated though publications in peer-reviewed journals, conferences and other articles such as for the Community Health MIC, Children and Young People MIC. Public journals or seminars.

For peer review only - http://bmjopen.bmj.com/site/about/guidelines.xhtml

	We will aim to make these findings available to the scientific and wider community by following the Open Science guidelines.

For peer review only

For peer review only - http://bmjopen.bmj.com/site/about/guidelines.xhtml

DECLARATIONS AND SIGNATURES

In providing signatures, the MS IDREC Secretariat will accept either:

Option 1: Email confirmations sent from a University of Oxford email address. Separate emails should be sent by each of the relevant signatories as outlined below, indicating acceptance of their responsibilities.

Option 2: That the form be fully-signed with handwritten (wet-ink) signatures. Please scan these and the rest of the form pages to create a single PDF document and email to us.

PRINCIPAL INVESTIGATOR (AND STUDENT IF APPLICABLE)

I/We, the researcher(s):

- Understand our responsibilities as outlined on this form and in the CUREC glossary and guidance
- Agree to start this research only after obtaining approval from the MS IDREC;
- Understand that the Principal Investigator must ensure that all researchers are suitably qualified and trained to conduct the research described, or are appropriately supervised until deemed qualified/trained;
- Agree to provide additional information as requested by the MS IDREC before approval is secured and as research progresses;
- Agree to maintain the confidentiality of all data collected from or about participants;
- Agree to notify the MS IDREC in writing immediately of any proposed change to the research, and await approval before proceeding with the proposed change;
- Agree to notify the MS IDREC if the Principal Investigator changes and supply the name of the successor;
- Will use the data collected only for the research for which approval has been given;
- Will grant access to data only to authorised persons; and
- Have made arrangements to ensure that personal data collected from participants will be held in compliance with the requirements of the GDPR and the Data Protection Act 2018.

For peer review only

**Principal Investigator (Name)**	Jeroen Bergmann
**Principal Investigator (Signature)**<br>Pasted images of signatures cannot be accepted	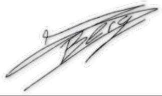
**Date**	30/06/2021
**Student (Name)**	
**Student (Signature)**<br>Pasted images of signatures cannot be accepted	
**Date**	

For peer review only - http://bmjopen.bmj.com/site/about/guidelines.xhtml

ACCEPTANCE BY HEAD OF DEPARTMENT/FACULTY OR DESIGNATED NOMINEE*

*Another senior member of the department may sign where the head of department is the Principal Investigator, or where the head of department has appointed a nominee. Example nominees include Deputy Head of Department, Director of Research, or Director of Graduate/Undergraduate Studies.

I have read the research proposal above. On the basis of the information available to me, I:

- consider the Principal Investigator/Supervisor and student researcher (if applicable) to be aware of their ethical responsibilities in regard to this research;
- am satisfied that the proposed design and scientific methodology are sound; the research has been subject to appropriate peer review and is likely to contribute to existing knowledge and/or to the education and training of the researcher(s) and that it is in the public interest.

**Head of Department or designated nominee (Name)**	
**Head of Department or designated nominee (Signature)**<br>Wet-ink signature (not pasted electronic image)<br>*or*<br>The Head of Department/nominee can send an email (including PI name and study title) to ethics@medsci.ox.ac.uk confirming the above	
**Date**	

For peer review only

For peer review only - http://bmjopen.bmj.com/site/about/guidelines.xhtml

Study Title: Exploring perspectives, priorities, and solutions of urine collection: A qualitative user centric study to understand practices and gain feedback on from healthcare professionals and parents/carers
Document Title: Topic Guide Parents/Carers
Date and Version Number: 20-Oct-2021, V0.1
CUREC Reference Number: R77332/RE001

Interview questions

This is a semi-structured interview. The questions below provide a guide and are not fixed. The interviewee can talk freely during the interview and the conversation will guide which questions will be asked next. The interview has 3 stages: warm-up questions, main questions, and wrap-up questions.

Reminder of how we will use the data:

> We will record the interview. This will be transcribed, and all identifiable information will be erased from transcription and stored safely within the Engineering Department. Recordings will be erased.

Introduction

Reassure interviewee that this is an interview to understand what works and what doesn't work of current urine collection methods so we can design technology that helps parents and clinicians.

Warm-up questions

We will give demographic questions to interviewee for them to fill. The option of prefer not to say will be given. Interviewer will remind participant that they do not need to answer any question they prefer not to answer.

- Tell us about yourself;
  - Who do you care for?
  - How many children do you have?
  - Demographic questions
    - Age
    - Gender
    - Ethnicity (give options from a list)
    - Social demographics:
      - Post code
      - How many people live in the house?
      - Employment status (full time / part time / stay at home parent / unemployed)
      - Number of dependents
    - Education level
  - How did you hear about this study?

Main questions

Deep dive into the urine collection process:

For peer review only - http://bmjopen.bmj.com/site/about/guidelines.xhtml

- Tell me about your experiences with urine collection in infants and toddlers?
  - When did it last happen?
  - Where did the urine collection occur? i.e. hospital, home, GP
    - If you sought medical attention, what were the symptoms your child had that worried you?
  - Tell me about how you were told to get the sample
    - How was the urine sample collected?
    - Were you given instructions? Do you remember them (the instructions you were given)? → Urine Collection Method
    - Who gave them to you?
    - Did someone explain why a urine sample was needed?
    - Was there any leaflet or other information that helped with the process?
      - If yes, do you remember any helpful information?
  - How did you find the process of urine collection in your child?
    - What were the challenges?
    - What worked well?

- When did this last happen?
- Did this happen with only one or many of your children? Does it happen often?
- How often do you perform urine collection in children?
- Are you aware that there are different urine collection methods?
  - Have you previously used other methods?
  - If you used other ways of collecting urine, which method did you prefer?
    - Why?
  - Did you have any worries about some of the collection methods?

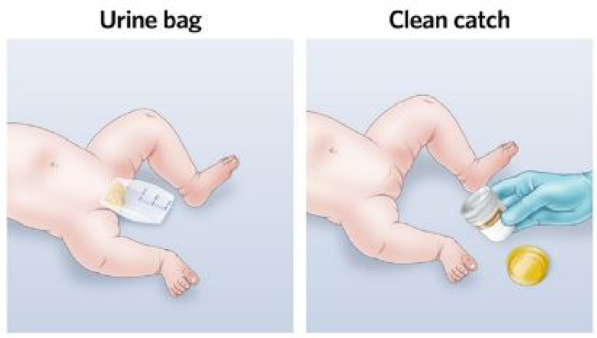

NON-INVASIVE

For peer review only - http://bmjopen.bmj.com/site/about/guidelines.xhtml

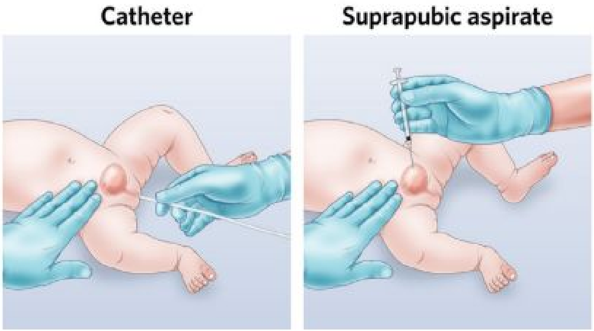

INVASIVE

Figure 1. Images by Dr. Jonathan Kaufman and Bill Reid, Royal Children's Hospital. Kaufman et al, BMJ, 2017. 357: 16-19.

- What is your opinion about the urine collection devices available?
  - Invasive vs. non-invasive, i.e. a sample requiring a nurse/doctor placing a urinary catheter versus getting the child to sit on a potty
  - If there was more than one option, were you given a choice?
- The NICE guidelines in UK suggest using the clean catch method. In your opinion was this the method used for your child.
  - Have you ever heard of ways to speed up collecting urine samples (eg gently tapping the bladder)?
    - If yes, did it work? / How long did it take?
- Are you aware that some urine samples are unreliable because they end being contaminated with bacteria (bugs) commonly found on our skin?
- Do you know of other urine collection devices?
- In your opinion:
  - what would the ideal process look like from beginning to end?
  - Where can/does the process go wrong?

Wrap-up questions

End with asking them if they have any questions, ensuring they are comfortable with everything that has been said, and whether they would be open to further interviews in the future:

- Would you like to add anything that we didn't ask about?
- Do you know anyone else that has gone through urine collection in a non-toilet trained infant that might be interested in participating?
- If I had any quick questions arise from reviewing my notes today, would it be possible to give you a quick call/email?
- Would you been open to further interviews from us in the future as we continue to develop this tool?

For peer review only - http://bmjopen.bmj.com/site/about/guidelines.xhtml

For peer review only

For peer review only - http://bmjopen.bmj.com/site/about/guidelines.xhtml

Study Title: Exploring perspectives, priorities, and solutions of urine collection: A qualitative user centric study to understand practices and gain feedback on from healthcare professionals and parents/carers
Document Title: Topic Guide Healthcare Staff
Date and Version Number: 20-Oct-2021, V0.1
CUREC Reference Number: R77332/RE001

DEPARTMENT OF ENGINEERING SCIENCE

UNIVERSITY OF OXFORD

Interview questions

This is a semi-structured interview. The questions below provide a guide and are not fixed. The interviewee can talk freely during the interview and the conversation will guide which questions will be asked next. The interview has 3 stages: warm-up questions, main questions, and wrap-up questions.

Introduction

Reassure interviewee that this is an interview to understand practices and knowledge around urine collection in order to design new solutions including technology, rather than judging or evaluating practices of individual.

Healthcare Professionals:

Warm-up questions

Interviewees will be asked to complete these questions in writing. Demographic questions will have an option "prefer not to say". Interviewer will remind participants that they do not need to answer any question they don't wish to answer.

All Healthcare Professionals

- Tell us about yourself
    - which hospital do you work in?
    - what is your role?
    - how long you have been in the role?
- Demographic questions
    - Age
    - Gender
    - Ethnicity
- How did you hear about this study?

If Medical Practitioners/Nurses

- About position
    - Main location of practice
    - Country of medical or nursing degree if applicable
    - Years of working in as current specialty (ie. As GP / in ED / Admin)
    - Training level
    - Do you know how many children under 2 would have a urine collection a week?
    - Specialised in paediatrics
    - Additional postgraduate qualification in paediatrics or child health
- Number of full-time equivalent GPs at clinic/number of doctors in ED
- Number of full-time equivalent nurses

For peer review only

- Type of clinic (private, mixed, NHS, other)
- What is your role related to urine collection?

Main questions

If Medical Practitioners/Nurses

Deep dive into the urine collection process:

- In the hospital you work in / have worked in, is there a preference for a particular urine collection method?
- What is your experience carrying out urine collection in infants? Have you used all methods?
- How often do you perform urine collection in children? / (in a day/week how many urine samples are collected?)
- Think about a recent experience carrying out/ordering/supervising/assisting a urine collection in an infant
  - Age of patient
  - Physical and emotional condition of patient / parent
  - Where was the collection done? Home, hospital, GP practice?
  - Who ordered the urine collection?
  - Where did the collection take place?
  - How many people were involved in the procedure?
  - Who carried out the collection?
  - How long did it take?
  - Any feedback?
  - Follow-up of patient
- Tell me about how would you go about asking parents to obtain a urine sample from their child?
  - Instructions:
    - ~~Have you been taught how to give~~Do you provide urine collection instructions for parents?
      - If no: why not? (lack of time, lack of knowledge, don't think it's necessary)
      - If yes, what type of instructions do you provide?
        - Do you think this is normal practice with colleagues? (allude to the fact that most parents have mentioned not to get instructions, would they agree?.
          - Why do you think that is?
    - ~~Did you share this information with parents?~~
  - Procedure explanation
  - Why are you collecting the sample?
- Who decides to get a urine sample?
  - If you, when would you get a urine sample?. Is this process clear?
- What is your perception of non-invasive urine methods as a diagnostic procedure?

Commented [MA1]: Here, trying to see, if they feel is an easy and valid method to diagnose an infection or if it's just a check list procedure.

- What is your opinion about the urine collection devices available?
  - Invasive vs. non-invasive

For peer review only

- What is the determining factor when choose a particular option?
- The NICE guidelines in UK suggest using the clean catch method. In your opinion is this the most common method used?
- Do you or your colleagues ever use voiding stimulating methods for clean catch (i.e. Quick-wee method, bladder-tapping method)?
    - If yes, what is the practice mostly used and your experience in terms of success rate, usefulness, difficulty
    - If not, why not?
- What do you think are the pros and cons of current urine collection devices/methods, if any?
    - Re-visits
    - Misdiagnosis
    - Contamination
    - Usability
- Do you know of other novel urine collection devices?
- Having considered everything mentioned before,
    - what would the ideal process look like from beginning to end?
    - Where can/does the process go wrong?

<u>If Bio-analyst</u>

Deep dive into the urine collection processes:

- Tell me about the work you do in regard to urine analysis in infants?
- How many urine collections happen in a month?
- How reliable are urine samples of infants for diagnosis of infections?
    - Do you have information of the method used for urine collection?
        - If yes, what do you think about invasive and non-invasive methods of urine collection?
        - How does clean catch compare?
        - Do you think there are other practices that reduce contamination rates
- Is retesting a frequent occurrence?
    - How much do you think this cost?
    - In your opinion what drives the use of one method or another?
- Having considered everything mentioned before,
    - what does the ideal process look like from beginning to end?
    - Where can/does the process go wrong?

<u>If admin staff</u>

Deep dive into the urine collection processes:

- Tell me about your experiences with urine collection in infants?
- How many urine collections happen in a month?
- What is the cost?
    - What are the resources used for a urine collection in infants
        - Duration for a collection
        - Use of room
        - Staff
    - How does clean catch compare to other methods?

For peer review only

  - In your opinion what drives the use of one method or another?
- The NICE guidelines in UK suggest using the clean catch method. In your opinion is this the most common method used?
  - Have you ever heard of voiding stimulating methods for clean catch (i.e. Quick-wee method, bladder-lumbar method)?
    - If yes, what is the success rate?
- Are you aware of contamination rates in different urine collection methods?
  - How does this affect cost?
- Do you know of other novel urine collection devices?
- Having considered everything mentioned before,
  - what does the ideal process look like from beginning to end?
  - Where can/does the process go wrong?

Wrap-up questions

End with asking them if they have any questions, ensuring they are comfortable with everything that has been said, and whether they would be open to further interviews in the future:

- Are there any questions you think I should have asked? Is there anything you would like to add?
- Who/where else do you think it might be interesting for us to interview? - How come? (because of different dynamic, scale and scope of work etc).
- If I had any quick questions arise from reviewing my notes today, would it be possible to give you a quick call/email?
- Would you been open to further interviews from us in the future as we continue to develop this tool?

Do you have any questions from us, would you like to know more about our urine collection project?

COREQ (COnsolidated criteria for REporting Qualitative research) Checklist

A checklist of items that should be included in reports of qualitative research. You must report the page number in your manuscript where you consider each of the items listed in this checklist. If you have not included this information, either revise your manuscript accordingly before submitting or note N/A.

Topic	Item No.	Guide Questions/Description	Reported on Page No.
**Domain 1: Research team and reflexivity**			
Personal characteristics			
Interviewer/facilitator	1	Which author/s conducted the interview or focus group?	
Credentials	2	What were the researcher's credentials? E.g. PhD, MD	
Occupation	3	What was their occupation at the time of the study?	
Gender	4	Was the researcher male or female?	
Experience and training	5	What experience or training did the researcher have?	
Relationship with participants			
Relationship established	6	Was a relationship established prior to study commencement?	
Participant knowledge of the interviewer	7	What did the participants know about the researcher? e.g. personal goals, reasons for doing the research	
Interviewer characteristics	8	What characteristics were reported about the inter viewer/facilitator? e.g. Bias, assumptions, reasons and interests in the research topic	
**Domain 2: Study design**			
Theoretical framework			
Methodological orientation and Theory	9	What methodological orientation was stated to underpin the study? e.g. grounded theory, discourse analysis, ethnography, phenomenology, content analysis	
Participant selection			
Sampling	10	How were participants selected? e.g. purposive, convenience, consecutive, snowball	
Method of approach	11	How were participants approached? e.g. face-to-face, telephone, mail, email	
Sample size	12	How many participants were in the study?	
Non-participation	13	How many people refused to participate or dropped out? Reasons?	
Setting			
Setting of data collection	14	Where was the data collected? e.g. home, clinic, workplace	
Presence of non-participants	15	Was anyone else present besides the participants and researchers?	
Description of sample	16	What are the important characteristics of the sample? e.g. demographic data, date	
Data collection			
Interview guide	17	Were questions, prompts, guides provided by the authors? Was it pilot tested?	
Repeat interviews	18	Were repeat inter views carried out? If yes, how many?	
Audio/visual recording	19	Did the research use audio or visual recording to collect the data?	
Field notes	20	Were field notes made during and/or after the inter view or focus group?	
Duration	21	What was the duration of the inter views or focus group?	
Data saturation	22	Was data saturation discussed?	
Transcripts returned	23	Were transcripts returned to participants for comment and/or	

For peer review only

Topic	Item No.	Guide Questions/Description	Reported on Page No.
		correction?	
**Domain 3: analysis and findings**			
Data analysis			
Number of data coders	24	How many data coders coded the data?	
Description of the coding tree	25	Did authors provide a description of the coding tree?	
Derivation of themes	26	Were themes identified in advance or derived from the data?	
Software	27	What software, if applicable, was used to manage the data?	
Participant checking	28	Did participants provide feedback on the findings?	
Reporting			
Quotations presented	29	Were participant quotations presented to illustrate the themes/findings? Was each quotation identified? e.g. participant number	
Data and findings consistent	30	Was there consistency between the data presented and the findings?	
Clarity of major themes	31	Were major themes clearly presented in the findings?	
Clarity of minor themes	32	Is there a description of diverse cases or discussion of minor themes?	

Developed from: Tong A, Sainsbury P, Craig J. Consolidated criteria for reporting qualitative research (COREQ): a 32-item checklist for interviews and focus groups. *International Journal for Quality in Health Care*. 2007. Volume 19, Number 6: pp. 349 – 357

Once you have completed this checklist, please save a copy and upload it as part of your submission. DO NOT include this checklist as part of the main manuscript document. It must be uploaded as a separate file.

For peer review only

BMJ Open

Clinician and parent views on urine collection in pre-continent children in the UK: a qualitative interview study

Journal:	*BMJ Open*
Manuscript ID	bmjopen-2023-081306.R1
Article Type:	Original research
Date Submitted by the Author:	21-Feb-2024
Complete List of Authors:	Armengol, Monica; University of Oxford, Department of Engineering Science<br>Hayward, Gail; University of Oxford, Nuffield Department of Primary Care Health Sciences<br>Abbott, Molly; Guy's and St Thomas' Hospitals NHS Trust<br>Bird, Chris; Birmingham Children's Hospital, Emergency Department<br>Bergmann, Jeroen; University of Oxford, Department of Engineering Science<br>Glogowska, Margaret; University of Oxford, Nuffield Department of Primary Care Health Sciences
**Primary Subject Heading**:	Paediatrics
Secondary Subject Heading:	Qualitative research
Keywords:	PAEDIATRICS, Urinary tract infections < UROLOGY, QUALITATIVE RESEARCH

SCHOLARONE™
Manuscripts

I, the Submitting Author has the right to grant and does grant on behalf of all authors of the Work (as defined in the below author licence), an exclusive licence and/or a non-exclusive licence for contributions from authors who are: i) UK Crown employees; ii) where BMJ has agreed a CC-BY licence shall apply, and/or iii) in accordance with the terms applicable for US Federal Government officers or employees acting as part of their official duties; on a worldwide, perpetual, irrevocable, royalty-free basis to BMJ Publishing Group Ltd ("BMJ") its licensees and where the relevant Journal is co-owned by BMJ to the co-owners of the Journal, to publish the Work in this journal and any other BMJ products and to exploit all rights, as set out in our licence.

The Submitting Author accepts and understands that any supply made under these terms is made by BMJ to the Submitting Author unless you are acting as an employee on behalf of your employer or a postgraduate student of an affiliated institution which is paying any applicable article publishing charge ("APC") for Open Access articles. Where the Submitting Author wishes to make the Work available on an Open Access basis (and intends to pay the relevant APC), the terms of reuse of such Open Access shall be governed by a Creative Commons licence – details of these licences and which Creative Commons licence will apply to this Work are set out in our licence referred to above.

Other than as permitted in any relevant BMJ Author's Self Archiving Policies, I confirm this Work has not been accepted for publication elsewhere, is not being considered for publication elsewhere and does not duplicate material already published. I confirm all authors consent to publication of this Work and authorise the granting of this licence.

For peer review only

Clinician and parent views on urine collection in pre-continent children in the UK: a qualitative interview study

Monica Armengol

Department of Engineering Science, University of Oxford

Gail Hayward

Nuffield Department of Primary Care Health Sciences, University of Oxford

Molly Abbott

Guy's and St Thomas' Hospital NHS Trust, London

Christopher Bird

Birmingham Women's and Children's NHS Foundation Trust and Nuffield Department of Primary Care Health Sciences, University of Oxford

Jeroen Bergmann

Department of Engineering Science, University of Oxford

Department of Technology and Innovation, University of Southern Denmark

Margaret Glogowska

Nuffield Department of Primary Care Health Sciences, University of Oxford

Corresponding Author:

Margaret Glogowska

Nuffield Department of Primary Care Health Sciences, University of Oxford

Radcliffe Observatory Quarter, Woodstock Road

Oxford OX2 6GG, UK

Tel: +44 (0)1865 617872

Email: Margaret.glogowska@phc.ox.ac.uk

For peer review only - http://bmjopen.bmj.com/site/about/guidelines.xhtml

Abstract

Objective

To explore the experiences of health care professionals (HCPs) and parents of urine collection methods, to identify barriers to successful sampling and what could improve the process.

Design

Qualitative research, using individual semi-structured interviews with HCPs and parents. The interviews were audio-recorded, transcribed and thematically analysed.

Setting

UK-based HCPs from primary and secondary care settings and parents with experience of urine collection in primary and/or secondary care settings.

Participants

HCPs who were involved in aiding, supervising or ordering urine samples. Parents who had experience of urine collection in at least one pre-continent child.

Results

13 HCPs and 19 parents were interviewed. Two participating HCPs were GPs, 11 worked in paediatric secondary care settings (eight were nurses and three were doctors). Two parents had children with underlying conditions where frequent urine collection was required to rule out infections. HCPs and parents reported that there were no straightforward methods of urine collection for pre-continent children. Each method – 'clean catch', urine bag and urine pad – had limitations and problems with usage. 'Clean catch', regarded as the gold standard by HCPs with a lower risk of contamination, often proved difficult for parents to achieve. Other methods had elevated risk of contamination but were more acceptable to parents because they were less challenging. Many of the parents expressed the need for more information about urine collection.

Conclusions

Current methods of urine collection are challenging to use and may be prone to contamination. A new device is required to assist with urine collection in pre-continent children, to simplify and

For peer review only - http://bmjopen.bmj.com/site/about/guidelines.xhtml

reduce the stress of the situation for those involved. Parents are key partners in the process of urine collection with young children. Meeting their expressed need for more information could be an important way to achieve better quality samples while awaiting a new device.

Strengths and limitations of this study

- We interviewed parents familiar with several of the different types of urine collection method and varied lived experience of urine collection.
- Most of the HCPs interviewed worked in environments where unwell children presented every day and where urine collection was a routine part of assessment.
- Interviewing both parents and HCPs gave us a dual perspective on the urine collection process.
- Our study includes relatively small samples of both parents and HCPs.

Patient and Public Involvement and Engagement Statement

This study emerged from an ideation workshop on challenges to urine collection in pre-continent children hosted by the NIHR Community Healthcare MIC in Oxford, UK, May 2019. The Covid-19 pandemic delayed the study's start but found unanimous approval at a virtual meeting of the NIHR Community Healthcare MIC's Patient and Public Involvement and Engagement (PPIE) for children and young people's health in October 2022 and ongoing support at meetings in July 2023 and January 2024.

For peer review only

Background

Urinary tract infections (UTIs) are common bacterial infections in young children.(1) Diagnosis is challenging as symptoms typical of UTI in adults may be absent.(2) In one study, fewer than one third of children who presented to primary care with acute illness and met microbiological criteria for UTI had been clinically suspected of having a UTI.(3) Thus, there is a need for urine testing to exclude UTI in children where the cause of acute illness is unclear.(4)

In the UK, invasive testing methods to collect urine in pre-continent children such as catheterisation may be used in emergency settings where time is critical but suprapubic aspiration (SPA) under ultrasound guidance is rarely performed. There is debate around whether invasive methods should be used more,(5) although there is evidence that SPA is more painful in infants than catheterisation.(6) UK guidelines emphasise the use of non-invasive methods and recommend invasive methods only if non-invasive methods are not possible or practical.(7) However, non-invasive methods such as ‘clean catch’, urine bags and nappy pads to collect urine in pre-continent children are challenging to use and associated with high rates of contamination.(4,8,9,10) Contamination of samples presents a significant clinical problem in that it delays diagnosis, can lead to inappropriate prescription and overuse of antibiotics, requires repeat samples which adds healthcare costs and delays discharge, and can result in further unnecessary diagnostic tests such as renal ultrasound scans.(10)

A necessary step toward improving urine collection for pre-continent children is to understand current experiences and how things could be improved. Primary care Health Care Professionals’ (HCPs’) views of urine collection methods for pre-continent children have been explored using qualitative research methods.(4,11) However, the more holistic picture, including the views of secondary care HCPs, often the first contact for unwell children, as well as those of parents, have not been explored.

For peer review only - http://bmjopen.bmj.com/site/about/guidelines.xhtml

Aims

The aims of this study were:

- to understand the experiences and perceptions of HCPs and parents of urine collection methods in primary and secondary care settings
- to identify barriers to successful urine sampling in pre-continent children and what could make the process more efficient

Methods

Design

We chose a qualitative research design – individual semi-structured interviewing – for our study. This design captures people's detailed accounts of their experiences and perceptions of phenomena. It allows researchers to explore their selected topics but gives participants the opportunity to raise issues significant to them. Ethical approval was granted by the Central University Research Ethics Committee, University of Oxford (R77332/RE002) (Supplementary File 1).

Patient and Public Involvement

This study emerged from an ideation workshop on challenges to urine collection in pre-continent children hosted by the NIHR Community Healthcare MIC in Oxford, UK, May 2019. The Covid-19 pandemic delayed the study's start but found unanimous approval at a virtual meeting of the NIHR Community Healthcare MIC's Patient and Public Involvement and Engagement (PPIE) for children and young people's health in October 2022 and ongoing support at meetings in July 2023 and January 2024.

Recruitment and sampling

For peer review only - http://bmjopen.bmj.com/site/about/guidelines.xhtml

For peer review only

We invited HCPs and parents to participate. For HCPs we sought to include secondary care doctors and nurses, as well as general practitioners. Inclusion criteria included aiding, supervising or ordering urine samples in paediatric patients and being able to be interviewed in English. For parents, the inclusion criteria were having experience of urine collection in a pre-continent child within the two years prior to the interview and being able to be interviewed in English. This time period was sufficiently narrow to allow recall of events, but wide enough to ensure that enough parents could be recruited.

Recruitment took place through advertisements on social media groups of parents, charities, and paediatric organisations for HCPs; in newsletters around universities; on posters in nurseries; and on public news boards. The main recruitment material was a leaflet and poster which directed potential participants to the study website. Here they could find more information and register interest. They were then contacted by a research team member (MAr or MAb) to schedule an interview. Participants received written information about the study and had the opportunity to ask questions. Participants gave written consent before interviewing took place.

For peer review only

Data collection

The interviews with HCPs and parents were conducted using separate topic guides around three main issues – the methods and process of urine collection experienced, difficulties encountered, and how urine collection could be made easier. The topic guides were employed flexibly, allowing the interviewers to follow up issues raised by participants, and modified as the study progressed (Supplementary Files 2&3). We did not present any information to the parents about techniques or methods, we gave them the opportunity to tell us in their own words about their experiences. The interviews were conducted online using Microsoft Teams (by MAr and MAb) between December 2021 and July 2022. The mean time for the parent interviews was 35 minutes (range 18-65 minutes) and the mean time for the HCP interviews was 42 minutes (range 25-59) minutes. Methodological

For peer review only - http://bmjopen.bmj.com/site/about/guidelines.xhtml

oversight was provided by an experienced health services researcher (MG). Data collection ended when the researchers agreed that no new issues were raised in the interviews and there was sufficient understanding of the emerging categories.

Data analysis

Interviews were audio-recorded and transcribed. Transcripts were checked against the audio recordings, de-identified and uploaded to NVivo 12 (Lumivero, Denver, USA), a qualitative analysis software tool. To explore the range of experiences we encountered in the interviews, thematic analysis was used. Data driven analysis allowed us to identify the topics emerging from the HCP and parent data and group them into themes. Initial analysis of both sets of interviews was undertaken by MAr, and an iterative approach to coding, grouping codes into categories and subsequently themes, was taken.(12) MAb and MG reviewed the preliminary themes generated and refined these. These themes and sub-themes were then shared with the wider research team, discussed and agreed to ensure that they were credible and dependable, to enhance trustworthiness.(13,14)

Findings

Sample

42 people expressed interest in participating through the website or email, but not all responded to contact from the research team. Altogether, the sample included 13 HCPs and 19 parents. Among the HCPs, two of the participants were GPs, eight were nurses working in paediatric secondary care settings (including children’s Emergency Departments and other paediatric wards/departments) and three were doctors working in paediatric secondary care settings (including Children’s Emergency department, paediatric surgery and a paediatric department). All HCPs who participated were practising in the UK. Working experience in their current role varied from 7 months to over 30 years. Among the parents, eight of the parents had one child and the other eleven had two. Two of the parents had children with underlying conditions where frequent urine collection was required to rule

For peer review only - http://bmjopen.bmj.com/site/about/guidelines.xhtml

out infections. All but one of the parents interviewed were female. The parents were between 25 and 56 years old. All our participants identified as white, except three who identified as mixed race or Indian.

From the analysis of the interviews, two main themes emerged: acceptability and challenges of urine collection methods; and making the urine collection process more parent- and child-friendly.

<u>Acceptability and challenges of urine collection methods</u>

<u>*Invasive methods*</u>

HCPs and parents agreed that invasive urine collection methods, such as in and out catheterisation and suprapubic aspiration (SPA), were acceptable when a child was seriously unwell and a urine sample was urgently needed. For parents, SPA seemed the least acceptable method because they perceived it as painful for their child. Similarly, HCPs tended to regard SPA as the most invasive method and did not commonly perform it. One clinician regarded it as useful only if imaging was used to guide collection of the sample. Four of the secondary care clinicians performed catheterisation but this happened infrequently. Both parents and HCPs were aware that invasive methods had additional risks, particularly introducing infection, limiting their acceptability.

<u>*Non-invasive methods*</u>

Clean catch

Among the non-invasive urine collection methods, 'clean catch' was preferred in the primary and secondary care settings where the HCPs we interviewed worked and the parents we interviewed sought care for their children. This usually involves direct catching of urine spontaneously produced by the child into a sterile container such as a pot or a bowl provided for that purpose. Several HCPs described this process as a *"nightmare"* with pre-continent children, acknowledging that it can be demanding for parents, messy, difficult to judge and time-consuming.

For peer review only - http://bmjopen.bmj.com/site/about/guidelines.xhtml

Parents found the 'clean catch' process challenging, if not impossible. They highlighted how emotional the process could be, also making it less acceptable. Very young unwell children often became upset at the process which further increased the stress for parents. The 'clean catch' method often required the child to be unclothed, at the least have their nappy removed, so the parent could see when urination occurred. Some parents and children found this aspect very upsetting. Even adapting the method to obtain a sample at home, such as holding a child over the bath, could be distressing. Knowing that they needed to obtain a sample to help pinpoint why their child is ill but having little control over when and if they can get one, increased the stress felt by parents. HCPs concurred that using the 'clean catch' method often meant that samples could be very delayed.

HCPs acknowledged that while the clean catch method was pursued to avoid contamination, the reality of attempting it diverged from the ideal. They were aware that sterile containers provided did not always remain sterile and that it was difficult, if not impossible, for parents to prevent the container coming into contact with the child's skin. Some parents were aware of how contamination could occur but found it difficult to avoid. To reduce contamination from a 'clean catch' sample, several HCPs suggested that HCPs advise parents to wipe their child's genital area beforehand. Parents, however, reported not having received this instruction.

Urine bags

HCPs, particularly the GPs in our sample, and some parents, mentioned urine bags for urine collection but these were not used in all of the settings where our participants either worked or attended with their children. These are plastic bags placed around the child's genitalia using an adhesive which sticks to the skin. HCPs found the idea acceptable in principle as they caught urine without parents needing to follow their child around with a container. However, HCPs highlighted

For peer review only - http://bmjopen.bmj.com/site/about/guidelines.xhtml

issues such as high contamination rates, leakage through loss of adhesion and difficulty with fitting the bag around the genitalia of both boys and girls, making them far less acceptable. Parents who hadn't used urine bags were optimistic about trying them. However, parents who had already used them found them far less acceptable in practice. They described similar issues to those of the HCPs – contamination, leakage, the bag losing adhesive and dropping off, and discomfort when the bag was pulled away from the child's skin in sensitive areas.

Urine pads

Normally used for children younger than 6 months, urine pads were mentioned less frequently than 'clean catch' and urine bags. These are absorbent pads positioned inside the child's nappy from which urine is collected via a syringe or squeezed out. HCPs regarded them as acceptable but acknowledged their problems, which included higher contamination rate than 'clean catch' and difficulty knowing whether any urine has been collected. Few parents had experience of urine pads for urine collection. A couple of parents mentioned using improvised 'pads' of sponge or cotton wool when desperate to obtain a sample at home, acknowledging that this was not ideal.

Table 1 contains a summary of this theme **Acceptability and challenges of urine collection methods**. Table 2 summarises the acceptability criteria for non-invasive urine collection methods discussed by parents and HCPs. For HCPs the acceptability of a non-invasive method of urine collection was largely determined by contamination risk. This appeared to be the case for both primary and secondary care clinicians. In the primary and secondary care settings where the HCPs we interviewed worked, invasive methods tended to be used very infrequently and much less often than the default 'clean catch' method. Both sets of clinicians, however, acknowledged that this method often presented a considerable challenge for parents.

For peer review only

For peer review only - http://bmjopen.bmj.com/site/about/guidelines.xhtml

For parents, charged with obtaining the sample, acceptability was centred more around the difficulty of the method, the time taken and whether it caused their child distress.

Making the urine collection process more parent- and child-friendly

Communication between parents and HCPs

Parents often reported a lack of clear communication about urine collection from HCPs. Some parents felt they lacked an explanation of why a urine sample was required, while other parents felt it was only partially explained. Parents also suggested that HCPs assumed that they would know how to perform 'clean catch'. In one situation a parent was given a urine bag to use at home but needed to find instructions on the Internet. Parents often felt concern that they were obtaining samples correctly. Some were aware of contamination but lacked a step-by-step explanation of where it occurs and how to avoid it.

Some of the HCPs acknowledged spending little time giving parents full instructions. When asked if it was standard procedure for HCP to give parents instructions, one HCP thought there should be policies and written standards. Additionally, this HCP felt it was important to have guidelines around when to collect urine samples, so that parents were not burdened with it unless a sample was necessary for clinical decision-making.

Information for parents

HCPs and parents were asked about what could improve communication about urine collection. They agreed that formal instructions in a leaflet provided along with the urine collection container would be useful. HCPs were positive about giving parents written instructions as there was little time to pass on verbal instructions. Posters in clinical rooms where parents could read information were also viewed positively and one HCP suggested making a video parents could watch.

For peer review only - http://bmjopen.bmj.com/site/about/guidelines.xhtml

Parents and HCPs were asked about acceleration methods – actions which encourage or speed up urination. Mostly HCPs just recommended giving the child regular drinks but some were aware of other methods, such as turning taps on in the background, running a cool fan near the child, pouring warm water over the child's abdomen, and putting either warm or cool wet paper towels above their pubic area (Quick-Wee method).(15) Some HCPs found the tap method gave good results but was not practical, as it required a number of people to be involved. These methods were normally suggested only after time had elapsed without a sample collected. Few parents reported receiving any tips about speeding up urination. One parent accidentally found that as soon their child's nappy was removed and he stood on a cold floor urination happened. Parents, however, felt they would have appreciated suggestions from HCPs around acceleration methods even if they did not guarantee success.

Towards a new device design

Both parents and HCPs agreed on the need for a new device to simplify urine collection and reduce the stress of obtaining samples. Parents appreciated that no device could automatically reduce waiting time for urination but that it should enable clean, efficient collection when urination did occur. Parents were highly critical of the containers provided for urine collection, especially narrow pots designed for adults which they knew adults also struggle with. They felt that any new device using a container should be tailored according to sex and be wider to allow easier collection without touching the skin.

Parents and HCPs described the criteria they considered important in making a new device fit for purpose. A high priority for parents was a non-invasive device which didn't require constant monitoring. Parents and HCPs described how current methods could be adapted to make urine collection easier, such as a urine bag which doesn't need adhesive to stay in place. One HCP described how something like a nappy which would fit on the child would be helpful. Parents also

For peer review only - http://bmjopen.bmj.com/site/about/guidelines.xhtml

favoured a wearable device for urine collection from a child resting or lying down. Several parents responded very negatively to removing their child's nappy and other clothing during the wait for a sample, especially where this caused distress. Thus, a device which could be used while the child remained clothed was considered more appropriate. Secondary care HCPs concurred that such a device would reduce the need for private spaces during the process.

HCPs perceived that simplicity of use was crucial in a new device so that staff could learn to use it quickly. Cost and any need for training in using a new device were also major considerations. Disposal was considered important by both parents and HCPs but both groups accepted that any device would probably need to be single use plastic as sterilisation was not likely to be possible.

Table 3 contains a summary of this theme **Making the urine collection process more parent- and child-friendly**.

Discussion

Invasive methods have a place in urine collection with pre-continent children but non-invasive methods are more commonly experienced in both the primary and secondary care settings, from which we drew our sample. We found that there were no straightforward methods of urine collection for pre-continent children. HCPs and parents explained that each method described – 'clean catch', urine bag and urine pad – had limitations and problems with usage. 'Clean catch', the urine collection method regarded as the gold standard by HCPs and with a lower risk of contamination, often proved difficult for parents to achieve. Other methods had elevated risk of contamination but were more acceptable to parents because they were less challenging and are still advocated as a last resort by UK national guidelines.(7)

For peer review only - http://bmjopen.bmj.com/site/about/guidelines.xhtml

Our findings highlighted ways in which current urine collection practice could be improved for parents and pre-continent children. While there is evidence that quicker sample collection can occur by the 'Quick-Wee' method,(15) acceleration methods did not appear to be routinely practised by the HCPs we interviewed, as they required a number of staff members to be involved,(16) and parents were typically not informed about them. Although GPs who had tried this method considered it impractical, such information could potentially improve the urine collection process for parents.(2) Parents would have appreciated clearer communication from HCPs about why a urine sample is necessary and they often expressed the need for more information and support for collecting urine samples, as these may determine the management of their child's illness. Explanation of the ways in which contamination can occur and how to avoid this would have been welcome to parents. Leaflets, posters and information videos were ways mentioned to give parents greater awareness of the issues around urine collection. This has a parallel with the information needs highlighted in a study conducted with adult women experiencing UTIs.(17)

Our findings also demonstrate the need for improved urine collection methods, such as a device which could make the urine collection process less challenging for parents and less distressing for the children. Previous literature, detailing the strengths and shortcomings of various methods of urine collection, including all of those discussed in our study, also points towards the need for new methods.(2,4,11)

Strengths and limitations

Our study includes relatively small samples of both parents and HCPs who presented for interview. However, the sample includes a number of parents familiar with several of the different types of urine collection method and varied lived experience of urine collection. Similarly, the majority of the HCPs worked in environments where unwell children presented every day and where urine collection was a routine part of assessment.

For peer review only - http://bmjopen.bmj.com/site/about/guidelines.xhtml

Previous qualitative research has been conducted around UTI in children, but has focused on parents' symptom recognition and management of the condition rather than the experience of urine collection.(1) One study has explored the views of GPs about urine collection in pre-continent children and described the barriers and facilitators to the process in Australian primary care settings.(11) In our study we were able to explore the views of parents, GPs and secondary care clinicians, which has been lacking in the literature up until this point. Thus, we were able to achieve a dual perspective on urine collection. Comparing and contrasting the expectations and experiences of HCPs and parents about what made methods more or less acceptable (risk of contamination, ease of collection, the amount of time taken for urine collection and urine collection causing distress to the child) helped us to identify where mismatches occurred and suggest why.

A limitation of the study is the method of recruitment of the parent sample. Ideally, we would have recruited parents at the point of needing to collect a urine sample from their child. However, this was not possible due to time and funding constraints, as well as concerns about the feasibility of recruiting in busy urgent care settings.

Conclusions

Current methods of urine collection are challenging to use and may be prone to contamination. There is need for a new device to assist with urine collection in pre-continent children. Ideally, this would simplify and reduce the stress of the situation for those using it whilst reducing the contamination risk. Parents are key partners in the process of urine collection with young children. Meeting their need for more information and support in the process of collecting urine could be an important way to achieve better samples while waiting for a new device.

For peer review only - http://bmjopen.bmj.com/site/about/guidelines.xhtml

References

1 Campbell A, Hartling L, Louie-Poon S, Scott SD. Exploring the Experiences and Information Needs of Parents Caring for a Child With a Urinary Tract Infection: A Qualitative Study. *J Patient Exp*. 2021;8:1-8.

2 Boon H, Van den Bruel A, Verbakel J. GPs' perspectives on diagnosing childhood urinary tract infections: a qualitative study. *Br J Gen Pract.* 2022; DOI: https://doi.org/10.3399/BJGP.2021.0589

3 Butler CC, O'Brien K, Pickles T, et al. Childhood urinary tract infection in primary care: a prospective observational study of prevalence, diagnosis, treatment, and recovery. *Br J Gen Pract.* 2015; DOI: 10.3399/bjgp15X684361.

4 Boon HA, Lenaerts W, Van Aerde C, Verbakel JY. Outpatient urine collection methods for paediatric urinary tract infections: Systematic review of diagnostic accuracy studies. *Acta Paediatr.* 2021;110:3170– 3179. https://doi.org/10.1111/apa.16027

5 Cousin E, Ryckewaert A, de Jorna Lecouvey C, Arnaud AP. Urine collection methods used for non-toilet-trained children in pediatric emergency departments in France: A medical practice analysis. *Archives de Pédiatrie*. 2019;26:16–20.

6 Kozer E, Rosenbloom E, Goldman D. Pain in Infants Who Are Younger Than 2 Months During Suprapubic Aspiration and Transurethral Bladder Catheterization: A Randomized, Controlled Study. *Pediatrics*. 2006;118, 1:e51-e56.

7 NICE, Urinary tract infection in under 16s: diagnosis and management NICE guideline Published: 27 July 2022 www.nice.org.uk/guidance/ng224

8 Butler CC, Sterne JA, Lawton M, et al. Nappy pad urine samples for investigation and treatment of UTI in young children: the 'DUTY' prospective diagnostic cohort study. *Br J Gen Pract.* 2016;66(648):e516-524.

9 Tosif S, Kaufman J, Fitzpatrick P, et al. Clean catch urine collection: Time taken and diagnostic implication. A prospective observational study. *J Paediatr Child Health*. 2017;53:970–975.

For peer review only - http://bmjopen.bmj.com/site/about/guidelines.xhtml

10 O'Gorman J, Clarke J, Robinson M. 'Spending a penny, but keeping it clean' – reducing the rate of contaminated non-invasive samples in the paediatric department. *Arch Dis Child.* 2022;107(Suppl 2):A429–A430.

11 Kaufman J, Sanci L, Temple-Smith M. What's the catch? Urine sample collection from young pre-continent children: a qualitative study in primary care. *BJGP Open*. 2020; DOI: 10.3399/bjgpopen20X101060.

12 Silverman D. *Doing qualitative research: a practical handbook.*2018. London: Sage Publications: 2018.

13 O'Brien BC, Harris IB, Beckman TJ, Reed DA, Cook DA. Standards for reporting qualitative research: a synthesis of recommendations. Acad Med. 2014;89(9):1245-1251.

14 Lincoln YS, Guba EG. *Naturalistic inquiry.* 1985. Beverly Hills, CA: Sage Publications; 1985.

15 Kaufman J, Fitzpatrick P, Tosif S, et al. Faster clean catch urine collection (Quick-Wee method) from infants: randomised controlled trial. BMJ. 2017;357(j1341):1–8.

16 Herreros Fernández ML, González Merino N, Tagarro García A, et al. A new technique for fast and safe collection of urine in newborns. *Arch Dis Child.* 2013;Jan;98(1):27-9. doi: 10.1136/archdischild-2012-301872.

17 Glogowska M, Croxson C, Hayward G. Women's information needs around urine testing for Urinary Tract Infections: qualitative study. *Br J Genl Pract. 2022;*DOI: https://doi.org/10.3399/BJGP.2021.0564

For peer review only - http://bmjopen.bmj.com/site/about/guidelines.xhtml

Tables

Table 1 Summary of theme 'Acceptability and challenges of urine collection methods'

Subtheme	Topic and example quotations
Invasive methods	***Suprapubic aspiration and catheterisation***<br>But if you've got a very unwell baby and there's a time pressure to collect a sample, then essentially you either have to get a catheter sample, or if absolutely required, then very occasionally they would do a suprapubic aspiration [Interview 562, nurse, children's Emergency Department [ED]]<br><br>I would quite happily have allowed them to catheterise her…because she was very poorly…and if that could have meant that we just got some urine out and got an answer and could go home I would have taken that option. I don't think I would want that on the other occasions because it's not particularly nice for a child to have a catheter [Int 600, parent]<br><br>I haven't done a suprapubic aspirate for absolutely years and years…but generally, if you don't do them with imaging to help you then it's a bit pointless. [Int 767, consultant, children's ED]<br><br>***Risks around invasive methods***<br>If life or health could be permanently damaged by waiting an hour, then do what you need to do now. But obviously, it wouldn't have been ideal because…I'm sure using a catheter presumably carries some risk of infection and damage. [Int 869, parent]<br><br>If you were doing [an] invasive technique on everyone then you also put them at risk of introducing infection. They might not have one in the first place and then you can use a poor technique they're going to end up with one [Int 327, paediatric nurse, ED]
Non-invasive methods	***Challenges of the 'clean catch' method***<br>It can take ages and they [parents] missed the urine quite a bit. It ends up on the floor, on the bed, so it's not easy. [Int 984, advanced clinical practitioner, children's ED]<br><br>I mean, they're wildly, wildly unpredictable. And yeah, it is very tiring. It is very challenging because obviously, they don't wee all that frequently, so if you miss it, then it really delays their care. [Int 298, paediatric surgeon, secondary care]<br><br>She [was] really quite distressed trying to put [her] in a position, trying to catch the urine for them…She's getting upset screaming at me. I'm trying to catch it…she keeps kicking the bowl… and it's just an absolutely nightmare [Int 535, parent]<br><br>It wasn't warm…she had to be naked and she was poorly…I thought well, if I put the nappy on, I wouldn't know when she's weeing to be able to then act quickly to move her on top of the pot that was given. So I felt quite bad as a mum when she's crying and unwell and laying there really cold and you're having to try and just get on with it to try and get one [a sample]. [Int 266, parent]<br><br>I have a bit of a blurry memory of holding a baby over a bath at three in the morning. With the husband holding a pot trying to wait for a wee basically…you've done a feed and you're just waiting for pee…she was not happy to be held…held naked just kind of held a bit awkwardly for about 10, 15 minutes. She was not happy about that. She was crying quite a lot. [Int 088, parent]<br><br>There was a kind of time pressure…we need this checked off. And the sooner I can get the sample the sooner we can exclude that there's anything really dangerous going on. [Int 869, parent]

For peer review only - http://bmjopen.bmj.com/site/about/guidelines.xhtml

Have the child out of a nappy and catch it when they wee, which obviously spends a long, long time…but then it means that everything is delayed. [Int 630, paediatric urology nurse, secondary care]

Contamination and 'clean catch'

But if you're sat there with the sterile pot for any period of time, it's gonna get touched by… the parent's finger. [Int 998, paediatric nurse, secondary care]

With the pot you've got to keep it very close to them, they've got to make sure that they're trying not to touch it [Int 980, advanced nurse practitioner, children's ED]
trying to not allow, the genitalia to touch the funnel, I don't think it's achievable. [Int 562, senior nurse, children's ED]

So I have had the words "get a sterile sample"… and I just sort of think well that's really hard, especially when I was having to do it in such a way when…she was lying down and you were trying to hold a little collection tube or something under. It was touching, you try not to touch the skin or anything, but you kind of have to, and then…I knew it wasn't clean, like as clean as they would want it, because it just it was just impossible. [Int 535, parent]

We've got some wipes, you want to clean the area before we wait particularly, you know, mainly the girls really the girls are worse. [Int 016, paediatric registrar, secondary care]
Nobody tells you make sure their bum is really clean or gives you anything to clean their bum with before they sit in this bowl [Int 307, parent]

Experiences around urine bags

The bags were much easier. You were guaranteed to catch, to capture it, you could put the nappy back on. They can run around, you can put them back in the waiting room. [Int 984, advanced clinical practitioner, children's ED]

There's the little bags that you stick that go inside the nappy but I haven't used them once in my job now…but from what I've been told by colleagues they notoriously don't stick. The urine leaks out of it, also they're not necessarily as sterile. [Int 543, nurse, children's ED]

Especially that bag, I would have given it a go. I suppose all children are different but…I think that bag one probably would work better than just looking out for wee and when she's going and having it [a pad under her] on the floor ready. [Int 266, parent]

It's not simple either [using the bag], because, it does stick on her and I think when you kind of peel it off, it leaves like a residue on her, so I think even like peeling it off at the end is a little bit probably a little bit painful, kind of like a bandaid, on those sensitive parts coming off probably does not feel good for her [Int 698, parent]

Experiences around urine pads

We can't do it that way because it's not a clean sample. So then I'll explain to them like if it does come up as positive for infection, and it's not a clean sample, we then have to repeat the sample anyway. [Int 327, paediatric nurse, ED]

Pads are not quite so easy because you can't tell that they've been used without looking at them… and in the past it just used to be just like I have to touch the nappy to see whether it was wet or not [Int 630, paediatric urology nurse, secondary care]

I used a new sponge and I cut it in half and I just put it between her legs and put a pair of pants on rather than a nappy…then when I felt she'd weed on me, I quickly grabbed the sponge and just wrung it out…I know that was probably classed as contaminated in some way [Int 266, parent]

For peer review only - http://bmjopen.bmj.com/site/about/guidelines.xhtml

Table 2 Acceptability criteria for urine collection using non-invasive methods in young children for HCPs and parents

	HCPs	Parents
**Preventing contamination in urine samples**	Highly important in determining acceptability of the method.<br><br>Uncontaminated samples necessary to guide diagnosis and treatment. 'Clean catch' recommended for this reason.	Not always aware of the importance of uncontaminated samples so less important in determining acceptability of the method.
**Ease of collection**	HCPs not involved in collection process so do not always experience the difficulties encountered by parents.<br><br>HCPs aware that 'clean catch' is challenging but still consider it the best option.	Highly important in determining acceptability of the method.<br><br>'Clean catch' perceived as very difficult and thus other methods, seen as less difficult but where contamination rates are higher, may be preferred.
**Amount of time taken for urine collection**	Important for HCPs as delay in urine collection can delay diagnosis/treatment.<br><br>HCPs aware that 'clean catch' can take a long time but still consider it the best option.	Very important as urine collection process can be lengthy.<br><br>'Clean catch' can require vigilance over long periods of time from parents. This can be particularly challenging if urine collection taking place in hospital.
**Urine collection causing distress to child**	HCPs not involved in urine collection process so do not always experience the distress caused to the child first hand.	Very important as child's distress, especially if undressing is required, during urine collection process adds to parental stress.

For peer review only - http://bmjopen.bmj.com/site/about/guidelines.xhtml

For peer review only

Table 3 Summary of theme 'Making the urine collection process more parent- and child-friendly

Subtheme	Topic and example quotations
Communication between parents and HCPs	***Explanation of the need for a urine sample***<br>It seemed quite random and unnecessary to then do it [get a urine sample]…the link wasn't really explained to me what the need for the sample was…so I tried, but yeah, it wasn't really clear as to what we were looking for in the sense of what she'd seen and why she felt she needed one [a sample]. [Int 266, parent]<br><br>They said that it [urine sample] would be worth having…because all of the other ones [tests] came back alright. It was one of those extra things to check. [Int 519, parent]<br><br>***Information about the 'clean catch' and other methods***<br>They just gave me the containers and said to send it back when I had collected it. [Int 881, parent]<br><br>In your cubicle [in hospital], they didn't give any specific instructions on how to collect. So they gave me the pot and said just make him pee here. And that was pretty much all the instructions that I received. [Int 479, parent]<br><br>No, I wasn't actually [told how to use the bag at home]. I think I looked it up online just to check. [Int 009, parent]<br><br>I've been told you know, be careful that doesn't get contaminated [urine container] but how particularly I do that I don't know…I've ended up following them around with Tupperware pots that I wiped with like sterilising wipes first…I mean I doubt it's that clean…I take their nappy off… but I didn't sort of do anything in terms of cleaning around their bottoms or anything. [Int 600, parent]<br><br>***Giving parents instructions***<br>I'm probably not very good at it actually. I probably say we're gonna need a urine and give them the collection pot and show them sort of how it works and just sort of say…it's a case of sitting there and waiting until they pee…I think I mean, you could blame time, but I think that's an excuse, not a reason…it's something we're doing all the time and they're not. [Int 998, paediatric nurse, secondary care]<br><br>***The need for policies around urine collection to help parents***<br>The amount of times people are set up for urine collections, there is a policy within the trust about urine collection, but I think it should be more specific about methods…..(When I) went up to the acute admissions unit, the nursing staff up there had never really seen it [a urine collection in pre-continent children] done, even their senior ones. So that information [about collection methods], I think sometimes gets lost unless you put it within a policy…I didn't think that they're [junior doctors] given enough education, and I certainly there's not a standard for it. [Interview 562, senior nurse, children's ED and acute ward]<br><br>I don't think some of them, specially when the junior doctors come through, they don't necessarily recognize how difficult it is for families to collect urines to make sure that they do definitely need them rather than arbitrarily saying, oh, I need a urine, yes, but that might take 2 hours and do you really need it for your clinical decision making? Is there an option to let the family go home and do it, you know? [Interview 562, senior nurse, children's ED and acute ward]
Information for parents	***Providing better information about urine collection for parents***<br>I think a leaflet is fine. Especially if there's like a diagram or something. Yeah, if a doctor wants to take the time to explain that as well, that's helpful…and then if you have questions,

For peer review only - http://bmjopen.bmj.com/site/about/guidelines.xhtml

	obviously, you can ask them…What happens a lot is that people assume…that you've been told stuff already. [Int 507, parent]<br><br>It would be something that had fairly clear instructions for the parent, and that were sort of easy to convey fairly quickly too. Because, obviously, there's always the time pressures in general practice. [Int 201, GP]<br><br>We were actually thinking about doing a bit of a quality improvement project in our department…making some videos on common procedures…that the parents could watch. So that was a way to potentially think about making it a bit more consistent as to what is explained. [Int 980, advanced nurse practitioner, children's ED]<br><br>***Information about 'acceleration' methods***<br>So the first and most definite, if the kid doesn't drink then nothing is going to come out. [Int 285, staff nurse, secondary care]<br><br>But the problem is with the tap method, why it's not used as much as because it takes three people to do it. [Int 562, senior nurse, children's ED and acute ward]<br><br>I think putting his feet on the floor because it was cold made him wee. I tried blowing as well 'cause that's, you know, as soon as you take a boys nappy off they usually wee… [Int 451, parent]
Towards a new device design	***Desired characteristics of a new device***<br>I think the other thing that really struck me was the shape of the sample pot. Seemed really non ideal for I mean, if it had been more like a funnel or something like that, that [could] have managed to catch a stream of urine in a really narrow pot. [Int 869, parent]<br><br>Something like the urine bag, but that is easier to attach and to detach, because he has been stressed when we have tried taken it off. [Int 881, parent]<br><br>I think ideally, there would be something that you can just take the baby out of their nappy and put on another nappy and wait for them to wee and then have got a sterile sample…So I think we're removing the kind of human element of having to watch and wait and time to catch it, that would be the best thing. [Int 298, paediatric surgeon, secondary care]<br><br>I think maybe that whatever item that might be designed, it needs to sort of be easy to use that any and everyone can like learn how to use it fairly quickly because obviously there are a lot of GPs in the practice. [Int 570, GP]<br><br>Our trust would never pay I don't think for expensive kit. If it can be done cheaply, then that's the way to do it. [Int 767, consultant, children's ED]<br><br>It's just something simple, something that the parents or the staff can easily use. And then I suppose easily disposed of as well. [Int 630, paediatric urology nurse, secondary care]

For peer review only - http://bmjopen.bmj.com/site/about/guidelines.xhtml

Declarations

Ethics approval and consent to participate

Ethical approval was granted by the Central University Research Ethics Committee, University of Oxford (R77332/RE002). Participants received written information about the study and had the opportunity to ask questions. Participants gave written consent before interviewing took place.

Availability of data and materials

The datasets generated and analysed during the current study are not publicly available because participant consent to share the original data outside the research team was not sought but reasonable requests for further information which does not compromise participant anonymity and confidentiality may be obtained from the corresponding author.

Competing interests

The authors declare that they have no competing interests.

Funding

This work was supported by the University of Oxford (Grant number: N/A) and the National Institute for Health Research (NIHR) (Grant number: MIC-2016–018).

Acknowledgements

We would like to thank all the parents and clinicians who took part in interviews as well as the NIHR Community Healthcare MIC's Patient and Public Involvement and Engagement (PPIE) for children and young people's health for their support for our study.

Authors' contributions

MAr, JB, GH, CB and MG were involved in the design of the study. MAr and MAb recruited participants, collected all the data and led analysis of the data, supervised by MG and GH. All authors were involved in interpretation of the analysed data and contributed to the final manuscript. All authors read and approved the final manuscript.

Word count

3531 words

For peer review only - http://bmjopen.bmj.com/site/about/guidelines.xhtml

For peer review only

For peer review only - http://bmjopen.bmj.com/site/about/guidelines.xhtml

SECTION A. RESEARCH DETAILS	
**1. Full title of research**	Exploring perspectives, priorities, and solutions of urine collection: A qualitative user centric study to understand practices and gain feedback from healthcare professionals and parents/carers
**2. Short title of research**	Urine collection qualitative studies
**3. Principal Investigator (PI) / Student Supervisor**	Dr. Jeroen Bergmann
**4. PI's training in research ethics and/or GCP**	"Children and Clinical Research" from the Global Health Training Centre, Course completed on 31/08/2020 JB<br>"ICH Good Clinical Practice (GCP) E6", from the Global Health Training Centre, Course completed on 31/08/2020 JB<br>"Research Integrity", University of Oxford, Course completed on 31/08/2020 JB<br>"Research Integrity in Engineering and Technology", University of Oxford, Course completed on 26/02/2015 JB<br>"Introduction to Research Ethics" from the Global Health Training Centre, Course completed on 19/01/2015 JB<br>"Human Research", Basic Course, Collaborative Institutional Training Initiative, MIT, Completed on 12/11/2013 JB
**5. Student name and degree programme (if applicable)**	
**6. Department/Institute name**	Department of Engineering Science
**7. University email address**	jeroen.bergmann@eng.ox.ac.uk
**8. University telephone number**	
**9. Funding Source**	EPSRC IAA / NIHR BRC
**10. State any conflicts of interest and explain how these will be addressed**	None

For peer review only - http://bmjopen.bmj.com/site/about/guidelines.xhtml

SECTION B. RESEARCHERS	
**1. Researcher title and name**	Dr. Monica Armengol
**2. Department / Institute name**	Department of Engineering Science
**3. Role in research**	Researcher will obtain informed consent and conduct research activities with participant
**4. Training in research ethics and/or research integrity**	"Children and Clinical Research" from the Global Health Training Centre, Course completed on 23/06/2021 MA<br>"Research Integrity", University of Oxford, Course completed on 24/06/2021 MA
**5. Researcher title and name**	Dr. Christopher Bird
**6. Department / Institute name**	MIC Oxford Health NHS Foundation Trust
**7. Role in research**	Design and supervision of project, liaise with medical professionals
**8. Training in research ethics and/or research integrity**	GCP up to date July/2021 (Health Research Authority, the governing body for NHS Ethics in the UK)
**9. Researcher title and name**	Dr. Gail Hayward
**10. Department / Institute name**	Primary Care Health Science
**11. Role in research**	Design and supervision of project
**12. Training in research ethics and/or research integrity**	GCP, 2019 (Health Research Authority, the governing body for NHS Ethics in the UK)
**13. Researcher title and name**	Dr. Philip Turner
**14. Department / Institute name**	Primary Health Care Science
**15. Role in research**	Design and supervision of project
**16. Training in research ethics and/or research integrity**	"Research Integrity", University of Oxford, Course completed on 21/09/2021
**17. Researcher title and name**	Nathaniel Mills
**18. Department / Institute name**	NIHR Children & Young People MedTech Cooperative

For peer review only - http://bmjopen.bmj.com/site/about/guidelines.xhtml

**19. Role in research**	Support and supervision. Liaise to medical professionals
**20. Training in research ethics and/or research integrity**	NIHR Good Clinical Practice course
**21. Researcher title and name**	Dr. Paul Dimitri
**22. Department / Institute name**	NIHR Children & Young People MedTech Cooperative
**23. Role in research**	Support and supervision. Liaise to medical professionals
**24. Training in research ethics and/or research integrity**	GCP (Health Research Authority, the governing body for NHS Ethics in the UK)<br>GCP Paediatrics Module (Health Research Authority, the governing body for NHS Ethics in the UK)
**25. Researcher title and name**	Gemma Wheler
**26. Department / Institute name**	NIHR Children & Young People MedTech Cooperative
**27. Role in research**	Design of study and analysis support
**28. Training in research ethics and/or research integrity**	NIHR Good Clinical Practice course
**29. Researcher title and name**	Molly Abbott
**30. Department / Institute name**	Oxford Health NHS Foundation Trust and University of Oxford
**31. Role in research**	Recruitment of Interviewees and performing interviews
**32. Training in research ethics and/or research integrity**	Oxford GCP training course for clinical researchers 28 March 2021

SECTION C. BASIC INFORMATION

**1. Provide a brief lay summary of the aims and objectives of the research. This should cover the questions it will answer and any potential benefits.**<br>**(Maximum 300 words)**	Urine is used to diagnose and monitor different diseases including diabetes and urine tract infections (UTI). Paediatric UTI accounts for 14% of the emergency department visits. Despite the high occurrence, collection of urine samples in non-toilet trained infants is challenging, lengthy and up to 30% of urine samples are contaminated with bacteria from the back passage or skin, making results meaningless.<br><br>Re-testing urine samples due to contamination causes delays, increases visits to the GP and emergency services. Additionally, it is associated with misdiagnosis, which can cause bacterial resistance if children are prescribed an antibiotic due to erroneous diagnosis.<br><br>This study aims to understand the current practices of urine collection in non-toilet trained infants and the challenges faced when carrying out, ordering or waiting for a collection.

For peer review only - http://bmjopen.bmj.com/site/about/guidelines.xhtml

	These findings will guide the focus areas for the development of a new device for paediatric urine collection, requirements, and specifications of the device.<br><br>We believe that a user-centric approach in the development of medical devices, produces device of higher clinical value. Participants will provide input in two parts: (1) interviews will aim to explore practices, costs, experiences, and perceptions of available diagnostic technology used for urine collection in infants in clinical settings. Additionally, we will explore unmet needs with regards to urine collection technologies and the potential impact of new diagnostic methods, tools or technology.<br>(2) a co-design workshop will allow participants to have input into possible solutions to the urine collection issues reported during the interviews.
**2. List all places where research will be conducted (including any other countries and online)**	Most interviews and workshop will be conducted online, via teams/zoom. Interviews will be conducted out of NHS premises.
**3. Anticipated research start date**	As soon as ethics is approved. Oct-Nov 2021
**4. Anticipated research end date**<br><br>(n.b. A maximum of 5 years approval can be granted)	3 years after approval
**5. Please list any CUREC Approved Procedure(s) you will follow**	
**6. Please list any CUREC Best Practice Guidance used to develop your research**	BPG 01 Researcher Safety/ BPG 03 Elite and expert interviewing/ BPG 09 Data collection, protection and management/ BPG 10 Conducting research interviews
**7. Name of departmental / peer reviewer (if applicable)**	Engineering Science / Sarah Hoeksma

**SECTION D. PARTICIPANTS**	
**1. Age range of participants**	Between 18 and 70 years old
**2. Are research participants classed as people whose ability to give free and informed consent is in question?**	Yes
**3. Anticipated number of participants**	Around 30 healthcare professionals and 20-30 parents

For peer review only - http://bmjopen.bmj.com/site/about/guidelines.xhtml

**4. How was the number of participants decided?**	We anticipate conducting between 20-30 interviews to healthcare professionals and 20-30 interviews to parents/carers. However, recruitment will continue until data saturation is reached or sufficient exploratory power for the findings is achieved. Further interviews might be required to fully explore the topics proposed. The research team will decide on concluding interviews.<br><br>For the workshop, we anticipate around 30 people taking part. This will include parents and healthcare professionals that took part in the interviews, in addition to other parents, health care professionals, engineers, designers and general public that are interested in taking part.	
**5. Inclusion criteria**	Healthcare Professionals<br><br>Consenting adults with experience working with urine collection whether direct or indirect (perform, assist, manage, purchase, order or interpret) within a clinical setting. These include medical professionals, nurses, administrative staff and bioanalyst.<br><br>Parents/Carers:<br><br>Consenting adults with experience aiding a pre-continent child collect a urine sample in the last 2 years.<br><br>The adults taking part in the interviews, both parents/carers and healthcare professionals, must be able to take part on the interview conducted in English and consent to the interview being recorded. Participants must be over the age of 18 years and below 70 years old.<br><br>Co-design workshop<br><br>Consenting adults over the age of 18 years and below 70 years old with an interest in urine collection in infants, including those that took part in the interviews. Additionally, engineers, designers, industry experts and academics will be invited.	
**6. Exclusion criteria**	The participant may not enter the study if they are unable to take part in an interview in English or written consent cannot be provided.	
**7. Please mark 'X' against all planned recruitment methods**<br><br>Provide copies of all recruitment material for review	Poster advert	☒
	Flyer	☒
	Email circulation	☒
	In-person approach	☒
	Website	☒
	Social media (e.g. twitter, Facebook)	☒
	Snowball sampling (recruiting through contacts of existing participants)	☒
	Newspapers	☐
	Research recruitment sites (e.g. Prolific Academic, Amazon Turk)	☐
	Existing departmental contacts or volunteer database	☒
	Other (please specify)	☐
**8. How will potential participants be identified and approached?**	Information about the study and poster will be shared in social media (including Facebook groups [with permission of the group moderator] i.e. Community and parents groups, for example [Oxford] Community, [Oxford] Parents & Careers, paediatric organisations, charities and research groups), twitter (personal	

For peer review only - http://bmjopen.bmj.com/site/about/guidelines.xhtml

	accounts, research group account and organisations we are collaborating with – NIHR Children and Young People (CYP) Medical Technology and In-vitro Co-operative (MIC)/ NIHR Community Health (CH) MIC), blogs.<br><br>Newsletters and mailing lists could be used to share information and poster. Poster will include detail of £20 remuneration in the form of electronic gift vouchers will be offered for time taken to participate in interview.<br><br>These will include the University Department's mailing list charities and baby groups, paediatric medical organisations and, partners from the NIHR MICs will share information in their website and newsletter. The emails will be sent on behalf of the researchers conducting this study. We will also publish information of the study in our website and rely on word of mouth of participants.<br><br>Participants can express their interest in taking part of the study by contacting the researcher by email or phone. We will also include a form on the website for people to express their interest. Any potential participant expressing interest in taking part in the study will be contacted by email or telephone. Potential participants will be directed to our website where they can download the participant information sheet and consent form. Participants wishing to take part will be asked to return the consent form and participant information sheet signed. Participants wishing to take part, will be invited (by email or phone) to take part in the co-design workshop. Information and invite in the co-design workshop will use the same medium as the interviews did for distribution.<br><br>Participants will be given options regarding the timing, format, and venue (if face-to-face) for the interview will be given. The participant will have the opportunity to ask any questions either by email or telephone. They may choose to decline any further contact; in which case they will be reassured that this will in no way impact upon their current or future working.<br><br>The workshop will have a set date, participants will be asked to register their attendance in advance.
**9. Will informed consent be obtained from the research participants or their parents/ guardians?** If not, please explain why not.	Verbal consent will be taken at the start of interviews and co-design workshop. Before the interview/workshop starts, we will explain the study to the participant and go through the consent form.<br><br>Written informed consent will be obtained by means of participant dated signature and dated signature of the person who presented and obtained the Informed Consent.<br><br>Written consent will be sought participants will be given the option to fill the consent themselves and emailing it to us, they can also complete it through a GDPR compliant platform (https://www.smartsurvey.co.uk/) or researcher can fill them on their behalf and email a password-protected copy and retaining a copy for the records. For interviews being done by phone, form will be sent by email, or if not possible, oral consent will be sought.<br><br>Participant will be able to ask any questions or withdraw at any point without any explanation.
**10. For each activity or group of participants, explain how informed consent will be obtained from the participants themselves and/or their**	Verbal consent will be taken at the start of the interview and workshop. Study will be explained and the researcher will go through the consent form with the participant.<br><br>Written consent will be sought prior to interviews and co-design workshop are taking place. As interview/workshop will be conducted remotely (either online or

For peer review only - http://bmjopen.bmj.com/site/about/guidelines.xhtml

**parents/guardians, if applicable. How will their consent be recorded?**	by phone) participants will be given a few options to fill the consent form; the option to fill the consent themselves and emailing it to us, they can fill it online through a GDPR compliant software (https://www.smartsurvey.co.uk/) or researcher can fill them on their behalf and email a password-protected copy and retaining a copy for the records. A copy of the consent form will be made and stored safely in password protected file.<br><br>Participants will be given the opportunity to ask questions or withdraw from study. If participants are happy to proceed, interviews will be arranged online or by telephone at their convenience. A date will be set for the workshop, participants can register their interest to participants through the website. We will be available to reply to any questions or concerns that the participant may have at any point during this process.

SECTION E. RESEARCH METHODOLOGY

1. Please mark 'X' against the methods that will be used in your research

Ensure you address each method you will use in your informed consent documents and on this form

Use of casual or local workers (e.g. interpreters)	☐	Audio recording of participant	☒
Interview (refer to guidance in BPG 10: Conducting research interviews)	☒	Video recording of participant	☐
Focus group	☐	Photography of participant	☐
Participant completes questionnaire in hard copy	☐	Physiological recording from participant	☐
Participant completes online questionnaire or other online task (refer to guidance in BPG 06: Internet-mediated research)	☐	Taking a sample of blood or other bodily fluid from a participant	☐
Use of social media to recruit or interact with participants (refer to guidance in BPG 06: Internet-mediated research)	☒	Participant observation	☐
Analysis of existing records	☐	Covert observation	☐
Participant performs verbal or aural task	☒	Systematic observation	☐
Participant performs paper and pencil task	☒	Observation of specific organisational practices	☐
Participant performs computer based task	☒	Other (please specify below)	☐
Measurement/recording of motor behaviour	☐		

2. Provide a lay description of the research design and methods. In particular, describe clearly what participants in the research will be asked to do.

Interviews:

Before interview starting, the information sheet will be reviewed, and any questions addressed.

- Participants will be asked to provide their informed consent to participation prior to data collection. Participants will be explained to that their participation is voluntary and they can end or withdraw from study at any point without any explanation.

For peer review only - http://bmjopen.bmj.com/site/about/guidelines.xhtml

- A date for the interview to take place will be scheduled. Participants will have a choice of date, time and place of interview (online or telephone).
- Sessions will last between 40-60 min. Each participant will have one session. Consent will be asked in case follow-up is required in any of the questions.
- A qualitative methodology will be used given that it is appropriate to explore people's knowledge, choices, and decision. Data collection and analysis will be guided by the principles of grounded theory which allows adjustments to questions and sampling.
- Semi-structured questionnaires with open-ended questions will be used for the interviews. A topic guide will be used to conduct the interviews. This will allow flexibility in the order and wording of the questions. Additionally, questions will be amended based on on-going interviews, to explore emerging ideas and themes and to include new participants as participants for interview (theoretical sampling).

The topic guide will be divided into three parts, a warmup section that will include personal information and questions to get to know the person being interviewed and their relationship to urine collection. Then the main questions section, which will determine experiences, stories and opinions about urine collection methods. Finally, the wrap-up questions, to give the opportunity to ask any questions to us.

The open-ended questions will be used to probe the different areas of urine collection we are interested in. The interviews will explore the procedure, i.e., who uses it, device used, procedure (who collects the urine, how long it takes, final sample), post-collection feedback and process, patient follow-up.

- Interviews will be audio recorded and transcribed. If only audio is not possible (during online meeting),participant will be asked to turn off their video off during the interview..

Co-design Workshop

- Participants will be invited to take part in the workshop. They can express their interest by filling a registration form on the website.
- Participant will be able to download the consent and participant information form from the website. And will be able to contact the researchers with questions using the contact information provided in the participant information sheet.
- Participants will be asked to provide their informed consent to participation before the workshop starts. Participants will be explained to that their participation is voluntary and they can end or withdraw from study at any point without any explanation. They will also have the opportunity to ask questions.
- Participants will be divided in groups, where they can discuss urine collection ideas and brainstorm new solutions. This can be by drawing/writing/verbal explanation or making something with available materials. At the end each group will be able to present the summary of their ideas to the rest of the participants and organisers.
- If workshops are held online. An online worksheet will be set up and teams will work on their ideas there. Teams will be asked to present their ideas at the end of the workshop.
- Notes will be taken and online worksheets will be kept by the research team for analysis and further work. No video or audio recording will be taken.
- Session will last between 2.5-3h

For peer review only - http://bmjopen.bmj.com/site/about/guidelines.xhtml

**3. Will the research include any audio, video or photographic recordings?**
Yes. Participants who don't wish to be recorded could still take part. In this case researcher will take notes during the interviews.
**4. Biological sample handling**
No biological samples will be taken as part of the study.
**5. Please detail all expenses or gifts that will be offered to participants.** Guidance is available in Best Practice Guidance: 05 Payments and incentives in research.
R~~No r~~emuneration will be given for participation in the study, in the form of £20 electronic gift vouchers for time taken to participate in interviews.~~.~~

SECTION F. ETHICAL CONSIDERATIONS

For guidance on ethical issues, please see http://researchsupport.admin.ox.ac.uk/governance/ethics/resources

(N.B. To complete, double click on the check boxes and select 'checked')

**1. Will the research involve any participants considered vulnerable in the context of the research (e.g. children, elderly, prisoners)?** **If yes,** please describe how they are defined as vulnerable and detail any CUREC Approved Procedures or guidance that will be applied to the research (for current documents and templates see https://researchsupport.admin.ox.ac.uk/governance/ethics/resources). **If yes, and you cannot apply any Approved Procedure**, please cease completion of this form – a CUREC 2 application is required	Yes ☐	No ☒
**2. Will unequal relationships exist between participants and those obtaining informed consent?** **If yes,** describe the nature of the unequal relationship and how arising ethical issues will be addressed	Yes ☐	No ☒
**3. Will the research involve questions and/or discussions of contentious and/or sensitive issues (e.g. information relating to ethnicity, political opinions, religious beliefs, physical/mental health or sexual life)?** **If yes,** please justify why this is required and provide a copy of the questionnaire raising the issues that will be used in your research.	Yes ☒	No ☐

Healthcare professionals:

We know urine collection methods and procedure are not consistent throughout different clinical practices or clinicians. We would like to understand what drives the decision making. We know different countries and culture

For peer review only - http://bmjopen.bmj.com/site/about/guidelines.xhtml

have different acceptability to invasive or non-invasive procedures, so would like to test whether ethnicity plays a role.

Parents:

We would like our study to be diverse and include people from different social-economic backgrounds and ethnic backgrounds. In order to quantify the success rate of these goals, and report them, we will ask parents/carers to state their ethnicity and questions that will allow us to estimate their social-economic position.

Workshop:

In order to be able to report who took part in this study, we will ask demographic questions. Participants will have the chance of not answering if they don't feel comfortable doing so.

**4. Will the research involve deliberate deception of participants outside the scope of CUREC Approved Procedure 07?**<br>**If yes**, please cease completion of this form – a CUREC 2 application is required<br>**If no,** please either give details of the deception or state that no deception will be involved	Yes ☐	No ☒
**5. Could the proposed research affect your own physical and/or psychological safety as a researcher?**<br>**If yes,** describe how you will manage this.	Yes ☐	No ☒

6. How will you ensure the research is conducted according to the details given in this form?

Members of the research team will meet weekly to discuss progress or issues and themes that are arising from the interviews in order to make decision about the continuity and direction of interviews. Moreover, these discussions will also support the data analysis. And if needed, another person will be review recordings to validate findings.

Any students involved in the study will form part of the research team and will present findings on weekly basis. Students will be supported in their analysis by senior researchers.

Procedures will be discussed during meetings. We have involved people with experience in qualitative studies, who will support the process. Additionally, senior members of the research team will be able to join interviews and analysis, in order to check procedures are followed.

If any participant has questions, they will be able to ask the research team directly. They will also be able to reach the principal investigator if they feel appropriate. We will acknowledge reception and will give an indication of how it will be dealt with. Any serious complaints will be forwarded to the Chair of the Research Ethics Committee at the University of Oxford (ethics@medsci.ox.ac.uk).

7. Please give details of any other research-specific ethical and/or safety considerations

None

8. How do you propose to deal with / handle any incidental findings?

Not applicable

For peer review only - http://bmjopen.bmj.com/site/about/guidelines.xhtml

**9. Will any data or information from this study be provided to individual participants?**
No. The study aims to explore and validate challenges faced during urine collection. Therefore, there are no individual results to be provided.

**SECTION G. OTHER CONSIDERATIONS**		
**1. Is any part of this research being conducted overseas?**<br>**If yes,** please give details below. Ensure you complete and submit a travel risk assessment form to your departmental safety officer. (This is necessary to ensure the travel/fieldwork is covered by the University's travel insurance – see http://www.admin.ox.ac.uk/finance/insurance/travel/)	Yes ☐	No ☒
**2. Does your research raise issues relevant to the Counter-Terrorism and Security Act (the Prevent Duty), which seeks to prevent people from being drawn into terrorism?**<br>**If yes,** please say how you plan to address any related risks. Please see advice on this on our Best Practice Guidance Web Page.	Yes ☐	No ☒

SECTION H. DATA MANAGEMENT AND HANDLING

All information provided by participants is considered **research data** for the purpose of this form. Any research data from which participants can be identified is known as **personal data**; any personal data which is sensitive is considered **special category data**.

Management of personal data, either directly or via a third party, must comply with the requirements of the General Data Protection Regulation (GDPR) and the Data Protection Act 2018, as set out in the University's Guidance on Data Protection and Research.

In answering the questions below, please also consider the points raised in the Data Protection Checklist and whether, for higher-risk data processing, a separate Data Protection Impact Assessment may also be required for the research. Advice on research data management and security is available from Research Data Oxford and your local IT department. Advice on data protection is available from the Information Compliance team.

1. Please mark 'X' against the data you will collect for your research

Screening documents	☐	Audio recordings	☒
Consent records including participant name or other identifiers (e.g. written consent forms, audio-recorded consent, assent forms)	☒	Video recordings	☐

For peer review only - http://bmjopen.bmj.com/site/about/guidelines.xhtml

Consent obtained anonymously (e.g. via online survey)	☐	Transcript of audio/video recordings	☒
Opt-out forms	☐	Photographs	☐
Contact details for the purpose of this research only	☒	Information about the health of the participant (including mental health)	☐
Contact details for future use (guidance)	☒	Physiological test results / measurements	☐
Field notes	☒	MRI scans	☐
Task results (e.g. questionnaires, diary completion)	☐	IP addresses (refer to Best Practice Guidance 09: Data collection, protection and management for guidance)	
Data already in the public domain.<br>Specify the source of the data:	☐	Other (please specify below)	☐
Previously collected (secondary) data	☐		

2. How and where will each type of data be stored whilst the research is ongoing (until the end of all participant involvement)?

List each type of data selected above, and explain how each will be physically transferred (including movement/sharing of audio files, paper records, electronic downloads etc.) from where it is collected to a suitable storage site (e.g. Nexus365 OneDrive for Business, SharePoint, University servers). State the storage location for each. Do not store unencrypted data in freely available cloud services or unprotected USB drives.

Refer to Best Practice Guidance on data collection, protection and management (BPG09).

Hard copies of data (consent forms and field notes) will be placed in a folder and transferred to an office at the Engineering Science Department as soon as possible. In the Engineering Science Department, consent forms will be stored in a locked filling cabinet. When, despite efforts, it is not practical to immediately store data in this location, a lockable filling cabinet in the researcher's home will be used and will be transferred to the Engineering Science Department as soon as possible.

All electronic data (typed field notes, audio recordings and personal data including contact details) will be transferred from the researcher's password protected device to a password-protected drive within the University network. The audio recording held by the researchers will then be deleted once the interview audio has been transcribed. These recordings will be securely transferred using the university file sharing system or Nexus OneDrive for business. Workshops work documents will be de-identified.

Identifying information from audio recordings will be removed from the transcripts. Transcription will be done by the researcher or approved University transcriber. De-identified transcripts may be uploaded into NVIVO 12 which will accessed by the research team on password-protected computers. This de-identified data may also be made available to other researchers.

The transcription will be stored as a Word file on encrypted computers within the University network.

3. Will you use a unique participant number on research data instead of participant name?

If yes, state whether or not you will retain a list of participant names against numbers (pseudonymisation via a linkage list).

Where will the list be stored, and when will it be destroyed?

Yes. Data identifiers will be labelled and stored securely and separately from the research data, on password protected computers. We will destroy participants information once research has been completed and published.

For peer review only - http://bmjopen.bmj.com/site/about/guidelines.xhtml

4. Who will have access to the research data?

Researchers listed on this form will have access to the research data and other student/researchers working on the study. Access will be granted to the MS IDREC for the purposes of monitoring and/or audit of the research.

De-identified data may be made available to other researchers.

5. If research data is to be shared with another organisation, how will it be transferred / disclosed securely?

Any data will be shared securely using the university file sharing system (OxFile or Nexus365 OneDrive Business system) and returned the same way. The data will be stored in password-protected computers within their institutions.

6. When and how will identifiable data be destroyed or deleted?

N.B. If any identifiable data will be retained beyond the end of the study and/or indefinitely, please state what data this is, and the reasons for retention (e.g. contact details for future studies; photos used in publication). This must be clearly stated on participant information, and specific consent obtained.

All identifiable data will be removed from the transcript.

Unique participants number will be used. Audio recordings will be destroyed after publication.

Consent records and de-identified transcripts and worksheets will be kept 3 years after publication.

Personal data will be deleted from servers after the analysis and publication has been completed.

**7. Please confirm that you will store other (non-identifiable) research data safely for at least 3 years after final publication or public release and adhere to any additional research funder policies.**<br>For more information about the University policies, please see the University's web pages on research data management.<br>**If 'Yes'**, please give details of who will store the data and on storage format, location and security.<br>**If 'No'**, please provide further details.	Yes ☒	No ☐

Non-identifiable data will be stored in a password-protected computer within the University network at the PI's department.

SECTION I. PUBLICATION AND DISSEMINATION OF RESULTS

**1. How will you disseminate and feedback project outcomes at the end of the research?**	Study finding will be disseminated though publications in peer-reviewed journals, conferences and other articles such as for the Community Health MIC, Children and Young People MIC. Public journals or seminars.

For peer review only - http://bmjopen.bmj.com/site/about/guidelines.xhtml

	We will aim to make these findings available to the scientific and wider community by following the Open Science guidelines.

For peer review only

For peer review only - http://bmjopen.bmj.com/site/about/guidelines.xhtml

DECLARATIONS AND SIGNATURES

In providing signatures, the MS IDREC Secretariat will accept either:

Option 1: Email confirmations sent from a University of Oxford email address. Separate emails should be sent by each of the relevant signatories as outlined below, indicating acceptance of their responsibilities.

Option 2: That the form be fully-signed with handwritten (wet-ink) signatures. Please scan these and the rest of the form pages to create a single PDF document and email to us.

PRINCIPAL INVESTIGATOR (AND STUDENT IF APPLICABLE)

I/We, the researcher(s):

- Understand our responsibilities as outlined on this form and in the CUREC glossary and guidance
- Agree to start this research only after obtaining approval from the MS IDREC;
- Understand that the Principal Investigator must ensure that all researchers are suitably qualified and trained to conduct the research described, or are appropriately supervised until deemed qualified/trained;
- Agree to provide additional information as requested by the MS IDREC before approval is secured and as research progresses;
- Agree to maintain the confidentiality of all data collected from or about participants;
- Agree to notify the MS IDREC in writing immediately of any proposed change to the research, and await approval before proceeding with the proposed change;
- Agree to notify the MS IDREC if the Principal Investigator changes and supply the name of the successor;
- Will use the data collected only for the research for which approval has been given;
- Will grant access to data only to authorised persons; and
- Have made arrangements to ensure that personal data collected from participants will be held in compliance with the requirements of the GDPR and the Data Protection Act 2018.

**Principal Investigator (Name)**	Jeroen Bergmann
**Principal Investigator (Signature)** Pasted images of signatures cannot be accepted	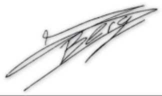
**Date**	30/06/2021
**Student (Name)**	
**Student (Signature)** Pasted images of signatures cannot be accepted	
**Date**	

For peer review only - http://bmjopen.bmj.com/site/about/guidelines.xhtml

ACCEPTANCE BY HEAD OF DEPARTMENT/FACULTY OR DESIGNATED NOMINEE*

*Another senior member of the department may sign where the head of department is the Principal Investigator, or where the head of department has appointed a nominee. Example nominees include Deputy Head of Department, Director of Research, or Director of Graduate/Undergraduate Studies.

I have read the research proposal above. On the basis of the information available to me, I:

- consider the Principal Investigator/Supervisor and student researcher (if applicable) to be aware of their ethical responsibilities in regard to this research;
- am satisfied that the proposed design and scientific methodology are sound; the research has been subject to appropriate peer review and is likely to contribute to existing knowledge and/or to the education and training of the researcher(s) and that it is in the public interest.

For peer review only

**Head of Department or designated nominee (Name)**	
**Head of Department or designated nominee (Signature)**<br>Wet-ink signature (not pasted electronic image)<br>*or*<br>The Head of Department/nominee can send an email (including PI name and study title) to ethics@medsci.ox.ac.uk confirming the above	
**Date**	

For peer review only - http://bmjopen.bmj.com/site/about/guidelines.xhtml

Study Title: Exploring perspectives, priorities, and solutions of urine collection: A qualitative user centric study to understand practices and gain feedback on from healthcare professionals and parents/carers
Document Title: Topic Guide Healthcare Staff
Date and Version Number: 20-Oct-2021, V0.1
CUREC Reference Number: R77332/RE001

Interview questions

This is a semi-structured interview. The questions below provide a guide and are not fixed. The interviewee can talk freely during the interview and the conversation will guide which questions will be asked next. The interview has 3 stages: warm-up questions, main questions, and wrap-up questions.

Introduction

Reassure interviewee that this is an interview to understand practices and knowledge around urine collection in order to design new solutions including technology, rather than judging or evaluating practices of individual.

Healthcare Professionals:

Warm-up questions

Interviewees will be asked to complete these questions in writing. Demographic questions will have an option "prefer not to say". Interviewer will remind participants that they do not need to answer any question they don't wish to answer.

All Healthcare Professionals

- Tell us about yourself
  - which hospital do you work in?
  - what is your role?
  - how long you have been in the role?
- Demographic questions
  - Age
  - Gender
  - Ethnicity
- How did you hear about this study?

If Medical Practitioners/Nurses

- About position
  - Main location of practice
  - Country of medical or nursing degree if applicable
  - Years of working in as current specialty (ie. As GP / in ED / Admin)
  - Training level
  - Do you know how many children under 2 would have a urine collection a week?
  - Specialised in paediatrics
  - Additional postgraduate qualification in paediatrics or child health
- Number of full-time equivalent GPs at clinic/number of doctors in ED
- Number of full-time equivalent nurses
- Type of clinic (private, mixed, NHS, other)
- What is your role related to urine collection?

For peer review only - http://bmjopen.bmj.com/site/about/guidelines.xhtml

Main questions

If Medical Practitioners/Nurses

Deep dive into the urine collection process:

- In the hospital you work in / have worked in, is there a preference for a particular urine collection method?
- What is your experience carrying out urine collection in infants? Have you used all methods?
- How often do you perform urine collection in children? / (in a day/week how many urine samples are collected?)
- Think about a recent experience carrying out/ordering/supervising/assisting a urine collection in an infant
  - Age of patient
  - Physical and emotional condition of patient / parent
  - Where was the collection done? Home, hospital, GP practice?
  - Who ordered the urine collection?
  - Where did the collection take place?
  - How many people were involved in the procedure?
  - Who carried out the collection?
  - How long did it take?
  - Any feedback?
  - Follow-up of patient
- Tell me about how would you go about asking parents to obtain a urine sample from their child?
  - Instructions:
    - ~~Have you been taught how to give~~Do you provide urine collection instructions for parents?
      - If no: why not? (lack of time, lack of knowledge, don't think it's necessary)
      - If yes, what type of instructions do you provide?
        - Do you think this is normal practice with colleagues? (allude to the fact that most parents have mentioned not to get instructions, would they agree?.
          - Why do you think that is?
    - ~~Did you share this information with parents?~~
  - Procedure explanation
  - Why are you collecting the sample?
- Who decides to get a urine sample?
  - If you, when would you get a urine sample?. Is this process clear?
- What is your perception of non-invasive urine methods as a diagnostic procedure?

- What is your opinion about the urine collection devices available?
  - Invasive vs. non-invasive
  - What is the determining factor when choose a particular option?
- The NICE guidelines in UK suggest using the clean catch method. In your opinion is this the most common method used?
- Do you or your colleagues ever use voiding stimulating methods for clean catch (i.e. Quick-wee method, bladder-tapping method)?
  - If yes, what is the practice mostly used and your experience in terms of success rate, usefulness, difficulty
  - If not, why not?
- What do you think are the pros and cons of current urine collection devices/methods, if any?

Formatted

Formatted: Font: (Default) +Body (Calibri), 11 pt

Commented [MA1]: Here, trying to see, if they feel is an easy and valid method to diagnose an infection or if it's just a check list procedure.

Formatted: Font: (Default) +Body (Calibri), 11 pt

Formatted: List Paragraph, Bulleted + Level: 1 + Aligned at: 0.63 cm + Indent at: 1.64 cm

For peer review only

- Re-visits
- Misdiagnosis
- Contamination
- Usability

- Do you know of other novel urine collection devices?
- Having considered everything mentioned before,
  - what would the ideal process look like from beginning to end?
  - Where can/does the process go wrong?

<u>If Bio-analyst</u>

Deep dive into the urine collection processes:

- Tell me about the work you do in regard to urine analysis in infants?
- How many urine collections happen in a month?
- How reliable are urine samples of infants for diagnosis of infections?
  - Do you have information of the method used for urine collection?
    - If yes, what do you think about invasive and non-invasive methods of urine collection?
    - How does clean catch compare?
    - Do you think there are other practices that reduce contamination rates
- Is retesting a frequent occurrence?
  - How much do you think this cost?
  - In your opinion what drives the use of one method or another?
- Having considered everything mentioned before,
  - what does the ideal process look like from beginning to end?
  - Where can/does the process go wrong?

<u>If admin staff</u>

Deep dive into the urine collection processes:

- Tell me about your experiences with urine collection in infants?
- How many urine collections happen in a month?
- What is the cost?
  - What are the resources used for a urine collection in infants
    - Duration for a collection
    - Use of room
    - Staff
  - How does clean catch compare to other methods?
  - In your opinion what drives the use of one method or another?
- The NICE guidelines in UK suggest using the clean catch method. In your opinion is this the most common method used?
  - Have you ever heard of voiding stimulating methods for clean catch (i.e. Quick-wee method, bladder-lumbar method)?
    - If yes, what is the success rate?
- Are you aware of contamination rates in different urine collection methods?
  - How does this affect cost?
- Do you know of other novel urine collection devices?
- Having considered everything mentioned before,
  - what does the ideal process look like from beginning to end?
  - Where can/does the process go wrong?

Wrap-up questions

For peer review only

End with asking them if they have any questions, ensuring they are comfortable with everything that has been said, and whether they would be open to further interviews in the future:

- Are there any questions you think I should have asked? Is there anything you would like to add?
- Who/where else do you think it might be interesting for us to interview? - How come? (because of different dynamic, scale and scope of work etc).
- If I had any quick questions arise from reviewing my notes today, would it be possible to give you a quick call/email?
- Would you been open to further interviews from us in the future as we continue to develop this tool?

Do you have any questions from us, would you like to know more about our urine collection project?

For peer review only

For peer review only - http://bmjopen.bmj.com/site/about/guidelines.xhtml

Study Title: Exploring perspectives, priorities, and solutions of urine collection: A qualitative user centric study to understand practices and gain feedback on from healthcare professionals and parents/carers
Document Title: Topic Guide Parents/Carers
Date and Version Number: 20-Oct-2021, V0.1
CUREC Reference Number: R77332/RE001

DEPARTMENT OF ENGINEERING SCIENCE

UNIVERSITY OF OXFORD

Interview questions

This is a semi-structured interview. The questions below provide a guide and are not fixed. The interviewee can talk freely during the interview and the conversation will guide which questions will be asked next. The interview has 3 stages: warm-up questions, main questions, and wrap-up questions.

Reminder of how we will use the data:

> We will record the interview. This will be transcribed, and all identifiable information will be erased from transcription and stored safely within the Engineering Department. Recordings will be erased.

Introduction

Reassure interviewee that this is an interview to understand what works and what doesn't work of current urine collection methods so we can design technology that helps parents and clinicians.

Warm-up questions

We will give demographic questions to interviewee for them to fill. The option of prefer not to say will be given. Interviewer will remind participant that they do not need to answer any question they prefer not to answer.

- Tell us about yourself;
  - Who do you care for?
  - How many children do you have?
  - Demographic questions
    - Age
    - Gender
    - Ethnicity (give options from a list)
    - Social demographics:
      - Post code
      - How many people live in the house?
      - Employment status (full time / part time / stay at home parent / unemployed)
      - Number of dependents
    - Education level
  - How did you hear about this study?

Main questions

Deep dive into the urine collection process:

- Tell me about your experiences with urine collection in infants and toddlers?
  - When did it last happen?
  - Where did the urine collection occur? i.e. hospital, home, GP

For peer review only - http://bmjopen.bmj.com/site/about/guidelines.xhtml

- If you sought medical attention, what were the symptoms your child had that worried you?
- Tell me about how you were told to get the sample
  - How was the urine sample collected?
  - Were you given instructions? Do you remember them (the instructions you were given)? → Urine Collection Method
  - Who gave them to you?
  - Did someone explain why a urine sample was needed?
  - Was there any leaflet or other information that helped with the process?
    - If yes, do you remember any helpful information?
- How did you find the process of urine collection in your child?
  - What were the challenges?
  - What worked well?

- When did this last happen?
- Did this happen with only one or many of your children? Does it happen often?
- How often do you perform urine collection in children?
- Are you aware that there are different urine collection methods?
  - Have you previously used other methods?
  - If you used other ways of collecting urine, which method did you prefer?
    - Why?
  - Did you have any worries about some of the collection methods?

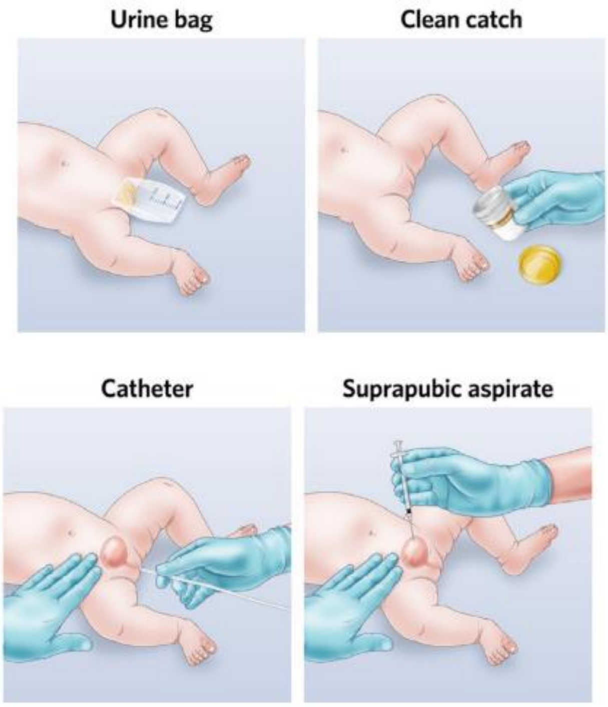

NON-INVASIVE

INVASIVE

Figure 1. Images by Dr. Jonathan Kaufman and Bill Reid, Royal Children's Hospital. Kaufman et al, BMJ, 2017. 357: 16-19.

- What is your opinion about the urine collection devices available?

For peer review only - http://bmjopen.bmj.com/site/about/guidelines.xhtml

  - Invasive vs. non-invasive, i.e. a sample requiring a nurse/doctor placing a urinary catheter versus getting the child to sit on a potty
  - If there was more than one option, were you given a choice?
- The NICE guidelines in UK suggest using the clean catch method. In your opinion was this the method used for your child.
  - Have you ever heard of ways to speed up collecting urine samples (eg gently tapping the bladder)?
    - If yes, did it work? / How long did it take?
- Are you aware that some urine samples are unreliable because they end being contaminated with bacteria (bugs) commonly found on our skin?
- Do you know of other urine collection devices?
- In your opinion:
  - what would the ideal process look like from beginning to end?
  - Where can/does the process go wrong?

Wrap-up questions

End with asking them if they have any questions, ensuring they are comfortable with everything that has been said, and whether they would be open to further interviews in the future:

- Would you like to add anything that we didn't ask about?
- Do you know anyone else that has gone through urine collection in a non-toilet trained infant that might be interested in participating?
- If I had any quick questions arise from reviewing my notes today, would it be possible to give you a quick call/email?
- Would you been open to further interviews from us in the future as we continue to develop this tool?

For peer review only - http://bmjopen.bmj.com/site/about/guidelines.xhtml

COREQ (COnsolidated criteria for REporting Qualitative research) Checklist

A checklist of items that should be included in reports of qualitative research. You must report the page number in your manuscript where you consider each of the items listed in this checklist. If you have not included this information, either revise your manuscript accordingly before submitting or note N/A.

Topic	Item No.	Guide Questions/Description	Reported on Page No.
**Domain 1: Research team and reflexivity**			
Personal characteristics			
Interviewer/facilitator	1	Which author/s conducted the interview or focus group?	
Credentials	2	What were the researcher's credentials? E.g. PhD, MD	
Occupation	3	What was their occupation at the time of the study?	
Gender	4	Was the researcher male or female?	
Experience and training	5	What experience or training did the researcher have?	
Relationship with participants			
Relationship established	6	Was a relationship established prior to study commencement?	
Participant knowledge of the interviewer	7	What did the participants know about the researcher? e.g. personal goals, reasons for doing the research	
Interviewer characteristics	8	What characteristics were reported about the inter viewer/facilitator? e.g. Bias, assumptions, reasons and interests in the research topic	
**Domain 2: Study design**			
Theoretical framework			
Methodological orientation and Theory	9	What methodological orientation was stated to underpin the study? e.g. grounded theory, discourse analysis, ethnography, phenomenology, content analysis	
Participant selection			
Sampling	10	How were participants selected? e.g. purposive, convenience, consecutive, snowball	
Method of approach	11	How were participants approached? e.g. face-to-face, telephone, mail, email	
Sample size	12	How many participants were in the study?	
Non-participation	13	How many people refused to participate or dropped out? Reasons?	
Setting			
Setting of data collection	14	Where was the data collected? e.g. home, clinic, workplace	
Presence of non-participants	15	Was anyone else present besides the participants and researchers?	
Description of sample	16	What are the important characteristics of the sample? e.g. demographic data, date	
Data collection			
Interview guide	17	Were questions, prompts, guides provided by the authors? Was it pilot tested?	
Repeat interviews	18	Were repeat inter views carried out? If yes, how many?	
Audio/visual recording	19	Did the research use audio or visual recording to collect the data?	
Field notes	20	Were field notes made during and/or after the inter view or focus group?	
Duration	21	What was the duration of the inter views or focus group?	
Data saturation	22	Was data saturation discussed?	
Transcripts returned	23	Were transcripts returned to participants for comment and/or	

For peer review only

Topic	Item No.	Guide Questions/Description	Reported on Page No.
		correction?	
**Domain 3: analysis and findings**			
Data analysis			
Number of data coders	24	How many data coders coded the data?	
Description of the coding tree	25	Did authors provide a description of the coding tree?	
Derivation of themes	26	Were themes identified in advance or derived from the data?	
Software	27	What software, if applicable, was used to manage the data?	
Participant checking	28	Did participants provide feedback on the findings?	
Reporting			
Quotations presented	29	Were participant quotations presented to illustrate the themes/findings? Was each quotation identified? e.g. participant number	
Data and findings consistent	30	Was there consistency between the data presented and the findings?	
Clarity of major themes	31	Were major themes clearly presented in the findings?	
Clarity of minor themes	32	Is there a description of diverse cases or discussion of minor themes?	

Developed from: Tong A, Sainsbury P, Craig J. Consolidated criteria for reporting qualitative research (COREQ): a 32-item checklist for interviews and focus groups. *International Journal for Quality in Health Care*. 2007. Volume 19, Number 6: pp. 349 – 357

Once you have completed this checklist, please save a copy and upload it as part of your submission. DO NOT include this checklist as part of the main manuscript document. It must be uploaded as a separate file.

For peer review only

BMJ Open

Clinician and parent views on urine collection in pre-continent children in the UK: a qualitative interview study

Journal:	*BMJ Open*
Manuscript ID	bmjopen-2023-081306.R2
Article Type:	Original research
Date Submitted by the Author:	28-Mar-2024
Complete List of Authors:	Armengol, Monica; University of Oxford, Department of Engineering Science<br>Hayward, Gail; University of Oxford, Nuffield Department of Primary Care Health Sciences<br>Abbott, Molly; Guy's and St Thomas' Hospitals NHS Trust<br>Bird, Chris; Birmingham Children's Hospital, Emergency Department<br>Bergmann, Jeroen; University of Oxford, Department of Engineering Science<br>Glogowska, Margaret; University of Oxford, Nuffield Department of Primary Care Health Sciences
**Primary Subject Heading**:	Paediatrics
Secondary Subject Heading:	Qualitative research
Keywords:	PAEDIATRICS, Urinary tract infections < UROLOGY, QUALITATIVE RESEARCH

SCHOLARONE™
Manuscripts

I, the Submitting Author has the right to grant and does grant on behalf of all authors of the Work (as defined in the below author licence), an exclusive licence and/or a non-exclusive licence for contributions from authors who are: i) UK Crown employees; ii) where BMJ has agreed a CC-BY licence shall apply, and/or iii) in accordance with the terms applicable for US Federal Government officers or employees acting as part of their official duties; on a worldwide, perpetual, irrevocable, royalty-free basis to BMJ Publishing Group Ltd ("BMJ") its licensees and where the relevant Journal is co-owned by BMJ to the co-owners of the Journal, to publish the Work in this journal and any other BMJ products and to exploit all rights, as set out in our licence.

The Submitting Author accepts and understands that any supply made under these terms is made by BMJ to the Submitting Author unless you are acting as an employee on behalf of your employer or a postgraduate student of an affiliated institution which is paying any applicable article publishing charge ("APC") for Open Access articles. Where the Submitting Author wishes to make the Work available on an Open Access basis (and intends to pay the relevant APC), the terms of reuse of such Open Access shall be governed by a Creative Commons licence – details of these licences and which Creative Commons licence will apply to this Work are set out in our licence referred to above.

Other than as permitted in any relevant BMJ Author's Self Archiving Policies, I confirm this Work has not been accepted for publication elsewhere, is not being considered for publication elsewhere and does not duplicate material already published. I confirm all authors consent to publication of this Work and authorise the granting of this licence.

For peer review only

Clinician and parent views on urine collection in pre-continent children in the UK: a qualitative interview study

Monica Armengol

Department of Engineering Science, University of Oxford

Gail Hayward

Nuffield Department of Primary Care Health Sciences, University of Oxford

Molly Abbott

Guy's and St Thomas' Hospital NHS Trust, London

Christopher Bird

Birmingham Women's and Children's NHS Foundation Trust and Nuffield Department of Primary Care Health Sciences, University of Oxford

Jeroen Bergmann

Department of Engineering Science, University of Oxford

Department of Technology and Innovation, University of Southern Denmark

Margaret Glogowska

Nuffield Department of Primary Care Health Sciences, University of Oxford

Corresponding Author:

Margaret Glogowska

Nuffield Department of Primary Care Health Sciences, University of Oxford

Radcliffe Observatory Quarter, Woodstock Road

Oxford OX2 6GG, UK

Tel: +44 (0)1865 617872

Email: Margaret.glogowska@phc.ox.ac.uk

For peer review only - http://bmjopen.bmj.com/site/about/guidelines.xhtml

Abstract

Objective

To explore the experiences of health care professionals (HCPs) and parents of urine collection methods, to identify barriers to successful sampling and what could improve the process.

Design

Qualitative research, using individual semi-structured interviews with HCPs and parents. The interviews were audio-recorded, transcribed and thematically analysed.

Setting

UK-based HCPs from primary and secondary care settings and parents with experience of urine collection in primary and/or secondary care settings.

Participants

HCPs who were involved in aiding, supervising or ordering urine samples. Parents who had experience of urine collection in at least one pre-continent child.

Results

13 HCPs and 16 parents were interviewed. Two participating HCPs were GPs, 11 worked in paediatric secondary care settings (eight were nurses and three were doctors). Two parents had children with underlying conditions where frequent urine collection was required to rule out infections. HCPs and parents reported that there were no straightforward methods of urine collection for pre-continent children. Each method – 'clean catch', urine bag and urine pad – had limitations and problems with usage. 'Clean catch', regarded as the gold standard by HCPs with a lower risk of contamination, often proved difficult for parents to achieve. Other methods had elevated risk of contamination but were more acceptable to parents because they were less challenging. Many of the parents expressed the need for more information about urine collection.

Conclusions

Current methods of urine collection are challenging to use and may be prone to contamination. A new device is required to assist with urine collection in pre-continent children, to simplify and

For peer review only - http://bmjopen.bmj.com/site/about/guidelines.xhtml

reduce the stress of the situation for those involved. Parents are key partners in the process of urine collection with young children. Meeting their expressed need for more information could be an important way to achieve better quality samples while awaiting a new device.

Strengths and limitations of this study

- We interviewed parents familiar with one or more of the different types of urine collection method and varied lived experience of urine collection.
- Most of the HCPs interviewed worked in environments where unwell children presented every day and where urine collection was a routine part of assessment.
- Interviewing both parents and HCPs gave us a dual perspective on the urine collection process.
- Our study includes relatively small samples of both parents and HCPs.

Patient and Public Involvement and Engagement Statement

This study emerged from an ideation workshop on challenges to urine collection in pre-continent children hosted by the NIHR Community Healthcare MIC in Oxford, UK, May 2019. The Covid-19 pandemic delayed the study's start but found unanimous approval at a virtual meeting of the NIHR Community Healthcare MIC's Patient and Public Involvement and Engagement (PPIE) for children and young people's health in October 2022 and ongoing support at meetings in July 2023 and January 2024.

For peer review only

For peer review only - http://bmjopen.bmj.com/site/about/guidelines.xhtml

Background

Urinary tract infections (UTIs) are common bacterial infections in young children.(1) Diagnosis is challenging as symptoms typical of UTI in adults may be absent.(2) In one study, fewer than one third of children who presented to primary care with acute illness and met microbiological criteria for UTI had been clinically suspected of having a UTI.(3) Thus, there is a need for urine testing to exclude UTI in children where the cause of acute illness is unclear.(4)

In the UK, invasive testing methods to collect urine in pre-continent children such as catheterisation may be used in emergency settings where time is critical but suprapubic aspiration (SPA) under ultrasound guidance is rarely performed. There is debate around whether invasive methods should be used more,(5) although there is evidence that SPA is more painful in infants than catheterisation.(6) UK guidelines emphasise the use of non-invasive methods and recommend invasive methods only if non-invasive methods are not possible or practical.(7) However, non-invasive methods such as 'clean catch', urine bags and nappy pads to collect urine in pre-continent children are challenging to use and associated with high rates of contamination.(4,8,9,10) Contamination of samples presents a significant clinical problem in that it delays diagnosis, can lead to inappropriate prescription and overuse of antibiotics, requires repeat samples which adds healthcare costs and delays discharge, and can result in further unnecessary diagnostic tests such as renal ultrasound scans.(10)

A necessary step toward improving urine collection for pre-continent children is to understand current experiences and how things could be improved. Primary care Health Care Professionals' (HCPs') views of urine collection methods for pre-continent children have been explored using qualitative research methods.(4,11) However, the more holistic picture, including the views of secondary care HCPs, often the first contact for unwell children, as well as those of parents, have not been explored.

For peer review only

Aims

The aims of this study were:

- to understand the experiences and perceptions of HCPs and parents of urine collection methods in primary and secondary care settings
- to identify barriers to successful urine sampling in pre-continent children and what could make the process more efficient

Methods

Design

We chose a qualitative research design – individual semi-structured interviewing – for our study. This design captures people's detailed accounts of their experiences and perceptions of phenomena. It allows researchers to explore their selected topics but gives participants the opportunity to raise issues significant to them. Ethical approval was granted by the Central University Research Ethics Committee, University of Oxford (R77332/RE002) (Supplementary File 1).

Patient and Public Involvement

This study emerged from an ideation workshop on challenges to urine collection in pre-continent children hosted by the NIHR Community Healthcare MIC in Oxford, UK, May 2019. The Covid-19 pandemic delayed the study's start but found unanimous approval at a virtual meeting of the NIHR Community Healthcare MIC's Patient and Public Involvement and Engagement (PPIE) for children and young people's health in October 2022 and ongoing support at meetings in July 2023 and January 2024.

Recruitment and sampling

For peer review only - http://bmjopen.bmj.com/site/about/guidelines.xhtml

For peer review only

We invited HCPs and parents to participate. For HCPs we sought to include secondary care doctors and nurses, as well as general practitioners. Inclusion criteria included aiding, supervising or ordering urine samples in paediatric patients and being able to be interviewed in English. For parents, the inclusion criteria were having experience of urine collection in a pre-continent child within the two years prior to the interview and being able to be interviewed in English. This time period was sufficiently narrow to allow recall of events, but wide enough to ensure that enough parents could be recruited.

Recruitment took place through advertisements on social media groups of parents, charities, and paediatric organisations for HCPs; in newsletters around universities; on posters in nurseries; and on public news boards. The main recruitment material was a leaflet and poster which directed potential participants to the study website. Here they could find more information and register interest. They were then contacted by a research team member (MAr or MAb) to schedule an interview. Participants received written information about the study and had the opportunity to ask questions. Participants gave written consent before interviewing took place.

<u>Data collection</u>

The interviews with HCPs and parents were conducted using separate topic guides around three main issues – the methods and process of urine collection experienced, difficulties encountered, and how urine collection could be made easier. The topic guides were employed flexibly, allowing the interviewers to follow up issues raised by participants, and modified as the study progressed (Supplementary Files 2&3). We did not present any information to the parents about techniques or methods, we gave them the opportunity to tell us in their own words about their experiences. The interviews were conducted online using Microsoft Teams (by MAr and MAb) between December 2021 and July 2022. The mean time for the parent interviews was 35 minutes (range 18-65 minutes) and the mean time for the HCP interviews was 42 minutes (range 25-59) minutes. Methodological

For peer review only - http://bmjopen.bmj.com/site/about/guidelines.xhtml

oversight was provided by an experienced health services researcher (MG). Data collection ended when the researchers agreed that no new issues were raised in the interviews and there was sufficient understanding of the emerging categories. What we were aiming to achieve is ‘information power’ where we had enough material to understand each of the thematic areas in sufficient depth and detail. (12)

Data analysis

Interviews were audio-recorded and transcribed. Transcripts were checked against the audio recordings, de-identified and uploaded to NVivo 12 (Lumivero, Denver, USA), a qualitative analysis software tool. To explore the range of experiences we encountered in the interviews, thematic analysis was used. Data driven analysis allowed us to identify the topics emerging from the HCP and parent data and group them into themes. Initial analysis of both sets of interviews was undertaken by MAr, and an iterative approach to coding, grouping codes into categories and subsequently themes, was taken.(13) MAb and MG reviewed the preliminary themes generated and refined these. These themes and sub-themes were then shared with the wider research team, discussed and agreed to ensure that they were credible and dependable, to enhance trustworthiness.(14,15)

Findings

Sample

42 people expressed interest in participating through the website or email, but not all responded to contact from the research team. Altogether, the sample included 13 HCPs and 16 parents. Among the HCPs, two of the participants were GPs, eight were nurses working in paediatric secondary care settings (including children’s Emergency Departments and other paediatric wards/departments) and three were doctors working in paediatric secondary care settings (including Children’s Emergency department, paediatric surgery and a paediatric department). All HCPs who participated were practising in the UK. Working experience in their current role varied from 7 months to over 30 years.

For peer review only - http://bmjopen.bmj.com/site/about/guidelines.xhtml

Among the parents, eight of the parents had one child and the other eleven had two. Two of the parents had children with underlying conditions where frequent urine collection was required to rule out infections. All but one of the parents interviewed were female. The parents were between 25 and 56 years old. All our participants identified as white, except three who identified as mixed race or Indian.

Among our sample of parents, eight had experience of clean catch only, with five of these wanting to try the urine bag method; five had experience of clean catch and urine bag; one had experience of clean catch and nappy pad; one had experience of clean catch, urine bag and nappy pad; and a further one had experience of urine bag only. Experience of methods other than 'clean catch' was more limited, as many UK hospital guidelines advise against other methods of urine collection because of the high risk of contamination. Two had experience of invasive methods – in and out catheterisation.

From the analysis of the interviews, two main themes emerged: acceptability and challenges of urine collection methods; and making the urine collection process more parent- and child-friendly.

<u>Acceptability and challenges of urine collection methods</u>

<u>*Invasive methods*</u>

HCPs and parents agreed that invasive urine collection methods, such as in and out catheterisation and suprapubic aspiration (SPA), were acceptable when a child was seriously unwell and a urine sample was urgently needed. For parents, SPA seemed the least acceptable method because they perceived it as painful for their child. Similarly, HCPs tended to regard SPA as the most invasive method and did not commonly perform it. One clinician regarded it as useful only if imaging was used to guide collection of the sample. Four of the secondary care clinicians performed catheterisation but this happened infrequently. Both parents and HCPs believed that invasive methods had additional risks, particularly introducing infection, limiting their acceptability.

For peer review only - http://bmjopen.bmj.com/site/about/guidelines.xhtml

<u>*Non-invasive methods*</u>

Clean catch

Among the non-invasive urine collection methods, 'clean catch' was preferred in the primary and secondary care settings where the HCPs we interviewed worked and the parents we interviewed sought care for their children. This usually involves direct catching of urine spontaneously produced by the child into a sterile container such as a pot or a bowl provided for that purpose. Several HCPs described this process as a *"nightmare"* with pre-continent children, acknowledging that it can be demanding for parents, messy, difficult to judge and time-consuming.

Parents found the 'clean catch' process challenging, if not impossible. They highlighted how emotional the process could be, also making it less acceptable. Very young unwell children often became upset at the process which further increased the stress for parents. The 'clean catch' method often required the child to be unclothed, at the least have their nappy removed, so the parent could see when urination occurred. Some parents and children found this aspect very upsetting. Even adapting the method to obtain a sample at home, such as holding a child over the bath, could be distressing. Knowing that they needed to obtain a sample to help pinpoint why their child is ill but having little control over when and if they can get one, increased the stress felt by parents. HCPs concurred that using the 'clean catch' method often meant that samples could be very delayed.

HCPs acknowledged that while the clean catch method was pursued to avoid contamination, the reality of attempting it diverged from the ideal. They were aware that sterile containers provided did not always remain sterile and that it was difficult, if not impossible, for parents to prevent the container coming into contact with the child's skin. Some parents were aware of how contamination could occur but found it difficult to avoid. To reduce contamination from a 'clean catch' sample,

For peer review only - http://bmjopen.bmj.com/site/about/guidelines.xhtml

several HCPs suggested that HCPs advise parents to wipe their child's genital area beforehand. Parents, however, reported not having received this instruction.

Urine bags

HCPs, particularly the GPs in our sample, and some parents, mentioned urine bags for urine collection but these were not used in all of the settings where our participants either worked or attended with their children. These are plastic bags placed around the child's genitalia using an adhesive which sticks to the skin. HCPs found the idea acceptable in principle as they caught urine without parents needing to follow their child around with a container. However, HCPs highlighted issues such as high contamination rates, leakage through loss of adhesion and difficulty with fitting the bag around the genitalia of both boys and girls, making them far less acceptable. Parents who hadn't used urine bags were optimistic about trying them. However, parents who had already used them found them far less acceptable in practice. They described similar issues to those of the HCPs – contamination, leakage, the bag losing adhesive and dropping off, and discomfort when the bag was pulled away from the child's skin in sensitive areas.

Urine pads

Normally used for children younger than 6 months, urine pads were mentioned less frequently than 'clean catch' and urine bags. These are absorbent pads positioned inside the child's nappy from which urine is collected via a syringe or squeezed out. HCPs regarded them as acceptable but acknowledged their problems, which included higher contamination rate than 'clean catch' and difficulty knowing whether any urine has been collected. Few parents had experience of urine pads for urine collection. A couple of parents mentioned using improvised 'pads' of sponge or cotton wool when desperate to obtain a sample at home, acknowledging that this was not ideal.

Table 1 contains a summary of this theme **Acceptability and challenges of urine collection methods**.

For peer review only - http://bmjopen.bmj.com/site/about/guidelines.xhtml

Table 2 summarises the acceptability criteria for non-invasive urine collection methods discussed by parents and HCPs. For HCPs the acceptability of a non-invasive method of urine collection was largely determined by contamination risk. This appeared to be the case for both primary and secondary care clinicians. In the primary and secondary care settings where the HCPs we interviewed worked, invasive methods tended to be used very infrequently and much less often than the default ‘clean catch’ method. Both sets of clinicians, however, acknowledged that this method often presented a considerable challenge for parents.

For parents, charged with obtaining the sample, acceptability was centred more around the difficulty of the method, the time taken and whether it caused their child distress.

<u>Making the urine collection process more parent- and child-friendly</u>

<u>Communication between parents and HCPs</u>

Parents often reported a lack of clear communication about urine collection from HCPs. Some parents felt they lacked an explanation of why a urine sample was required, while other parents felt it was only partially explained. Parents also suggested that HCPs assumed that they would know how to perform ‘clean catch’. In one situation a parent was given a urine bag to use at home but needed to find instructions on the Internet. Parents often felt concern that they were obtaining samples correctly. Some were aware of contamination but lacked a step-by-step explanation of where it occurs and how to avoid it.

Some of the HCPs acknowledged spending little time giving parents full instructions. When asked if it was standard procedure for HCP to give parents instructions, one HCP thought there should be policies and written standards. Additionally, this HCP felt it was important to have guidelines around when to collect urine samples, so that parents were not burdened with it unless a sample was necessary for clinical decision-making.

For peer review only - http://bmjopen.bmj.com/site/about/guidelines.xhtml

Information for parents

HCPs and parents were asked about what could improve communication about urine collection. They agreed that formal instructions in a leaflet provided along with the urine collection container would be useful. HCPs were positive about giving parents written instructions as there was little time to pass on verbal instructions. Posters in clinical rooms where parents could read information were also viewed positively and one HCP suggested making a video parents could watch.

Parents and HCPs were asked about acceleration methods – actions which encourage or speed up urination. Mostly HCPs just recommended giving the child regular drinks but some were aware of other methods, such as turning taps on in the background, running a cool fan near the child, pouring warm water over the child's abdomen, and putting either warm or cool wet paper towels above their pubic area (Quick-Wee method).(16) Some HCPs found the tap method gave good results but was not practical, as it required a number of people to be involved. These methods were normally suggested only after time had elapsed without a sample collected. Few parents reported receiving any tips about speeding up urination. One parent accidentally found that as soon their child's nappy was removed and he stood on a cold floor urination happened. Parents, however, felt they would have appreciated suggestions from HCPs around acceleration methods even if they did not guarantee success.

Towards a new device design

Both parents and HCPs agreed on the need for a new device to simplify urine collection and reduce the stress of obtaining samples. Parents appreciated that no device could automatically reduce waiting time for urination but that it should enable clean, efficient collection when urination did occur. Parents were highly critical of the containers provided for urine collection, especially narrow pots designed for adults which they knew adults also struggle with. They felt that any new device

For peer review only - http://bmjopen.bmj.com/site/about/guidelines.xhtml

using a container should be tailored according to sex and be wider to allow easier collection without touching the skin.

Parents and HCPs described the criteria they considered important in making a new device fit for purpose. A high priority for parents was a non-invasive device which didn't require constant monitoring. Parents and HCPs described how current methods could be adapted to make urine collection easier, such as a urine bag which doesn't need adhesive to stay in place. One HCP described how something like a nappy which would fit on the child would be helpful. Parents also favoured a wearable device for urine collection from a child resting or lying down. Several parents responded very negatively to removing their child's nappy and other clothing during the wait for a sample, especially where this caused distress. Thus, a device which could be used while the child remained clothed was considered more appropriate. Secondary care HCPs concurred that such a device would reduce the need for private spaces during the process.

HCPs perceived that simplicity of use was crucial in a new device so that staff could learn to use it quickly. Cost and any need for training in using a new device were also major considerations. Disposal was considered important by both parents and HCPs but both groups accepted that any device would probably need to be single use plastic as sterilisation was not likely to be possible.

Table 3 contains a summary of this theme **Making the urine collection process more parent- and child-friendly**.

Discussion

Invasive methods have a place in urine collection with pre-continent children but non-invasive methods are more commonly experienced in both the primary and secondary care settings, from which we drew our sample. We found that there were no straightforward non-invasive methods of

For peer review only - http://bmjopen.bmj.com/site/about/guidelines.xhtml

urine collection for pre-continent children. HCPs and parents explained that each method described – 'clean catch', urine bag and urine pad – had limitations and problems with usage. 'Clean catch', the urine collection method regarded as the gold standard by HCPs and with a lower risk of contamination, often proved difficult for parents to achieve. Other methods had elevated risk of contamination but were more acceptable to parents because they were less challenging and are still advocated as a last resort by UK national guidelines.(7)

Our findings highlighted ways in which current urine collection practice could be improved for parents and pre-continent children. While there is evidence that quicker sample collection can occur by the 'Quick-Wee' method,(16) acceleration methods did not appear to be routinely practised by the HCPs we interviewed, as they required a number of staff members to be involved,(17) and parents were typically not informed about them. Although GPs who had tried this method considered it impractical, such information could potentially improve the urine collection process for parents.(2) Parents would have appreciated clearer communication from HCPs about why a urine sample is necessary and they often expressed the need for more information and support for collecting urine samples, as these may determine the management of their child's illness. Explanation of the ways in which contamination can occur and how to avoid this would have been welcome to parents. Leaflets, posters and information videos were ways mentioned to give parents greater awareness of the issues around urine collection. This has a parallel with the information needs highlighted in a study conducted with adult women experiencing UTIs.(18)

Our findings also demonstrate the need for improved urine collection methods, such as a device which could make the urine collection process less challenging for parents and less distressing for the children. Previous literature, detailing the strengths and shortcomings of various methods of urine collection, including all of those discussed in our study, also points towards the need for new methods.(2,4,11)

For peer review only - http://bmjopen.bmj.com/site/about/guidelines.xhtml

Strengths and limitations

Our study includes relatively small samples of both parents and HCPs who presented for interview. However, the sample includes a number of parents familiar with several of the different types of urine collection method and varied lived experience of urine collection. Similarly, the majority of the HCPs worked in environments where unwell children presented every day and where urine collection was a routine part of assessment.

Previous qualitative research has been conducted around UTI in children, but has focused on parents' symptom recognition and management of the condition rather than the experience of urine collection.(1) One study has explored the views of GPs about urine collection in pre-continent children and described the barriers and facilitators to the process in Australian primary care settings.(11) In our study we were able to explore the views of parents, GPs and secondary care clinicians, which has been lacking in the literature up until this point. Thus, we were able to achieve a dual perspective on urine collection. Comparing and contrasting the expectations and experiences of HCPs and parents about what made methods more or less acceptable (risk of contamination, ease of collection, the amount of time taken for urine collection and urine collection causing distress to the child) helped us to identify where mismatches occurred and suggest why.

A limitation of the study is the method of recruitment of the parent sample. Ideally, we would have recruited parents at the point of needing to collect a urine sample from their child. However, this was not possible due to time and funding constraints, as well as concerns about the feasibility of recruiting in busy urgent care settings. It is possible that this recruitment method could have resulted in selection bias, with parents participating in order to recount negative experiences. Recall bias could also have affected our findings, although in the interviews, it was evident that these were

For peer review only

For peer review only - http://bmjopen.bmj.com/site/about/guidelines.xhtml

significant experiences for the parents and none of them expressed any difficulty in recalling the events we asked them about.

Conclusions

Current methods of urine collection are challenging to use and may be prone to contamination. There is need for a new device to assist with urine collection in pre-continent children. Ideally, this would simplify and reduce the stress of the situation for those using it whilst reducing the contamination risk. Parents are key partners in the process of urine collection with young children. Meeting their need for more information and support in the process of collecting urine could be an important way to achieve better samples while waiting for a new device.

References

1 Campbell A, Hartling L, Louie-Poon S, Scott SD. Exploring the Experiences and Information Needs of Parents Caring for a Child With a Urinary Tract Infection: A Qualitative Study. *J Patient Exp*. 2021;8:1-8.

2 Boon H, Van den Bruel A, Verbakel J. GPs' perspectives on diagnosing childhood urinary tract infections: a qualitative study. *Br J Gen Pract.* 2022; DOI: https://doi.org/10.3399/BJGP.2021.0589

3 Butler CC, O'Brien K, Pickles T, et al. Childhood urinary tract infection in primary care: a prospective observational study of prevalence, diagnosis, treatment, and recovery. *Br J Gen Pract.* 2015; DOI: 10.3399/bjgp15X684361.

4 Boon HA, Lenaerts W, Van Aerde C, Verbakel JY. Outpatient urine collection methods for paediatric urinary tract infections: Systematic review of diagnostic accuracy studies. *Acta Paediatr.* 2021;110:3170– 3179. https://doi.org/10.1111/apa.16027

For peer review only - http://bmjopen.bmj.com/site/about/guidelines.xhtml

5 Cousin E, Ryckewaert A, de Jorna Lecouvey C, Arnaud AP. Urine collection methods used for non-toilet-trained children in pediatric emergency departments in France: A medical practice analysis. *Archives de Pédiatrie*. 2019;26:16–20.

6 Kozer E, Rosenbloom E, Goldman D. Pain in Infants Who Are Younger Than 2 Months During Suprapubic Aspiration and Transurethral Bladder Catheterization: A Randomized, Controlled Study. *Pediatrics*. 2006;118, 1:e51-e56.

7 NICE, Urinary tract infection in under 16s: diagnosis and management NICE guideline Published: 27 July 2022 www.nice.org.uk/guidance/ng224

8 Butler CC, Sterne JA, Lawton M, et al. Nappy pad urine samples for investigation and treatment of UTI in young children: the 'DUTY' prospective diagnostic cohort study. *Br J Gen Pract.* 2016;66(648):e516-524.

9 Tosif S, Kaufman J, Fitzpatrick P, et al. Clean catch urine collection: Time taken and diagnostic implication. A prospective observational study. *J Paediatr Child Health*. 2017;53:970–975.

10 O'Gorman J, Clarke J, Robinson M. 'Spending a penny, but keeping it clean' – reducing the rate of contaminated non-invasive samples in the paediatric department. *Arch Dis Child.* 2022;107(Suppl 2):A429–A430.

11 Kaufman J, Sanci L, Temple-Smith M. What's the catch? Urine sample collection from young pre-continent children: a qualitative study in primary care. *BJGP Open*. 2020; DOI: 10.3399/bjgpopen20X101060.

12 Malterud K, Siersma VD, Guassora AD. Sample Size in Qualitative Interview Studies: Guided by Information Power. *Qual Health Res* 2016;**26**. doi:10.1177/1049732315617444

13 Silverman D. *Doing qualitative research: a practical handbook.*2018. London: Sage Publications: 2018.

14 O'Brien BC, Harris IB, Beckman TJ, Reed DA, Cook DA. Standards for reporting qualitative research: a synthesis of recommendations. Acad Med. 2014;89(9):1245-1251.

15 Lincoln YS, Guba EG. *Naturalistic inquiry.* 1985. Beverly Hills, CA: Sage Publications; 1985.

For peer review only - http://bmjopen.bmj.com/site/about/guidelines.xhtml

16 Kaufman J, Fitzpatrick P, Tosif S, et al. Faster clean catch urine collection (Quick-Wee method) from infants: randomised controlled trial. BMJ. 2017;357(j1341):1–8.

17 Herreros Fernández ML, González Merino N, Tagarro García A, et al. A new technique for fast and safe collection of urine in newborns. *Arch Dis Child.* 2013;Jan;98(1):27-9. doi: 10.1136/archdischild-2012-301872.

18 Glogowska M, Croxson C, Hayward G. Women's information needs around urine testing for Urinary Tract Infections: qualitative study. *Br J Genl Pract. 2022;*DOI: https://doi.org/10.3399/BJGP.2021.0564

For peer review only

For peer review only - http://bmjopen.bmj.com/site/about/guidelines.xhtml

Tables

Table 1 Summary of theme 'Acceptability and challenges of urine collection methods'

Subtheme	Topic and example quotations
Invasive methods	***Suprapubic aspiration and catheterisation***<br>But if you've got a very unwell baby and there's a time pressure to collect a sample, then essentially you either have to get a catheter sample, or if absolutely required, then very occasionally they would do a suprapubic aspiration [Interview 562, nurse, children's Emergency Department [ED]]<br><br>I would quite happily have allowed them to catheterise her…because she was very poorly…and if that could have meant that we just got some urine out and got an answer and could go home I would have taken that option. I don't think I would want that on the other occasions because it's not particularly nice for a child to have a catheter [Int 600, parent]<br><br>I haven't done a suprapubic aspirate for absolutely years and years…but generally, if you don't do them with imaging to help you then it's a bit pointless. [Int 767, consultant, children's ED]<br><br>***Risks around invasive methods***<br>If life or health could be permanently damaged by waiting an hour, then do what you need to do now. But obviously, it wouldn't have been ideal because…I'm sure using a catheter presumably carries some risk of infection and damage. [Int 869, parent]<br><br>If you were doing [an] invasive technique on everyone then you also put them at risk of introducing infection. They might not have one in the first place and then you can use a poor technique they're going to end up with one [Int 327, paediatric nurse, ED]
Non-invasive methods	***Challenges of the 'clean catch' method***<br>It can take ages and they [parents] missed the urine quite a bit. It ends up on the floor, on the bed, so it's not easy. [Int 984, advanced clinical practitioner, children's ED]<br><br>I mean, they're wildly, wildly unpredictable. And yeah, it is very tiring. It is very challenging because obviously, they don't wee all that frequently, so if you miss it, then it really delays their care. [Int 298, paediatric surgeon, secondary care]<br><br>She [was] really quite distressed trying to put [her] in a position, trying to catch the urine for them…She's getting upset screaming at me. I'm trying to catch it…she keeps kicking the bowl… and it's just an absolutely nightmare [Int 535, parent]<br><br>It wasn't warm…she had to be naked and she was poorly…I thought well, if I put the nappy on, I wouldn't know when she's weeing to be able to then act quickly to move her on top of the pot that was given. So I felt quite bad as a mum when she's crying and unwell and laying there really cold and you're having to try and just get on with it to try and get one [a sample]. [Int 266, parent]<br><br>I have a bit of a blurry memory of holding a baby over a bath at three in the morning. With the husband holding a pot trying to wait for a wee basically…you've done a feed and you're just waiting for pee…she was not happy to be held…held naked just kind of held a bit awkwardly for about 10, 15 minutes. She was not happy about that. She was crying quite a lot. [Int 088, parent]<br><br>There was a kind of time pressure…we need this checked off. And the sooner I can get the sample the sooner we can exclude that there's anything really dangerous going on. [Int 869, parent]

For peer review only - http://bmjopen.bmj.com/site/about/guidelines.xhtml

Have the child out of a nappy and catch it when they wee, which obviously spends a long, long time…but then it means that everything is delayed. [Int 630, paediatric urology nurse, secondary care]

Contamination and 'clean catch'

But if you're sat there with the sterile pot for any period of time, it's gonna get touched by… the parent's finger. [Int 998, paediatric nurse, secondary care]

With the pot you've got to keep it very close to them, they've got to make sure that they're trying not to touch it [Int 980, advanced nurse practitioner, children's ED]
trying to not allow, the genitalia to touch the funnel, I don't think it's achievable. [Int 562, senior nurse, children's ED]

So I have had the words "get a sterile sample"… and I just sort of think well that's really hard, especially when I was having to do it in such a way when…she was lying down and you were trying to hold a little collection tube or something under. It was touching, you try not to touch the skin or anything, but you kind of have to, and then…I knew it wasn't clean, like as clean as they would want it, because it just it was just impossible. [Int 535, parent]

We've got some wipes, you want to clean the area before we wait particularly, you know, mainly the girls really the girls are worse. [Int 016, paediatric registrar, secondary care]
Nobody tells you make sure their bum is really clean or gives you anything to clean their bum with before they sit in this bowl [Int 307, parent]

Experiences around urine bags

The bags were much easier. You were guaranteed to catch, to capture it, you could put the nappy back on. They can run around, you can put them back in the waiting room. [Int 984, advanced clinical practitioner, children's ED]

There's the little bags that you stick that go inside the nappy but I haven't used them once in my job now…but from what I've been told by colleagues they notoriously don't stick. The urine leaks out of it, also they're not necessarily as sterile. [Int 543, nurse, children's ED]

Especially that bag, I would have given it a go. I suppose all children are different but…I think that bag one probably would work better than just looking out for wee and when she's going and having it [a pad under her] on the floor ready. [Int 266, parent]

It's not simple either [using the bag], because, it does stick on her and I think when you kind of peel it off, it leaves like a residue on her, so I think even like peeling it off at the end is a little bit probably a little bit painful, kind of like a bandaid, on those sensitive parts coming off probably does not feel good for her [Int 698, parent]

Experiences around urine pads

We can't do it that way because it's not a clean sample. So then I'll explain to them like if it does come up as positive for infection, and it's not a clean sample, we then have to repeat the sample anyway. [Int 327, paediatric nurse, ED]

Pads are not quite so easy because you can't tell that they've been used without looking at them… and in the past it just used to be just like I have to touch the nappy to see whether it was wet or not [Int 630, paediatric urology nurse, secondary care]

I used a new sponge and I cut it in half and I just put it between her legs and put a pair of pants on rather than a nappy…then when I felt she'd weed on me, I quickly grabbed the sponge and just wrung it out…I know that was probably classed as contaminated in some way [Int 266, parent]

For peer review only - http://bmjopen.bmj.com/site/about/guidelines.xhtml

Table 2 Acceptability criteria for urine collection using non-invasive methods in young children for HCPs and parents

	HCPs	Parents
**Preventing contamination in urine samples**	Highly important in determining acceptability of the method.<br><br>Uncontaminated samples necessary to guide diagnosis and treatment. 'Clean catch' recommended for this reason.	Less awareness of the importance of uncontaminated samples so parents appeared to consider this less in determining acceptability of the method.
**Ease of collection**	HCPs not involved in collection process so do not always experience the difficulties encountered by parents.<br><br>HCPs aware that 'clean catch' is challenging but still consider it the best option.	Highly important in determining acceptability of the method.<br><br>'Clean catch' perceived as very difficult and thus other methods, seen as less difficult but where contamination rates are higher, may be preferred.
**Amount of time taken for urine collection**	Important for HCPs as delay in urine collection can delay diagnosis/treatment.<br><br>HCPs aware that 'clean catch' can take a long time but still consider it the best option.	Very important as urine collection process can be lengthy.<br><br>'Clean catch' can require vigilance over long periods of time from parents. This can be particularly challenging if urine collection taking place in hospital.
**Urine collection causing distress to child**	HCPs not involved in urine collection process so do not always experience the distress caused to the child first hand.	Very important as child's distress, especially if undressing is required, during urine collection process adds to parental stress.

For peer review only

For peer review only - http://bmjopen.bmj.com/site/about/guidelines.xhtml

Table 3 Summary of theme 'Making the urine collection process more parent- and child-friendly

Subtheme	Topic and example quotations
Communication between parents and HCPs	***Explanation of the need for a urine sample***<br>It seemed quite random and unnecessary to then do it [get a urine sample]…the link wasn't really explained to me what the need for the sample was…so I tried, but yeah, it wasn't really clear as to what we were looking for in the sense of what she'd seen and why she felt she needed one [a sample]. [Int 266, parent]<br><br>They said that it [urine sample] would be worth having…because all of the other ones [tests] came back alright. It was one of those extra things to check. [Int 519, parent]<br><br>***Information about the 'clean catch' and other methods***<br>They just gave me the containers and said to send it back when I had collected it. [Int 881, parent]<br><br>In your cubicle [in hospital], they didn't give any specific instructions on how to collect. So they gave me the pot and said just make him pee here. And that was pretty much all the instructions that I received. [Int 479, parent]<br><br>No, I wasn't actually [told how to use the bag at home]. I think I looked it up online just to check. [Int 009, parent]<br><br>I've been told you know, be careful that doesn't get contaminated [urine container] but how particularly I do that I don't know…I've ended up following them around with Tupperware pots that I wiped with like sterilising wipes first…I mean I doubt it's that clean…I take their nappy off… but I didn't sort of do anything in terms of cleaning around their bottoms or anything. [Int 600, parent]<br><br>***Giving parents instructions***<br>I'm probably not very good at it actually. I probably say we're gonna need a urine and give them the collection pot and show them sort of how it works and just sort of say…it's a case of sitting there and waiting until they pee…I think I mean, you could blame time, but I think that's an excuse, not a reason…it's something we're doing all the time and they're not. [Int 998, paediatric nurse, secondary care]<br><br>***The need for policies around urine collection to help parents***<br>The amount of times people are set up for urine collections, there is a policy within the trust about urine collection, but I think it should be more specific about methods…..(When I) went up to the acute admissions unit, the nursing staff up there had never really seen it [a urine collection in pre-continent children] done, even their senior ones. So that information [about collection methods], I think sometimes gets lost unless you put it within a policy…I didn't think that they're [junior doctors] given enough education, and I certainly there's not a standard for it. [Interview 562, senior nurse, children's ED and acute ward]<br><br>I don't think some of them, specially when the junior doctors come through, they don't necessarily recognize how difficult it is for families to collect urines to make sure that they do definitely need them rather than arbitrarily saying, oh, I need a urine, yes, but that might take 2 hours and do you really need it for your clinical decision making? Is there an option to let the family go home and do it, you know? [Interview 562, senior nurse, children's ED and acute ward]
Information for parents	***Providing better information about urine collection for parents***<br>I think a leaflet is fine. Especially if there's like a diagram or something. Yeah, if a doctor wants to take the time to explain that as well, that's helpful…and then if you have questions,

For peer review only - http://bmjopen.bmj.com/site/about/guidelines.xhtml

	obviously, you can ask them…What happens a lot is that people assume…that you've been told stuff already. [Int 507, parent]<br><br>It would be something that had fairly clear instructions for the parent, and that were sort of easy to convey fairly quickly too. Because, obviously, there's always the time pressures in general practice. [Int 201, GP]<br><br>We were actually thinking about doing a bit of a quality improvement project in our department…making some videos on common procedures…that the parents could watch. So that was a way to potentially think about making it a bit more consistent as to what is explained. [Int 980, advanced nurse practitioner, children's ED]<br><br>***Information about 'acceleration' methods***<br>So the first and most definite, if the kid doesn't drink then nothing is going to come out. [Int 285, staff nurse, secondary care]<br><br>But the problem is with the tap method, why it's not used as much as because it takes three people to do it. [Int 562, senior nurse, children's ED and acute ward]<br><br>I think putting his feet on the floor because it was cold made him wee. I tried blowing as well 'cause that's, you know, as soon as you take a boys nappy off they usually wee… [Int 451, parent]
Towards a new device design	***Desired characteristics of a new device***<br>I think the other thing that really struck me was the shape of the sample pot. Seemed really non ideal for I mean, if it had been more like a funnel or something like that, that [could] have managed to catch a stream of urine in a really narrow pot. [Int 869, parent]<br><br>Something like the urine bag, but that is easier to attach and to detach, because he has been stressed when we have tried taken it off. [Int 881, parent]<br><br>I think ideally, there would be something that you can just take the baby out of their nappy and put on another nappy and wait for them to wee and then have got a sterile sample…So I think we're removing the kind of human element of having to watch and wait and time to catch it, that would be the best thing. [Int 298, paediatric surgeon, secondary care]<br><br>I think maybe that whatever item that might be designed, it needs to sort of be easy to use that any and everyone can like learn how to use it fairly quickly because obviously there are a lot of GPs in the practice. [Int 570, GP]<br><br>Our trust would never pay I don't think for expensive kit. If it can be done cheaply, then that's the way to do it. [Int 767, consultant, children's ED]<br><br>It's just something simple, something that the parents or the staff can easily use. And then I suppose easily disposed of as well. [Int 630, paediatric urology nurse, secondary care]

For peer review only - http://bmjopen.bmj.com/site/about/guidelines.xhtml

Declarations

Ethics approval and consent to participate

Ethical approval was granted by the Central University Research Ethics Committee, University of Oxford (R77332/RE002). Participants received written information about the study and had the opportunity to ask questions. Participants gave written consent before interviewing took place.

Availability of data and materials

The datasets generated and analysed during the current study are not publicly available because participant consent to share the original data outside the research team was not sought but reasonable requests for further information which does not compromise participant anonymity and confidentiality may be obtained from the corresponding author.

Competing interests

The authors declare that they have no competing interests.

Funding

This work was supported by the University of Oxford (Grant number: N/A) and the National Institute for Health Research (NIHR) (Grant number: MIC-2016–018).

Acknowledgements

We would like to thank all the parents and clinicians who took part in interviews as well as the NIHR Community Healthcare MIC's Patient and Public Involvement and Engagement (PPIE) for children and young people's health for their support for our study.

Authors' contributions

MAr, JB, GH, CB and MG were involved in the design of the study. MAr and MAb recruited participants, collected all the data and led analysis of the data, supervised by MG and GH. All authors were involved in interpretation of the analysed data and contributed to the final manuscript. All authors read and approved the final manuscript.

Word count

3719 words

For peer review only - http://bmjopen.bmj.com/site/about/guidelines.xhtml

SECTION A. RESEARCH DETAILS	
**1. Full title of research**	Exploring perspectives, priorities, and solutions of urine collection: A qualitative user centric study to understand practices and gain feedback from healthcare professionals and parents/carers
**2. Short title of research**	Urine collection qualitative studies
**3. Principal Investigator (PI) / Student Supervisor**	Dr. Jeroen Bergmann
**4. PI's training in research ethics and/or GCP**	"Children and Clinical Research" from the Global Health Training Centre, Course completed on 31/08/2020 JB<br><br>"ICH Good Clinical Practice (GCP) E6", from the Global Health Training Centre, Course completed on 31/08/2020 JB<br><br>"Research Integrity", University of Oxford, Course completed on 31/08/2020 JB<br><br>"Research Integrity in Engineering and Technology", University of Oxford, Course completed on 26/02/2015 JB<br><br>"Introduction to Research Ethics" from the Global Health Training Centre, Course completed on 19/01/2015 JB<br><br>"Human Research", Basic Course, Collaborative Institutional Training Initiative, MIT, Completed on 12/11/2013 JB
**5. Student name and degree programme (if applicable)**	
**6. Department/Institute name**	Department of Engineering Science
**7. University email address**	jeroen.bergmann@eng.ox.ac.uk
**8. University telephone number**	
**9. Funding Source**	EPSRC IAA / NIHR BRC
**10. State any conflicts of interest and explain how these will be addressed**	None

For peer review only - http://bmjopen.bmj.com/site/about/guidelines.xhtml

SECTION B. RESEARCHERS

**1. Researcher title and name**	Dr. Monica Armengol
**2. Department / Institute name**	Department of Engineering Science
**3. Role in research**	Researcher will obtain informed consent and conduct research activities with participant
**4. Training in research ethics and/or research integrity**	"Children and Clinical Research" from the Global Health Training Centre, Course completed on 23/06/2021 MA<br>"Research Integrity", University of Oxford, Course completed on 24/06/2021 MA
**5. Researcher title and name**	Dr. Christopher Bird
**6. Department / Institute name**	MIC Oxford Health NHS Foundation Trust
**7. Role in research**	Design and supervision of project, liaise with medical professionals
**8. Training in research ethics and/or research integrity**	GCP up to date July/2021 (Health Research Authority, the governing body for NHS Ethics in the UK)
**9. Researcher title and name**	Dr. Gail Hayward
**10. Department / Institute name**	Primary Care Health Science
**11. Role in research**	Design and supervision of project
**12. Training in research ethics and/or research integrity**	GCP, 2019 (Health Research Authority, the governing body for NHS Ethics in the UK)
**13. Researcher title and name**	Dr. Philip Turner
**14. Department / Institute name**	Primary Health Care Science
**15. Role in research**	Design and supervision of project
**16. Training in research ethics and/or research integrity**	"Research Integrity", University of Oxford, Course completed on 21/09/2021
**17. Researcher title and name**	Nathaniel Mills
**18. Department / Institute name**	NIHR Children & Young People MedTech Cooperative

For peer review only - http://bmjopen.bmj.com/site/about/guidelines.xhtml

**19. Role in research**	Support and supervision. Liaise to medical professionals
**20. Training in research ethics and/or research integrity**	NIHR Good Clinical Practice course
**21. Researcher title and name**	Dr. Paul Dimitri
**22. Department / Institute name**	NIHR Children & Young People MedTech Cooperative
**23. Role in research**	Support and supervision. Liaise to medical professionals
**24. Training in research ethics and/or research integrity**	GCP (Health Research Authority, the governing body for NHS Ethics in the UK)<br>GCP Paediatrics Module (Health Research Authority, the governing body for NHS Ethics in the UK)
**25. Researcher title and name**	Gemma Wheler
**26. Department / Institute name**	NIHR Children & Young People MedTech Cooperative
**27. Role in research**	Design of study and analysis support
**28. Training in research ethics and/or research integrity**	NIHR Good Clinical Practice course
**29. Researcher title and name**	Molly Abbott
**30. Department / Institute name**	Oxford Health NHS Foundation Trust and University of Oxford
**31. Role in research**	Recruitment of Interviewees and performing interviews
**32. Training in research ethics and/or research integrity**	Oxford GCP training course for clinical researchers 28 March 2021

**SECTION C. BASIC INFORMATION**	
**1. Provide a brief lay summary of the aims and objectives of the research. This should cover the questions it will answer and any potential benefits.**<br><br>**(Maximum 300 words)**	Urine is used to diagnose and monitor different diseases including diabetes and urine tract infections (UTI). Paediatric UTI accounts for 14% of the emergency department visits. Despite the high occurrence, collection of urine samples in non-toilet trained infants is challenging, lengthy and up to 30% of urine samples are contaminated with bacteria from the back passage or skin, making results meaningless.<br><br>Re-testing urine samples due to contamination causes delays, increases visits to the GP and emergency services. Additionally, it is associated with misdiagnosis, which can cause bacterial resistance if children are prescribed an antibiotic due to erroneous diagnosis.<br><br>This study aims to understand the current practices of urine collection in non-toilet trained infants and the challenges faced when carrying out, ordering or waiting for a collection.

For peer review only - http://bmjopen.bmj.com/site/about/guidelines.xhtml

	These findings will guide the focus areas for the development of a new device for paediatric urine collection, requirements, and specifications of the device.<br><br>We believe that a user-centric approach in the development of medical devices, produces device of higher clinical value. Participants will provide input in two parts: (1) interviews will aim to explore practices, costs, experiences, and perceptions of available diagnostic technology used for urine collection in infants in clinical settings. Additionally, we will explore unmet needs with regards to urine collection technologies and the potential impact of new diagnostic methods, tools or technology.<br>(2) a co-design workshop will allow participants to have input into possible solutions to the urine collection issues reported during the interviews.
**2. List all places where research will be conducted (including any other countries and online)**	Most interviews and workshop will be conducted online, via teams/zoom. Interviews will be conducted out of NHS premises.
**3. Anticipated research start date**	As soon as ethics is approved. Oct-Nov 2021
**4. Anticipated research end date**<br><br>(n.b. A maximum of 5 years approval can be granted)	3 years after approval
**5. Please list any CUREC Approved Procedure(s) you will follow**	
**6. Please list any CUREC Best Practice Guidance used to develop your research**	BPG 01 Researcher Safety/ BPG 03 Elite and expert interviewing/ BPG 09 Data collection, protection and management/ BPG 10 Conducting research interviews
**7. Name of departmental / peer reviewer (if applicable)**	Engineering Science / Sarah Hoeksma

**SECTION D. PARTICIPANTS**	
**1. Age range of participants**	Between 18 and 70 years old
**2. Are research participants classed as people whose ability to give free and informed consent is in question?**	Yes
**3. Anticipated number of participants**	Around 30 healthcare professionals and 20-30 parents

For peer review only - http://bmjopen.bmj.com/site/about/guidelines.xhtml

**4. How was the number of participants decided?**	We anticipate conducting between 20-30 interviews to healthcare professionals and 20-30 interviews to parents/carers. However, recruitment will continue until data saturation is reached or sufficient exploratory power for the findings is achieved. Further interviews might be required to fully explore the topics proposed. The research team will decide on concluding interviews.<br><br>For the workshop, we anticipate around 30 people taking part. This will include parents and healthcare professionals that took part in the interviews, in addition to other parents, health care professionals, engineers, designers and general public that are interested in taking part.
**5. Inclusion criteria**	Healthcare Professionals<br><br>Consenting adults with experience working with urine collection whether direct or indirect (perform, assist, manage, purchase, order or interpret) within a clinical setting. These include medical professionals, nurses, administrative staff and bioanalyst.<br><br>Parents/Carers:<br><br>Consenting adults with experience aiding a pre-continent child collect a urine sample in the last 2 years.<br><br>The adults taking part in the interviews, both parents/carers and healthcare professionals, must be able to take part on the interview conducted in English and consent to the interview being recorded. Participants must be over the age of 18 years and below 70 years old.<br><br>Co-design workshop<br><br>Consenting adults over the age of 18 years and below 70 years old with an interest in urine collection in infants, including those that took part in the interviews. Additionally, engineers, designers, industry experts and academics will be invited.
**6. Exclusion criteria**	The participant may not enter the study if they are unable to take part in an interview in English or written consent cannot be provided.

**7. Please mark 'X' against all planned recruitment methods**<br><br>Provide copies of all recruitment material for review	Poster advert	☒
	Flyer	☒
	Email circulation	☒
	In-person approach	☒
	Website	☒
	Social media (e.g. twitter, Facebook)	☒
	Snowball sampling (recruiting through contacts of existing participants)	☒
	Newspapers	☐
	Research recruitment sites (e.g. Prolific Academic, Amazon Turk)	☐
	Existing departmental contacts or volunteer database	☒
	Other (please specify)	☐

**8. How will potential participants be identified and approached?**	Information about the study and poster will be shared in social media (including Facebook groups [with permission of the group moderator] i.e. Community and parents groups, for example [Oxford] Community, [Oxford] Parents & Careers, paediatric organisations, charities and research groups), twitter (personal

For peer review only - http://bmjopen.bmj.com/site/about/guidelines.xhtml
For peer review only

	accounts, research group account and organisations we are collaborating with – NIHR Children and Young People (CYP) Medical Technology and In-vitro Co-operative (MIC)/ NIHR Community Health (CH) MIC), blogs.<br><br>Newsletters and mailing lists could be used to share information and poster. Poster will include detail of £20 remuneration in the form of electronic gift vouchers will be offered for time taken to participate in interview.<br><br>These will include the University Department's mailing list charities and baby groups, paediatric medical organisations and, partners from the NIHR MICs will share information in their website and newsletter. The emails will be sent on behalf of the researchers conducting this study. We will also publish information of the study in our website and rely on word of mouth of participants.<br><br>Participants can express their interest in taking part of the study by contacting the researcher by email or phone. We will also include a form on the website for people to express their interest. Any potential participant expressing interest in taking part in the study will be contacted by email or telephone. Potential participants will be directed to our website where they can download the participant information sheet and consent form. Participants wishing to take part will be asked to return the consent form and participant information sheet signed. Participants wishing to take part, will be invited (by email or phone) to take part in the co-design workshop. Information and invite in the co-design workshop will use the same medium as the interviews did for distribution.<br><br>Participants will be given options regarding the timing, format, and venue (if face-to-face) for the interview will be given. The participant will have the opportunity to ask any questions either by email or telephone. They may choose to decline any further contact; in which case they will be reassured that this will in no way impact upon their current or future working.<br><br>The workshop will have a set date, participants will be asked to register their attendance in advance.
**9. Will informed consent be obtained from the research participants or their parents/ guardians?** If not, please explain why not.	Verbal consent will be taken at the start of interviews and co-design workshop. Before the interview/workshop starts, we will explain the study to the participant and go through the consent form.<br><br>Written informed consent will be obtained by means of participant dated signature and dated signature of the person who presented and obtained the Informed Consent.<br><br>Written consent will be sought participants will be given the option to fill the consent themselves and emailing it to us, they can also complete it through a GDPR compliant platform (https://www.smartsurvey.co.uk/) or researcher can fill them on their behalf and email a password-protected copy and retaining a copy for the records. For interviews being done by phone, form will be sent by email, or if not possible, oral consent will be sought.<br><br>Participant will be able to ask any questions or withdraw at any point without any explanation.
**10. For each activity or group of participants, explain how informed consent will be obtained from the participants themselves and/or their**	Verbal consent will be taken at the start of the interview and workshop. Study will be explained and the researcher will go through the consent form with the participant.<br><br>Written consent will be sought prior to interviews and co-design workshop are taking place. As interview/workshop will be conducted remotely (either online or

For peer review only - http://bmjopen.bmj.com/site/about/guidelines.xhtml

**parents/guardians, if applicable. How will their consent be recorded?**	by phone) participants will be given a few options to fill the consent form; the option to fill the consent themselves and emailing it to us, they can fill it online through a GDPR compliant software (https://www.smartsurvey.co.uk/) or researcher can fill them on their behalf and email a password-protected copy and retaining a copy for the records. A copy of the consent form will be made and stored safely in password protected file.<br><br>Participants will be given the opportunity to ask questions or withdraw from study. If participants are happy to proceed, interviews will be arranged online or by telephone at their convenience. A date will be set for the workshop, participants can register their interest to participants through the website. We will be available to reply to any questions or concerns that the participant may have at any point during this process.

SECTION E. RESEARCH METHODOLOGY

1. Please mark 'X' against the methods that will be used in your research

Ensure you address each method you will use in your informed consent documents and on this form

Use of casual or local workers (e.g. interpreters)	☐	Audio recording of participant	☒
Interview (refer to guidance in BPG 10: Conducting research interviews)	☒	Video recording of participant	☐
Focus group	☐	Photography of participant	☐
Participant completes questionnaire in hard copy	☐	Physiological recording from participant	☐
Participant completes online questionnaire or other online task (refer to guidance in BPG 06: Internet-mediated research)	☐	Taking a sample of blood or other bodily fluid from a participant	☐
Use of social media to recruit or interact with participants (refer to guidance in BPG 06: Internet-mediated research)	☒	Participant observation	☐
Analysis of existing records	☐	Covert observation	☐
Participant performs verbal or aural task	☒	Systematic observation	☐
Participant performs paper and pencil task	☒	Observation of specific organisational practices	☐
Participant performs computer based task	☒	Other (please specify below)	☐
Measurement/recording of motor behaviour	☐		

2. Provide a lay description of the research design and methods. In particular, describe clearly what participants in the research will be asked to do.

Interviews:

Before interview starting, the information sheet will be reviewed, and any questions addressed.

- Participants will be asked to provide their informed consent to participation prior to data collection. Participants will be explained to that their participation is voluntary and they can end or withdraw from study at any point without any explanation.

For peer review only - http://bmjopen.bmj.com/site/about/guidelines.xhtml

- A date for the interview to take place will be scheduled. Participants will have a choice of date, time and place of interview (online or telephone).
- Sessions will last between 40-60 min. Each participant will have one session. Consent will be asked in case follow-up is required in any of the questions.
- A qualitative methodology will be used given that it is appropriate to explore people's knowledge, choices, and decision. Data collection and analysis will be guided by the principles of grounded theory which allows adjustments to questions and sampling.
- Semi-structured questionnaires with open-ended questions will be used for the interviews. A topic guide will be used to conduct the interviews. This will allow flexibility in the order and wording of the questions. Additionally, questions will be amended based on on-going interviews, to explore emerging ideas and themes and to include new participants as participants for interview (theoretical sampling).

The topic guide will be divided into three parts, a warmup section that will include personal information and questions to get to know the person being interviewed and their relationship to urine collection. Then the main questions section, which will determine experiences, stories and opinions about urine collection methods. Finally, the wrap-up questions, to give the opportunity to ask any questions to us.

The open-ended questions will be used to probe the different areas of urine collection we are interested in. The interviews will explore the procedure, i.e., who uses it, device used, procedure (who collects the urine, how long it takes, final sample), post-collection feedback and process, patient follow-up.

- Interviews will be audio recorded and transcribed. If only audio is not possible (during online meeting),participant will be asked to turn off their video off during the interview..

Co-design Workshop

- Participants will be invited to take part in the workshop. They can express their interest by filling a registration form on the website.
- Participant will be able to download the consent and participant information form from the website. And will be able to contact the researchers with questions using the contact information provided in the participant information sheet.
- Participants will be asked to provide their informed consent to participation before the workshop starts. Participants will be explained to that their participation is voluntary and they can end or withdraw from study at any point without any explanation. They will also have the opportunity to ask questions.
- Participants will be divided in groups, where they can discuss urine collection ideas and brainstorm new solutions. This can be by drawing/writing/verbal explanation or making something with available materials. At the end each group will be able to present the summary of their ideas to the rest of the participants and organisers.
- If workshops are held online. An online worksheet will be set up and teams will work on their ideas there. Teams will be asked to present their ideas at the end of the workshop.
- Notes will be taken and online worksheets will be kept by the research team for analysis and further work. No video or audio recording will be taken.
- Session will last between 2.5-3h

For peer review only - http://bmjopen.bmj.com/site/about/guidelines.xhtml

**3. Will the research include any audio, video or photographic recordings?**	
Yes. Participants who don't wish to be recorded could still take part. In this case researcher will take notes during the interviews.	
**4. Biological sample handling**	
No biological samples will be taken as part of the study.	
**5. Please detail all expenses or gifts that will be offered to participants.**<br>Guidance is available in Best Practice Guidance: 05 Payments and incentives in research.	
RNo remuneration will be given for participation in the study, in the form of £20 electronic gift vouchers for time taken to participate in interviews..	

For peer review only

SECTION F. ETHICAL CONSIDERATIONS

For guidance on ethical issues, please see http://researchsupport.admin.ox.ac.uk/governance/ethics/resources

(N.B. To complete, double click on the check boxes and select 'checked')

**1. Will the research involve any participants considered vulnerable in the context of the research (e.g. children, elderly, prisoners)?**<br>**If yes,** please describe how they are defined as vulnerable and detail any CUREC Approved Procedures or guidance that will be applied to the research (for current documents and templates see https://researchsupport.admin.ox.ac.uk/governance/ethics/resources).<br>**If yes, and you cannot apply any Approved Procedure**, please cease completion of this form – a CUREC 2 application is required	Yes ☐	No ☒
**2. Will unequal relationships exist between participants and those obtaining informed consent?**<br>**If yes,** describe the nature of the unequal relationship and how arising ethical issues will be addressed	Yes ☐	No ☒
**3. Will the research involve questions and/or discussions of contentious and/or sensitive issues (e.g. information relating to ethnicity, political opinions, religious beliefs, physical/mental health or sexual life)?**<br>**If yes,** please justify why this is required and provide a copy of the questionnaire raising the issues that will be used in your research.	Yes ☒	No ☐
Healthcare professionals:<br>We know urine collection methods and procedure are not consistent throughout different clinical practices or clinicians. We would like to understand what drives the decision making. We know different countries and culture		

For peer review only - http://bmjopen.bmj.com/site/about/guidelines.xhtml

have different acceptability to invasive or non-invasive procedures, so would like to test whether ethnicity plays a role.

Parents:

We would like our study to be diverse and include people from different social-economic backgrounds and ethnic backgrounds. In order to quantify the success rate of these goals, and report them, we will ask parents/carers to state their ethnicity and questions that will allow us to estimate their social-economic position.

Workshop:

In order to be able to report who took part in this study, we will ask demographic questions. Participants will have the chance of not answering if they don't feel comfortable doing so.

**4. Will the research involve deliberate deception of participants outside the scope of CUREC Approved Procedure 07?**<br>**If yes**, please cease completion of this form – a CUREC 2 application is required<br>**If no,** please either give details of the deception or state that no deception will be involved	Yes ☐	No ☒
**5. Could the proposed research affect your own physical and/or psychological safety as a researcher?**<br>**If yes,** describe how you will manage this.	Yes ☐	No ☒

6. How will you ensure the research is conducted according to the details given in this form?

Members of the research team will meet weekly to discuss progress or issues and themes that are arising from the interviews in order to make decision about the continuity and direction of interviews. Moreover, these discussions will also support the data analysis. And if needed, another person will be review recordings to validate findings.

Any students involved in the study will form part of the research team and will present findings on weekly basis. Students will be supported in their analysis by senior researchers.

Procedures will be discussed during meetings. We have involved people with experience in qualitative studies, who will support the process. Additionally, senior members of the research team will be able to join interviews and analysis, in order to check procedures are followed.

If any participant has questions, they will be able to ask the research team directly. They will also be able to reach the principal investigator if they feel appropriate. We will acknowledge reception and will give an indication of how it will be dealt with. Any serious complaints will be forwarded to the Chair of the Research Ethics Committee at the University of Oxford (ethics@medsci.ox.ac.uk).

7. Please give details of any other research-specific ethical and/or safety considerations

None

8. How do you propose to deal with / handle any incidental findings?

Not applicable

For peer review only - http://bmjopen.bmj.com/site/about/guidelines.xhtml

**9. Will any data or information from this study be provided to individual participants?**
No. The study aims to explore and validate challenges faced during urine collection. Therefore, there are no individual results to be provided.

SECTION G. OTHER CONSIDERATIONS

**1. Is any part of this research being conducted overseas?**<br>**If yes,** please give details below. Ensure you complete and submit a travel risk assessment form to your departmental safety officer. (This is necessary to ensure the travel/fieldwork is covered by the University's travel insurance – see http://www.admin.ox.ac.uk/finance/insurance/travel/)	Yes ☐	No ☒
**2. Does your research raise issues relevant to the Counter-Terrorism and Security Act (the Prevent Duty), which seeks to prevent people from being drawn into terrorism?**<br>**If yes,** please say how you plan to address any related risks. Please see advice on this on our Best Practice Guidance Web Page.	Yes ☐	No ☒

SECTION H. DATA MANAGEMENT AND HANDLING

All information provided by participants is considered **research data** for the purpose of this form. Any research data from which participants can be identified is known as **personal data**; any personal data which is sensitive is considered **special category data**.

Management of personal data, either directly or via a third party, must comply with the requirements of the General Data Protection Regulation (GDPR) and the Data Protection Act 2018, as set out in the University's Guidance on Data Protection and Research.

In answering the questions below, please also consider the points raised in the Data Protection Checklist and whether, for higher-risk data processing, a separate Data Protection Impact Assessment may also be required for the research. Advice on research data management and security is available from Research Data Oxford and your local IT department. Advice on data protection is available from the Information Compliance team.

1. Please mark 'X' against the data you will collect for your research

Screening documents	☐	Audio recordings	☒
Consent records including participant name or other identifiers (e.g. written consent forms, audio-recorded consent, assent forms)	☒	Video recordings	☐

For peer review only - http://bmjopen.bmj.com/site/about/guidelines.xhtml

Consent obtained anonymously (e.g. via online survey)	☐	Transcript of audio/video recordings	☒
Opt-out forms	☐	Photographs	☐
Contact details for the purpose of this research only	☒	Information about the health of the participant (including mental health)	☐
Contact details for future use (guidance)	☒	Physiological test results / measurements	☐
Field notes	☒	MRI scans	☐
Task results (e.g. questionnaires, diary completion)	☐	IP addresses (refer to Best Practice Guidance 09: Data collection, protection and management for guidance)	
Data already in the public domain.<br>Specify the source of the data:	☐	Other (please specify below)	☐
Previously collected (secondary) data	☐		

2. How and where will each type of data be stored whilst the research is ongoing (until the end of all participant involvement)?

List each type of data selected above, and explain how each will be physically transferred (including movement/sharing of audio files, paper records, electronic downloads etc.) from where it is collected to a suitable storage site (e.g. Nexus365 OneDrive for Business, SharePoint, University servers). State the storage location for each. Do not store unencrypted data in freely available cloud services or unprotected USB drives.

Refer to Best Practice Guidance on data collection, protection and management (BPG09).

Hard copies of data (consent forms and field notes) will be placed in a folder and transferred to an office at the Engineering Science Department as soon as possible. In the Engineering Science Department, consent forms will be stored in a locked filling cabinet. When, despite efforts, it is not practical to immediately store data in this location, a lockable filling cabinet in the researcher's home will be used and will be transferred to the Engineering Science Department as soon as possible.

All electronic data (typed field notes, audio recordings and personal data including contact details) will be transferred from the researcher's password protected device to a password-protected drive within the University network. The audio recording held by the researchers will then be deleted once the interview audio has been transcribed. These recordings will be securely transferred using the university file sharing system or Nexus OneDrive for business. Workshops work documents will be de-identified.

Identifying information from audio recordings will be removed from the transcripts. Transcription will be done by the researcher or approved University transcriber. De-identified transcripts may be uploaded into NVIVO 12 which will accessed by the research team on password-protected computers. This de-identified data may also be made available to other researchers.

The transcription will be stored as a Word file on encrypted computers within the University network.

3. Will you use a unique participant number on research data instead of participant name?

If yes, state whether or not you will retain a list of participant names against numbers (pseudonymisation via a linkage list).

Where will the list be stored, and when will it be destroyed?

Yes. Data identifiers will be labelled and stored securely and separately from the research data, on password protected computers. We will destroy participants information once research has been completed and published.

For peer review only - http://bmjopen.bmj.com/site/about/guidelines.xhtml

**4. Who will have access to the research data?**
Researchers listed on this form will have access to the research data and other student/researchers working on the study. Access will be granted to the MS IDREC for the purposes of monitoring and/or audit of the research.<br>De-identified data may be made available to other researchers.
**5. If research data is to be shared with another organisation, how will it be transferred / disclosed securely?**
Any data will be shared securely using the university file sharing system (OxFile or Nexus365 OneDrive Business system) and returned the same way. The data will be stored in password-protected computers within their institutions.
**6. When and how will identifiable data be destroyed or deleted?**<br>N.B. If any identifiable data will be retained beyond the end of the study and/or indefinitely, please state what data this is, and the reasons for retention (e.g. contact details for future studies; photos used in publication). This must be clearly stated on participant information, and specific consent obtained.
All identifiable data will be removed from the transcript.<br>Unique participants number will be used. Audio recordings will be destroyed after publication.<br>Consent records and de-identified transcripts and worksheets will be kept 3 years after publication.<br>Personal data will be deleted from servers after the analysis and publication has been completed.

**7. Please confirm that you will store other (non-identifiable) research data safely for at least 3 years after final publication or public release and adhere to any additional research funder policies.**<br>For more information about the University policies, please see the University's web pages on research data management.<br>**If 'Yes'**, please give details of who will store the data and on storage format, location and security.<br>**If 'No'**, please provide further details.	Yes ☒	No ☐
Non-identifiable data will be stored in a password-protected computer within the University network at the PI's department.		

SECTION I. PUBLICATION AND DISSEMINATION OF RESULTS	
**1. How will you disseminate and feedback project outcomes at the end of the research?**	Study finding will be disseminated though publications in peer-reviewed journals, conferences and other articles such as for the Community Health MIC, Children and Young People MIC. Public journals or seminars.

For peer review only - http://bmjopen.bmj.com/site/about/guidelines.xhtml

	We will aim to make these findings available to the scientific and wider community by following the Open Science guidelines.

For peer review only

For peer review only - http://bmjopen.bmj.com/site/about/guidelines.xhtml

DECLARATIONS AND SIGNATURES

In providing signatures, the MS IDREC Secretariat will accept either:

Option 1: Email confirmations sent from a University of Oxford email address. Separate emails should be sent by each of the relevant signatories as outlined below, indicating acceptance of their responsibilities.

Option 2: That the form be fully-signed with handwritten (wet-ink) signatures. Please scan these and the rest of the form pages to create a single PDF document and email to us.

PRINCIPAL INVESTIGATOR (AND STUDENT IF APPLICABLE)

I/We, the researcher(s):

- Understand our responsibilities as outlined on this form and in the CUREC glossary and guidance
- Agree to start this research only after obtaining approval from the MS IDREC;
- Understand that the Principal Investigator must ensure that all researchers are suitably qualified and trained to conduct the research described, or are appropriately supervised until deemed qualified/trained;
- Agree to provide additional information as requested by the MS IDREC before approval is secured and as research progresses;
- Agree to maintain the confidentiality of all data collected from or about participants;
- Agree to notify the MS IDREC in writing immediately of any proposed change to the research, and await approval before proceeding with the proposed change;
- Agree to notify the MS IDREC if the Principal Investigator changes and supply the name of the successor;
- Will use the data collected only for the research for which approval has been given;
- Will grant access to data only to authorised persons; and
- Have made arrangements to ensure that personal data collected from participants will be held in compliance with the requirements of the GDPR and the Data Protection Act 2018.

**Principal Investigator (Name)**	Jeroen Bergmann
**Principal Investigator (Signature)**<br>Pasted images of signatures cannot be accepted	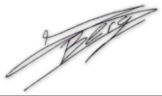
**Date**	30/06/2021
**Student (Name)**	
**Student (Signature)**<br>Pasted images of signatures cannot be accepted	
**Date**	

For peer review only - http://bmjopen.bmj.com/site/about/guidelines.xhtml

ACCEPTANCE BY HEAD OF DEPARTMENT/FACULTY OR DESIGNATED NOMINEE*

*Another senior member of the department may sign where the head of department is the Principal Investigator, or where the head of department has appointed a nominee. Example nominees include Deputy Head of Department, Director of Research, or Director of Graduate/Undergraduate Studies.

I have read the research proposal above. On the basis of the information available to me, I:

- consider the Principal Investigator/Supervisor and student researcher (if applicable) to be aware of their ethical responsibilities in regard to this research;
- am satisfied that the proposed design and scientific methodology are sound; the research has been subject to appropriate peer review and is likely to contribute to existing knowledge and/or to the education and training of the researcher(s) and that it is in the public interest.

For peer review only

**Head of Department or designated nominee (Name)**	
**Head of Department or designated nominee (Signature)**<br>Wet-ink signature (not pasted electronic image)<br>*or*<br>The Head of Department/nominee can send an email (including PI name and study title) to ethics@medsci.ox.ac.uk confirming the above	
**Date**	

For peer review only - http://bmjopen.bmj.com/site/about/guidelines.xhtml

Study Title: Exploring perspectives, priorities, and solutions of urine collection: A qualitative user centric study to understand practices and gain feedback on from healthcare professionals and parents/carers
Document Title: Topic Guide Healthcare Staff
Date and Version Number: 20-Oct-2021, V0.1
CUREC Reference Number: R77332/RE001

DEPARTMENT OF ENGINEERING SCIENCE
UNIVERSITY OF OXFORD

Interview questions

This is a semi-structured interview. The questions below provide a guide and are not fixed. The interviewee can talk freely during the interview and the conversation will guide which questions will be asked next. The interview has 3 stages: warm-up questions, main questions, and wrap-up questions.

Introduction

Reassure interviewee that this is an interview to understand practices and knowledge around urine collection in order to design new solutions including technology, rather than judging or evaluating practices of individual.

Healthcare Professionals:

Warm-up questions

Interviewees will be asked to complete these questions in writing. Demographic questions will have an option "prefer not to say". Interviewer will remind participants that they do not need to answer any question they don't wish to answer.

All Healthcare Professionals

- Tell us about yourself
  - which hospital do you work in?
  - what is your role?
  - how long you have been in the role?
- Demographic questions
  - Age
  - Gender
  - Ethnicity
- How did you hear about this study?

If Medical Practitioners/Nurses

- About position
  - Main location of practice
  - Country of medical or nursing degree if applicable
  - Years of working in as current specialty (ie. As GP / in ED / Admin)
  - Training level
  - Do you know how many children under 2 would have a urine collection a week?
  - Specialised in paediatrics
  - Additional postgraduate qualification in paediatrics or child health
- Number of full-time equivalent GPs at clinic/number of doctors in ED
- Number of full-time equivalent nurses
- Type of clinic (private, mixed, NHS, other)
- What is your role related to urine collection?

For peer review only - http://bmjopen.bmj.com/site/about/guidelines.xhtml

Main questions

If Medical Practitioners/Nurses

Deep dive into the urine collection process:

- In the hospital you work in / have worked in, is there a preference for a particular urine collection method?
- What is your experience carrying out urine collection in infants? Have you used all methods?
- How often do you perform urine collection in children? / (in a day/week how many urine samples are collected?)
- Think about a recent experience carrying out/ordering/supervising/assisting a urine collection in an infant
  - Age of patient
  - Physical and emotional condition of patient / parent
  - Where was the collection done? Home, hospital, GP practice?
  - Who ordered the urine collection?
  - Where did the collection take place?
  - How many people were involved in the procedure?
  - Who carried out the collection?
  - How long did it take?
  - Any feedback?
  - Follow-up of patient
- Tell me about how would you go about asking parents to obtain a urine sample from their child?
  - Instructions:
    - ~~Have you been taught how to give~~Do you provide urine collection instructions for parents?
      - If no: why not? (lack of time, lack of knowledge, don't think it's necessary)
      - If yes, what type of instructions do you provide?
        - Do you think this is normal practice with colleagues? (allude to the fact that most parents have mentioned not to get instructions, would they agree?.
          - Why do you think that is?
    - ~~Did you share this information with parents?~~
  - Procedure explanation
  - Why are you collecting the sample?
- Who decides to get a urine sample?
  - If you, when would you get a urine sample?. Is this process clear?
- What is your perception of non-invasive urine methods as a diagnostic procedure?
- What is your opinion about the urine collection devices available?
  - Invasive vs. non-invasive
  - What is the determining factor when choose a particular option?
- The NICE guidelines in UK suggest using the clean catch method. In your opinion is this the most common method used?
- Do you or your colleagues ever use voiding stimulating methods for clean catch (i.e. Quick-wee method, bladder-tapping method)?
  - If yes, what is the practice mostly used and your experience in terms of success rate, usefulness, difficulty
  - If not, why not?
- What do you think are the pros and cons of current urine collection devices/methods, if any?

Formatted

Formatted: Font: (Default) +Body (Calibri), 11 pt

Commented [MA1]: Here, trying to see, if they feel is an easy and valid method to diagnose an infection or if it's just a check list procedure.

Formatted: Font: (Default) +Body (Calibri), 11 pt

Formatted: List Paragraph, Bulleted + Level: 1 + Aligned at: 0.63 cm + Indent at: 1.64 cm

For peer review only - http://bmjopen.bmj.com/site/about/guidelines.xhtml

   - Re-visits
   - Misdiagnosis
   - Contamination
   - Usability
- Do you know of other novel urine collection devices?
- Having considered everything mentioned before,
   - what would the ideal process look like from beginning to end?
   - Where can/does the process go wrong?

If Bio-analyst

Deep dive into the urine collection processes:

- Tell me about the work you do in regard to urine analysis in infants?
- How many urine collections happen in a month?
- How reliable are urine samples of infants for diagnosis of infections?
   - Do you have information of the method used for urine collection?
      - If yes, what do you think about invasive and non-invasive methods of urine collection?
      - How does clean catch compare?
      - Do you think there are other practices that reduce contamination rates
- Is retesting a frequent occurrence?
   - How much do you think this cost?
   - In your opinion what drives the use of one method or another?
- Having considered everything mentioned before,
   - what does the ideal process look like from beginning to end?
   - Where can/does the process go wrong?

If admin staff

Deep dive into the urine collection processes:

- Tell me about your experiences with urine collection in infants?
- How many urine collections happen in a month?
- What is the cost?
   - What are the resources used for a urine collection in infants
      - Duration for a collection
      - Use of room
      - Staff
   - How does clean catch compare to other methods?
   - In your opinion what drives the use of one method or another?
- The NICE guidelines in UK suggest using the clean catch method. In your opinion is this the most common method used?
   - Have you ever heard of voiding stimulating methods for clean catch (i.e. Quick-wee method, bladder-lumbar method)?
      - If yes, what is the success rate?
- Are you aware of contamination rates in different urine collection methods?
   - How does this affect cost?
- Do you know of other novel urine collection devices?
- Having considered everything mentioned before,
   - what does the ideal process look like from beginning to end?
   - Where can/does the process go wrong?

Wrap-up questions

For peer review only

For peer review only - http://bmjopen.bmj.com/site/about/guidelines.xhtml

End with asking them if they have any questions, ensuring they are comfortable with everything that has been said, and whether they would be open to further interviews in the future:

- Are there any questions you think I should have asked? Is there anything you would like to add?
- Who/where else do you think it might be interesting for us to interview? - How come? (because of different dynamic, scale and scope of work etc).
- If I had any quick questions arise from reviewing my notes today, would it be possible to give you a quick call/email?
- Would you been open to further interviews from us in the future as we continue to develop this tool?

Do you have any questions from us, would you like to know more about our urine collection project?

For peer review only

Study Title: Exploring perspectives, priorities, and solutions of urine collection: A qualitative user centric study to understand practices and gain feedback on from healthcare professionals and parents/carers
Document Title: Topic Guide Parents/Carers
Date and Version Number: 20-Oct-2021, V0.1
CUREC Reference Number: R77332/RE001

Interview questions

This is a semi-structured interview. The questions below provide a guide and are not fixed. The interviewee can talk freely during the interview and the conversation will guide which questions will be asked next. The interview has 3 stages: warm-up questions, main questions, and wrap-up questions.

Reminder of how we will use the data:

> We will record the interview. This will be transcribed, and all identifiable information will be erased from transcription and stored safely within the Engineering Department. Recordings will be erased.

Introduction

Reassure interviewee that this is an interview to understand what works and what doesn't work of current urine collection methods so we can design technology that helps parents and clinicians.

Warm-up questions

We will give demographic questions to interviewee for them to fill. The option of prefer not to say will be given. Interviewer will remind participant that they do not need to answer any question they prefer not to answer.

- Tell us about yourself;
  - Who do you care for?
  - How many children do you have?
  - Demographic questions
    - Age
    - Gender
    - Ethnicity (give options from a list)
    - Social demographics:
      - Post code
      - How many people live in the house?
      - Employment status (full time / part time / stay at home parent / unemployed)
      - Number of dependents
    - Education level
  - How did you hear about this study?

Main questions

Deep dive into the urine collection process:

- Tell me about your experiences with urine collection in infants and toddlers?
  - When did it last happen?
  - Where did the urine collection occur? i.e. hospital, home, GP

For peer review only - http://bmjopen.bmj.com/site/about/guidelines.xhtml

- If you sought medical attention, what were the symptoms your child had that worried you?
  - Tell me about how you were told to get the sample
    - How was the urine sample collected?
    - Were you given instructions? Do you remember them (the instructions you were given)? → Urine Collection Method
    - Who gave them to you?
    - Did someone explain why a urine sample was needed?
    - Was there any leaflet or other information that helped with the process?
      - If yes, do you remember any helpful information?
  - How did you find the process of urine collection in your child?
    - What were the challenges?
    - What worked well?

- When did this last happen?
- Did this happen with only one or many of your children? Does it happen often?
- How often do you perform urine collection in children?
- Are you aware that there are different urine collection methods?
  - Have you previously used other methods?
  - If you used other ways of collecting urine, which method did you prefer?
    - Why?
  - Did you have any worries about some of the collection methods?

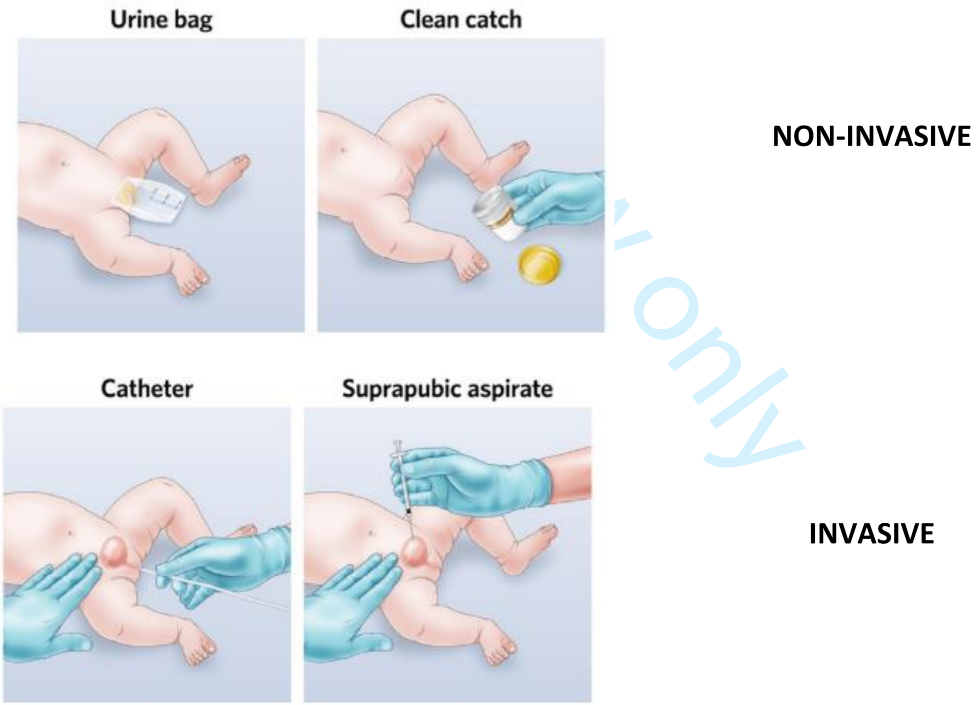

Figure 1. Images by Dr. Jonathan Kaufman and Bill Reid, Royal Children's Hospital. Kaufman et al, BMJ, 2017. 357: 16-19.

- What is your opinion about the urine collection devices available?

For peer review only - http://bmjopen.bmj.com/site/about/guidelines.xhtml

  - Invasive vs. non-invasive, i.e. a sample requiring a nurse/doctor placing a urinary catheter versus getting the child to sit on a potty
  - If there was more than one option, were you given a choice?
- The NICE guidelines in UK suggest using the clean catch method. In your opinion was this the method used for your child.
  - Have you ever heard of ways to speed up collecting urine samples (eg gently tapping the bladder)?
    - If yes, did it work? / How long did it take?
- Are you aware that some urine samples are unreliable because they end being contaminated with bacteria (bugs) commonly found on our skin?
- Do you know of other urine collection devices?
- In your opinion:
  - what would the ideal process look like from beginning to end?
  - Where can/does the process go wrong?

Wrap-up questions

End with asking them if they have any questions, ensuring they are comfortable with everything that has been said, and whether they would be open to further interviews in the future:

- Would you like to add anything that we didn't ask about?
- Do you know anyone else that has gone through urine collection in a non-toilet trained infant that might be interested in participating?
- If I had any quick questions arise from reviewing my notes today, would it be possible to give you a quick call/email?
- Would you been open to further interviews from us in the future as we continue to develop this tool?

For peer review only - http://bmjopen.bmj.com/site/about/guidelines.xhtml

COREQ (COnsolidated criteria for REporting Qualitative research) Checklist

A checklist of items that should be included in reports of qualitative research. You must report the page number in your manuscript where you consider each of the items listed in this checklist. If you have not included this information, either revise your manuscript accordingly before submitting or note N/A.

Topic	Item No.	Guide Questions/Description	Reported on Page No.
**Domain 1: Research team and reflexivity**			
Personal characteristics			
Interviewer/facilitator	1	Which author/s conducted the interview or focus group?	
Credentials	2	What were the researcher's credentials? E.g. PhD, MD	
Occupation	3	What was their occupation at the time of the study?	
Gender	4	Was the researcher male or female?	
Experience and training	5	What experience or training did the researcher have?	
Relationship with participants			
Relationship established	6	Was a relationship established prior to study commencement?	
Participant knowledge of the interviewer	7	What did the participants know about the researcher? e.g. personal goals, reasons for doing the research	
Interviewer characteristics	8	What characteristics were reported about the inter viewer/facilitator? e.g. Bias, assumptions, reasons and interests in the research topic	
**Domain 2: Study design**			
Theoretical framework			
Methodological orientation and Theory	9	What methodological orientation was stated to underpin the study? e.g. grounded theory, discourse analysis, ethnography, phenomenology, content analysis	
Participant selection			
Sampling	10	How were participants selected? e.g. purposive, convenience, consecutive, snowball	
Method of approach	11	How were participants approached? e.g. face-to-face, telephone, mail, email	
Sample size	12	How many participants were in the study?	
Non-participation	13	How many people refused to participate or dropped out? Reasons?	
Setting			
Setting of data collection	14	Where was the data collected? e.g. home, clinic, workplace	
Presence of non-participants	15	Was anyone else present besides the participants and researchers?	
Description of sample	16	What are the important characteristics of the sample? e.g. demographic data, date	
Data collection			
Interview guide	17	Were questions, prompts, guides provided by the authors? Was it pilot tested?	
Repeat interviews	18	Were repeat inter views carried out? If yes, how many?	
Audio/visual recording	19	Did the research use audio or visual recording to collect the data?	
Field notes	20	Were field notes made during and/or after the inter view or focus group?	
Duration	21	What was the duration of the inter views or focus group?	
Data saturation	22	Was data saturation discussed?	
Transcripts returned	23	Were transcripts returned to participants for comment and/or	

For peer review only

Topic	Item No.	Guide Questions/Description	Reported on Page No.
		correction?	
**Domain 3: analysis and findings**			
Data analysis			
Number of data coders	24	How many data coders coded the data?	
Description of the coding tree	25	Did authors provide a description of the coding tree?	
Derivation of themes	26	Were themes identified in advance or derived from the data?	
Software	27	What software, if applicable, was used to manage the data?	
Participant checking	28	Did participants provide feedback on the findings?	
Reporting			
Quotations presented	29	Were participant quotations presented to illustrate the themes/findings? Was each quotation identified? e.g. participant number	
Data and findings consistent	30	Was there consistency between the data presented and the findings?	
Clarity of major themes	31	Were major themes clearly presented in the findings?	
Clarity of minor themes	32	Is there a description of diverse cases or discussion of minor themes?	

Developed from: Tong A, Sainsbury P, Craig J. Consolidated criteria for reporting qualitative research (COREQ): a 32-item checklist for interviews and focus groups. *International Journal for Quality in Health Care*. 2007. Volume 19, Number 6: pp. 349 – 357

Once you have completed this checklist, please save a copy and upload it as part of your submission. DO NOT include this checklist as part of the main manuscript document. It must be uploaded as a separate file.

For peer review only
